# Electrophilically Activated Nitroalkanes in Double Annulation of [1,2,4]Triazolo[4,3-*a*]quinolines and 1,3,4-Oxadiazole Rings

**DOI:** 10.3390/molecules26185692

**Published:** 2021-09-20

**Authors:** Alexander V. Aksenov, Nikita K. Kirilov, Nicolai A. Aksenov, Dmitrii A. Aksenov, Elena A. Sorokina, Carolyn Lower, Michael Rubin

**Affiliations:** 1 Department of Chemistry, North Caucasus Federal University, 1a Pushkin St., 355017 Stavropol, Russia; lyncheron@gmail.com (N.K.K.); naksenov@ncfu.ru (N.A.A.); mr.twos@mail.ru (D.A.A.); 2 Organic Chemistry Department, Peoples’ Friendship, University of Russia (RUDN University), 6, Miklukho-Maklaya St., 117198 Moscow, Russia; sorokina-ea@rudn.ru; 3 Department of Chemistry, University of Kansas, 1567 Irving Hill Road, Lawrence, KS 66045, USA; cmlower7@ku.edu

**Keywords:** nitroalkanes, heterocycles, annulation, cascade transformations

## Abstract

Nitroalkanes activated with polyphosphoric acid could serve as efficient electrophiles in reactions with amines and hydrazines, enabling various cascade transformations toward heterocyclic systems. This strategy was developed for an innovative synthetic protocol employing simultaneous or sequential annulation of two different heterocyclic cores, affording [1,2,4]triazolo[4,3-*a*]quinolines with 1,3,4-oxadiazole substituents.

## 1. Introduction

There is significant emphasis on the role of [1,2,4]triazolo[4,3-*a*]quinolines in modern-day drug discovery and medicinal chemistry. This privileged scaffold was utilized in the design of potent and selective aldosterone synthase inhibitors with antihypertensive activity [1], as well as prospective antitumor [2,3], anticonvulsant [4,5,6,7,8,9,10], anti-inflammatory [11,12], and antimicrobial [13,14] agents. It was also found that an introduction of a heterocyclic substituent at C-5 could allow for the preparation of chimeric scaffolds with improved biological activities. This strategy was employed in the development of cytotoxic and antiviral agents [15], as well as analgesic and anti-inflammatory drug candidates [16]. Recently, we described the preparation of novel antitumor agents with in vitro differentiation activity against neuroblastoma cancer cell lines [2]. These compounds were assembled via an unusual annulation reaction between electrophilically activated nitroalkanes [17] and 2-hydrazinylquinolines. We also reported on the cyclocondensation of nitroalkanes with acylhydrazides furnishing 1,3,4-oxadiazole rings [18,19]. With continuous SAR studies, we had a task of building a focused library of perspective “chimeric” antitumor drug candidates possessing both [1,2,4]triazolo[4,3-*a*]quinoline and 1,3,4-oxadiazole rings. An expeditious and concise synthetic method was needed to allow for a highly efficient installation of both heterocyclic cores in a single-pot fashion. In this report, we disclosed the results of these synthetic studies.

## 2. Results and Discussion

**Scheme 1:**
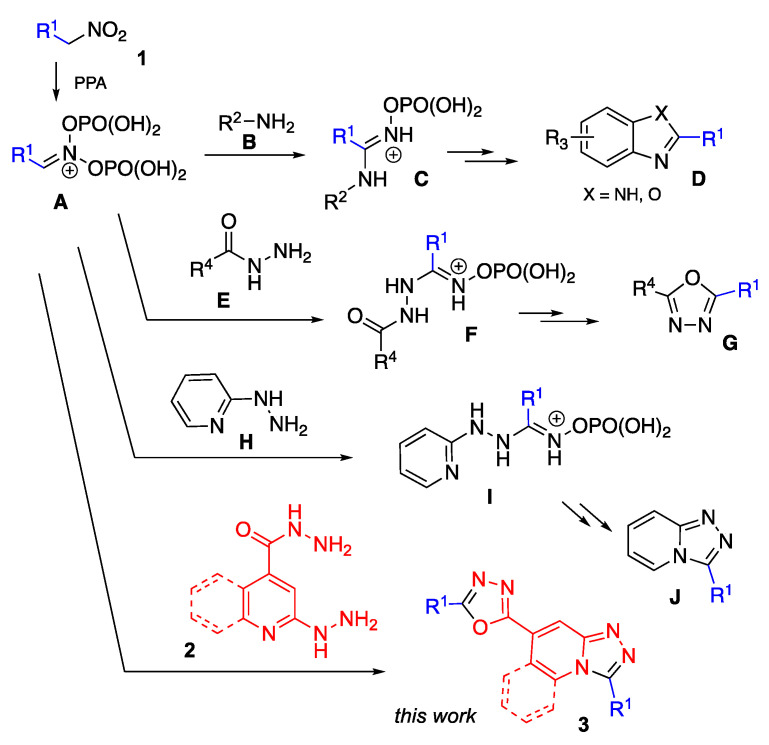
Electrophilic activation of nitroalkanes in synthesis of heterocycles.

As we previously reported, a highly electrophilic phosphorylated nitronate species **A** is generated upon the interactions between nitroalkanes **1** and polyphosphoric acid (PPA) (Scheme 1). These entities readily react with amines **B** to afford amidinium intermediates **C**, which can be used for the highly efficient assembly of imidazoles or oxazoles (**D**) [20,21], as well as imidazolines [22] and other nitrogen-based heterocycles [23,24]. Furthermore, the reaction with 2-hydrazinylpyridines **H** enacts formation of the corresponding (hydrazineyl)alkaniminium species **I**, which was found to undergo intramolecular cyclocondensations to obtain 1,2,4-triazolo[4,3-*a*]pyridines (**J**) (Scheme 1) [2]. Similarly, a mechanistically related reaction with acylhydrazides **E** proved useful for cyclization toward 1,3,4-oxadiazoles **G**. (Scheme 1) [18,19]. Remarkably, both latter reactions could be carried out under similar reaction conditions. This prompted us to pursue the idea of performing these reactions in a one-pot fashion en route to chimeric heterocyclic structures **3** (Scheme 1). To test the possibility of the simultaneous installation of these two heterocyclic moieties, we carried out the reaction of 2-hydrazineylisonicotinohydrazide (**2a**) in PPA in the presence of excess 1-nitropropane (**1a**, 4 equiv. used to compensate the loss due to evaporation). Initial tests were performed in 80% PPA (which corresponds to H_4_P_2_O_7_ composition). The reaction was carried out at 130 °C for 30 min, when it notably slowed down, most likely due to the loss of relatively volatile nitroalkane (bp 132 °C). Two more equivalents of **1a** was added, and the reaction was stirred for an additional 90 min to afford the desired 2-ethyl-5-(3-ethyl-[1,2,4]triazolo [4,3-*a*]pyridin-7-yl)-1,3,4-oxadiazole (**3aa**) with a 71% yield (Table 1, entry 1). To evaluate the influence of the medium, we also performed the same reaction in 87% PPA, which corresponds to polymeric HPO_3_ with lowered acidity, but enhanced anhydride activity. These parameters were modified aiming for an improved reaction performance or lowering of the reaction temperature. Indeed, even at 120 °C the yield of product **3aa** was notably higher (entry 2), and it reached 84% at 130 °C (entry 3). We also tested for the intermediate value of 85% P_2_O_5_ content in the reaction medium, which was found to be optimal for the featured process. With the increase in reaction temperature from 110–120 °C and then to 130 °C, the yield of **3aa** improved from 52–79% and then to 90%, respectively (entries 4–6). A further increase in temperature to 140 °C, however, was found to be detrimental, as it caused partial decomposition of the product (entry 7). 

**Scheme 2:**
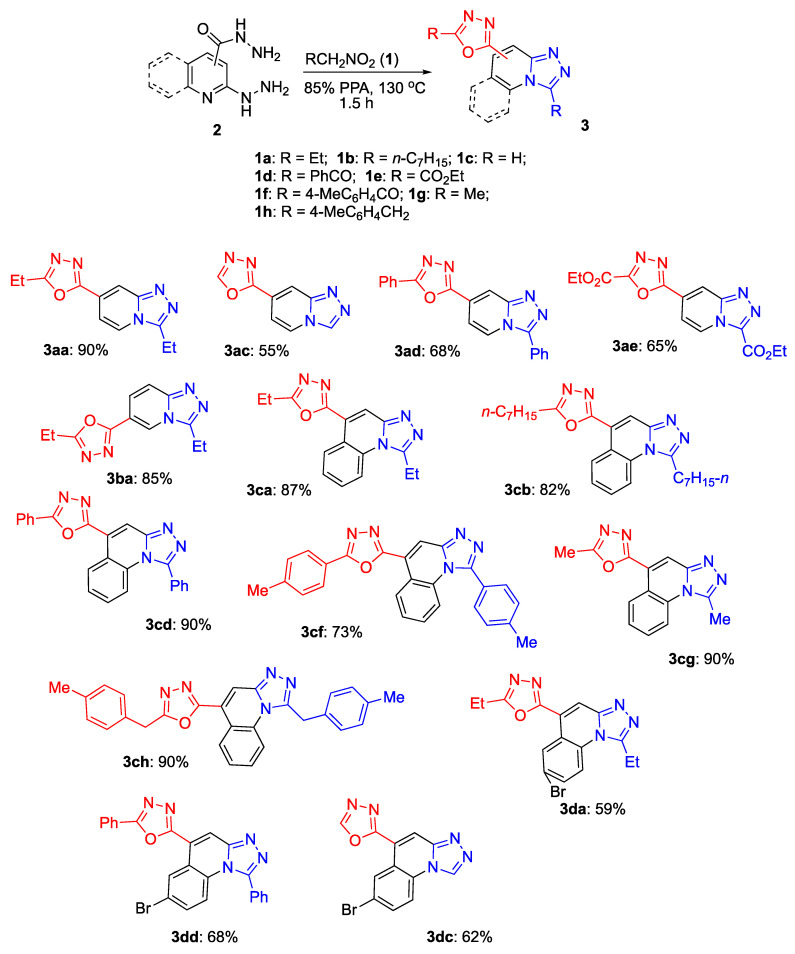
Preparation of [1,2,4]triazolo[4,3-*a*]quinolines with 1,3,4-oxadiazole rings.

**Scheme 3:**
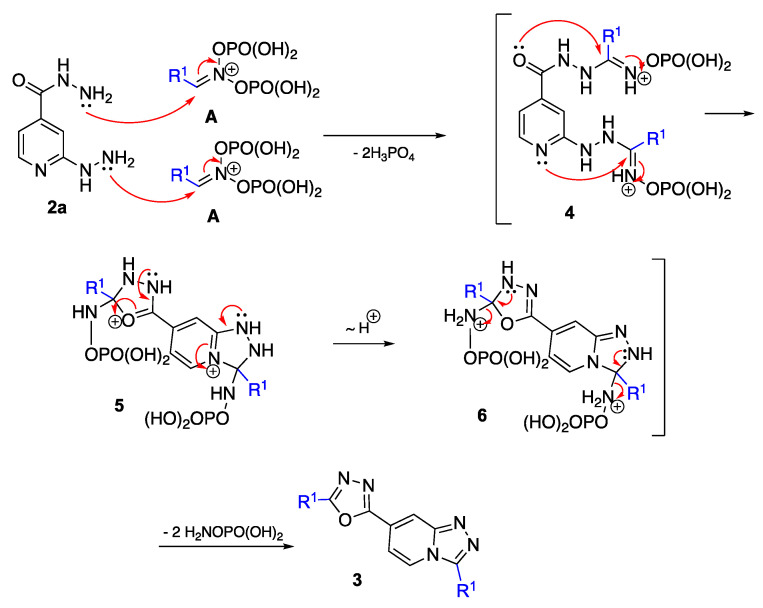
Mechanistic rationale involving electrophilic nitroalkanes.

With optimized reaction conditions in hand, we proceeded with the scope and limitation studies, the results of which are shown in Scheme 2. Nitromethane (**1c**) reacted smoothly, but the yields were somewhat lower due to partial loss of this reagent through its high volatility. The addition of nitromethane (up to 7 equivalents) to compensate for evaporation allowed for the isolation of polyheterocyclic products **3ac** and **3dc** in moderate to high yields (Scheme 2). Reactions involving three homologous nitroalkanes with higher boiling points, nitroethane (**1g**), 1-nitropropane (**1a**), and 1-nitrooctane (**1b**), proceeded much more efficiently, generally providing notably higher yields (Scheme 2). Furthermore, we managed to carry out the reaction of ethyl 2-nitroacetate, efficiently generating a bis-annulation product **3ae** with two newly introduced ester functionalities (Scheme 2). Moreover, *p*-tolyl(2-nitroethane) (**1h**) reacted smoothly, affording compound **3ch** in excellent yield (Scheme 2). A putative mechanistic rationale of the featured transformation is depicted in Scheme 3. The reaction begins with two nucleophilic attacks by both hydrazine and hydrazide groups of the substrate at two of the phosphorylated nitronate species **A**. After subsequent elimination of the two molecules of *ortho*-phosphoric acid, the resulting species **4** underwent a double-fold 5-*endo-trig* nucleophilic cyclization employing both *N*-(1-hydrazineylalkylidene)-*O*-phosphonohydroxylammonium moieties. Next, tautomeric form **5** would undergo re-protonation to produce heterocyclic intermediate **6**. The latter experiences an elimination of two equivalents of hydroxylamine *O*-phosphate to afford the aromatic final product **3** (Scheme 3).

**Scheme 4:**
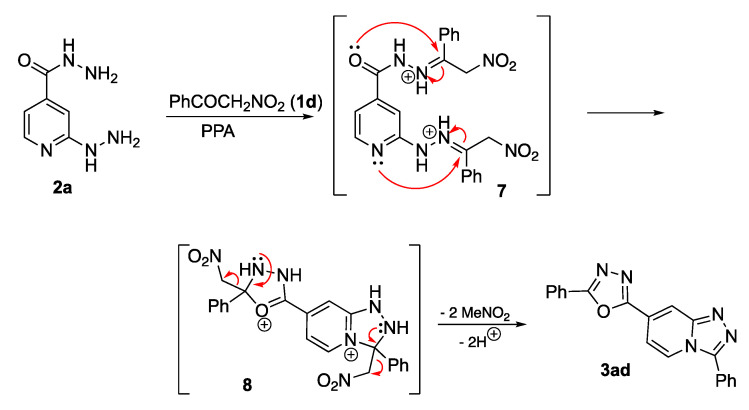
Mechanistic rationale involving electrophilic nitroacetophenones.

It should be pointed out that reactions involving α-nitrotoluene as a pro-electrophile proceeded sluggishly affording the corresponding bis-phenylsubstituted products (such as **3ad**) with disappointingly marginal yields (10% or below). This limitation, however, can be addressed by substituting α-nitrotoluene with α-nitroacetophenones (**1d** or **1f**, 3.0 equiv.). This tactical trick was first presented in our original report on the preparation of benzimidazoles and benzoxazoles in 2015 [20]. An updated version of this mechanistic rationale adapted for the reaction with bifunctional substrate **2a** is shown in Scheme 4. It is assumed that the initial acid-assisted double-fold interaction of **2a** with α-nitroacetophenone (**1d**) leads to the formation of bis-1-(2-nitroethylidene)hydrazin-1-ium as an intermediate **7** (Scheme 3). The electron pairs for the heteroatoms of ketone and pyridine form an intramolecular attack to afford two new five-membered rings as an intermediate **8**. The latter experiences a double-fold nucleofugal cleavage of nitromethane to produce molecule **3ad** (Scheme 4). Compounds **3cd**, **3dd**, and **3cf** were formed in a similar manner with high yields (Scheme 2).

In both discussed mechanistic rationales (Scheme 3 and Scheme 4), the initial nucleophilic attack of the hydrazine groups and subsequent annulations are occurring at two different sites. This would most likely take place independently with different kinetic rates, but for the sake of a concise depiction they are shown here to proceed in parallel.

**Scheme 5:**
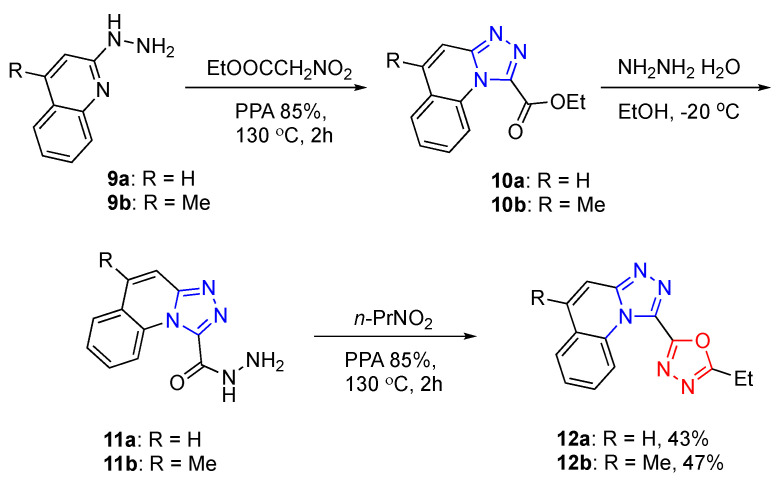
Stepwise installation of[1,2,4]triazolo[4,3-*a*]quinolines and 1,3,4-oxadiazole moieties.

We also decided to take advantage of the reactivity of the electrophilically activated ethyl nitroacetate (**1e**) to design a sequential approach for [1,2,4]triazolo[4,3-*a*]quinolines bearing 1,3,4-oxadiazole substituent at C-3 (shown in Scheme 5). To this end, 2-hydrazinylquinolines **9** were first utilized in the featured annulation to obtain triazoles **10** bearing an ester function at C-3. These compounds were taken without purification and subjected to the hydrazinolysis reaction to provide heterocyclic products **11**. The latter crude acylhydrazide moieties were transformed into 1,3,4-oxadiazole rings this time employing 1-nitropropane (**1a**) as an electrophilic component. Two compounds, **12a** and **12b**, were successfully synthesized via this approach with moderate yields (43% and 47%, respectively) (Scheme 5). In principle, the same strategy can potentially be employed for stepwise assembly of longer linear oligomeric chains with repeating 1,3,4-oxadiazole units, provided that greater annulation efficiency could be achieved at every step.

**Figure 1:**
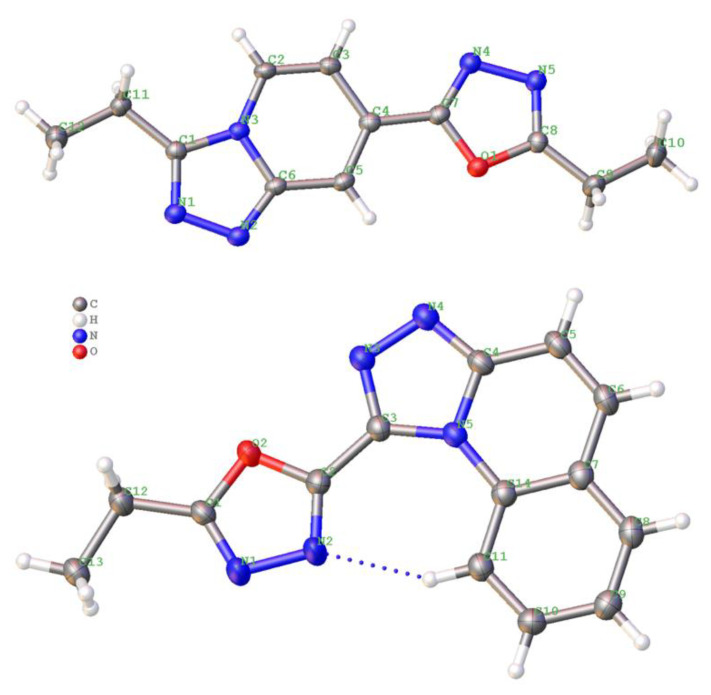
ORTEP drawing of X-ray structures of (2-ethyl-5-(3-ethyl-[1,2,4]triazolo[4,3-*a*]pyridin-6-yl)-1,3,4-oxadiazole) (**3ba**, CCDC #2092596) and 1-(5-ethyl-1,3,4-oxadiazol-2-yl)[1,2,4]triazolo[4,3-*a*]quinoline (**12a**, CCDC #2093198). The thermal ellipsoids are shown at 50% probability.

Formation of triazole and oxadiazole cycles in both parallel and sequential modes of the featured annulation was unambiguously confirmed by single-crystal X-ray diffraction of compounds **3ba** and **12a**, respectively (Figure 1).

## 3. Materials and Methods

### General

NMR spectra, ^1^H and ^13^C, were measured in solutions of CDCl_3_ or DMSO-*d*_6_ on Bruker AVANCE-III HD instrument (at 400.40 or 100.61 MHz, respectively. Bruker, Billerica, MA, USA). Residual solvent signals were used as internal standards, in DMSO*-d*_6_ (2.50 ppm for ^1^H, and 40.45 ppm for ^13^C nuclei) or in CDCl_3_ (7.26 ppm for ^1^H, and 77.16 ppm for ^13^C nuclei). HRMS spectra were measured on a Bruker maXis impact (electrospray ionization, in MeCN solutions, employing HCO_2_Na–HCO_2_H for calibration). IR spectra were measured on an FT-IR spectrometer Shimadzu IRAffinity-1S equipped with an ATR sampling module. Reaction progress, purity of isolated compounds, and R*_f_* values were monitored with TLC on Silufol UV-254 plates. Column chromatography was performed on silica gel (32–63 μm, 60 Å pore size). Melting points were measured with Stuart SMP30 apparatus. Polyphosphoric acid samples were prepared by dissolving precisely measured amounts of P_2_O_5_ in 85% *ortho*-phosphoric acid. Ethyl 2-chloroquinoline-4-carboxylate, ethyl 6-bromo-2-chloroquinoline-4-carboxylate [25], ethyl 2-chloroisonicotinate [26], and ethyl 6-chloronicotinate [27] were synthesized according to the published methods. All other reagents and solvents were purchased from commercial venders and used as received.

**2-Hydrazinylpyridine-4-carbohydrazide** (**2a**). Ethyl 2-chloroisonicotinate (740 mg, 4.00 mmol), hydrazine hydrate (88% solution in water, 2.3 mL, 40.0 mmol), and ethanol (0.7 mL) were combined in a 30 mL G30 vial and covered with a septum. The vial was placed in a Monowave 300 microwave reactor, and the mixture was heated to 160 °C over the course of 5 min (power did not exceed 135 watts), after which this temperature was maintained for 1.5 h (controlled by IR sensor, MW power within 10 watts, 10–15 bar pressure). The resulting mixture was poured into water (40 mL) and filtrated. The resulting precipitate was washed with water several times (2 × 30 mL). It was recrystallized from ethanol to afford **2a** as a pale-yellow solid, m.p. 171–172 °C (ethanol); yield 601 mg (3.60 mmol, 90%). R*_f_* 0.16, EtOAc/Et_3_N (20:0.1: *v*/*v*). ^1^H-NMR (400 MHz, DMSO-*d_6_*) δ 9.83 (br.s, NH, 1H), 8.03 (dd, *J* = 5.3, 0.8 Hz, 1H), 7.61 (br.s, NH, 1H), 7.07 (t, *J* = 1.1 Hz, 1H), 6.84 (dd, *J* = 5.2, 1.5 Hz, 1H), 4.51 (br.s, NH, 2H), 4.15 (br.s, NH, 2H). ^13^C-NMR (101 MHz, DMSO) δ 164.9, 162.5, 147.9, 141.5, 109.4, 104.3. FTIR (ZnSe) ν (cm^−1^): 3232, 2925, 2858, 1739, 1652, 1619, 1556, 1462, 1250, 1103, 1055, 874. HRMS (ES TOF, *m*/*z*) calculated for C_6_H_9_N_5_NaO^+^ ([M + Na]^+^): 190.0694, found: 190.0699 (2.9 ppm).

**2-Hydrazinylquinoline-4-carbohydrazide** (**2c**). Ethyl 2-chloroquinoline-4-carboxylate (940 mg, 4.00 mmol), hydrazine hydrate (88% solution in water, 2.3 mL, 40.0 mmol), and ethanol (0.7 mL) were combined and refluxed for 1 h. The resulting mixture was poured into water (40 mL) and filtrated. The resulting precipitate was washed with water several times (2 × 30 mL). It was recrystallized from ethanol to afford **2c** as a brown solid, m.p. 219–220 °C (decomp.) (ethanol); yield 781 mg (3.60 mmol, 90%). R*_f_* 0.43, EtOAc/Et_3_N (20:0.1: *v*/*v*). ^1^H-NMR (400 MHz, DMSO-*d*_6_) δ 9.78 (br.s, NH, 1H), 8.24 (s, 1H), 7.84–7.76 (m, 1H), 7.59–7.49 (m, 2H), 7.18 (d, *J* = 1.5 Hz, 1H), 6.83 (br.s, NH, 1H), 4.60 (br.s, NH, 2H), 4.36 (br.s, NH, 2H). ^13^C-NMR (101 MHz, DMSO) δ 166.4, 147.9, 141.7, 129.5, 125.81, 125.30, 121.7, 120.1, 109.5. FTIR (ZnSe) ν (cm^−1^): 3691, 3253, 3183, 1775, 1612, 1511, 1245, 972, 850. HRMS (ES TOF, *m*/*z*) calculated for C_10_H_11_N_5_NaO^+^ ([M + Na]^+^): 240.0848, found: 240.0856 (3.4 ppm).

**6-Bromo-2-hydrazinylquinoline-4-carbohydrazide** (**2d**). This material was obtained using the method described for preparation of compound **2a** employing ethyl 6-bromo-2-chloroquinoline-4-carboxylate (1252 mg, 4.00 mmol), and it was purified by recrystallization from ethanol to afford **2d** as a brown solid, m.p. = 215–216 °C (ethanol); yield 1065 g (3.60 mmol, 90%). R*_f_* = 0.56, EtOAc/Et_3_N (20:0.1: *v*/*v*). ^1^H-NMR (400 MHz, DMSO-*d*_6_) δ 9.86 (br.s, NH, 1H), 8.42 (s, 1H), 7.97 (d, *J* = 2.3 Hz, 1H), 7.62 (dd, *J* = 8.9, 2.4 Hz, 1H), 7.49 (d, *J* = 8.9 Hz, 1H), 6.89 (br.s, NH, 1H), 4.63 (br.s, NH, 2H), 4.40 (br.s, NH, 2H). ^13^C-NMR (101 MHz, DMSO) δ 165.9, 158.9, 146.9, 140.6, 132.3, 128.0, 127.3, 121.5, 113.7, 110.5. FTIR (ZnSe) ν (cm^−1^): 3325, 3205, 2935, 1665, 1633, 1520, 1380, 1245, 1043, 937, 756. HRMS (ES TOF, *m*/*z*) calculated for C_10_H_11_BrN_5_O^+^ ([M – H]^+^): 296.0138, found: 296.0141 (1.2 ppm). 

**[1,2,4]Triazolo[4,3-*a*]quinoline-1-carbohydrazide (11a).** Hydrazine hydrate (88% solution in water, 0.58 mL, 10.0 mmol) was added dropwise to a solution of the ethyl [1,2,4]triazolo[4,3-*a*]quinoline-1-carboxylate (241 mg, 1.00 mmol) in ethanol (5 mL), cooled to −10 °C. It was stirred at room temperature for 1 h, and then water (10 mL) was poured in. The precipitate was filtered off and washed with cold water (2 × 5 mL). This material can be used as is for the subsequent transformation. To obtain an analytical sample, the crude material was recrystallized from ethanol to afford the titled compound as a pale-yellow solid, m.p. = 205–206 °C (ethanol); yield 204 mg (90%). R*_f_* 0.60, EtOAc/Et_3_N (20:0.1: *v*/*v*). ^1^H-NMR (400 MHz, DMSO-*d*_6_) δ 10.12 (br.s, NH, 1H), 8.41 (d, *J* = 8.5 Hz, 1H), 8.07 (dd, *J* = 7.8, 1.6 Hz, 1H), 7.93 (d, *J* = 9.5 Hz, 1H), 7.85–7.71 (m, 2H), 7.66 (td, *J* = 7.6, 1.1 Hz, 1H), 4.97 (br.s, NH, 2H). ^13^C-NMR (101 MHz, DMSO) δ 158.5, 149.0, 143.3, 131.0, 130.5, 129.9, 129.5, 126.9, 124.0, 117.8, 114.1. FTIR (ZnSe) ν (cm^−1^): 3219, 2824, 1677, 1619, 1539, 1409, 1250, 1216, 1172, 1089, 944. HRMS (ES TOF, *m*/*z*) calculated for C_11_H_9_N_5_NaO^+^ ([M + Na]^+^): 250.0692, found: 250.0699 (2.9 ppm).

**5-Methyl-[1,2,4]triazolo[4,3-*a*]quinoline-1-carbohydrazide (11b).** This material was obtained by the method described for compound **2e** employing ethyl 5-methyl-[1,2,4]triazolo[4,3-*a*]quinoline-1-carboxylate (255 mg, 1.00 mmol). This material can be used as is for the subsequent transformation. To obtain an analytical sample, the crude material was recrystallized from ethanol to afford the titled compound as a pale-brown solid, m.p. = 214–215 °C (ethanol); yield 219 mg (91%). R*_f_* 0.26, EtOAc/Et_3_N (20:0.1: *v*/*v*). ^1^H-NMR (400 MHz, DMSO) δ 10.47 (bs, 1H), 8.43 (dd, *J* = 8.4, 1.2 Hz, 1H), 8.09 (dd, *J* = 8.1, 1.6 Hz, 1H), 7.77 (ddd, *J* = 8.6, 7.2, 1.6 Hz, 1H), 7.72–7.66 (m, 2H), 5.00 (bs, NH, 2H), 2.64 (s, 3H). ^13^C-NMR (101 MHz, DMSO) δ 158.6, 148.9, 143.0, 138.1, 130.3, 129.9, 126.8, 126.2, 124.3, 118.0, 112.9, 19.0. FTIR (ZnSe) ν (cm^−1^): 3296, 3209, 1674, 1522, 1414, 1375, 1303, 1248, 1168, 1091, 1036. HRMS (ES TOF, *m/z*) calculated for C_12_H_11_N_5_NaO^+^ ([M + Na]^+^): 264.0858, found: 264.0858 (−0.8 ppm).

**General method A (employing nitroalkanes)**. A 10 mL Erlenmeyer flask equipped with reflux condenser and magnetic stirrer was charged with 85% polyphosphoric acid (2.00 g), 2-hydrazinilisonicotinohydrazide **2** (1.00 equiv.), and nitroalkane **1** (4.00 equiv.). The mixture was placed in an oil bath that was preheated to 130 °C and stirred for 30 min. Then, another 2.00 equiv. of nitro compound **1** was added, followed by stirring for an additional 1.5 h. Then, the mixture was poured into cold H_2_O (5 mL), neutralized with aqueous ammonia to a pH 6–7, and extracted with EtOAc (4 × 5 mL). The combined extracts were concentrated under vacuum, and the residue was purified by preparative column chromatography on silica gel, eluting with a mixture of acetone and hexane.

**Method B with α-nitroacetophenone**. A 10 mL Erlenmeyer flask equipped with a magnetic stirrer and a reflux condenser was charged with 2-hydrazinilisonicotinohydrazide (1.00 equiv.) **2**, polyphosphoric acid (85% P_2_O_5_, 2 g), and α-nitroacetophenone **1d** (3.00 equiv.). The flask was placed in an oil bath and heated to 130 °C while being stirred. The mixture was heated for 1.5–2 h; when TLC analysis showed the reaction was completed, the reaction mixture was cooled. Water was added (5 mL), neutralized with 25% aqueous ammonia solution (4 mL) to pH = 8–9 and extracted with EtOAc (4 × 5 mL). The combined organic phases were concentrated and the crude product was purified by preparative column chromatography eluting with acetone and hexane.

**Method C with ethyl nitroacetate.** In a 10 mL Erlenmeyer flask equipped with a magnetic stirrer and a reflux condenser, 2-hydrazinilisonicotinohydrazide (1.00 equiv.) **2,** PPA 85% (1 g), H_3_PO_3_ (1 g), and ethyl nitroacetate **1e** (3.0 equiv.) were loaded. The flask was placed in an oil bath and heated to 130 °C while being stirred for 1 h. Then, another 2 equiv. of nitroacetic ether **1e** was added and heated for another hour; when TLC analysis showed the reaction was completed, the reaction mixture was cooled. Water was added (5 mL), before neutralizing with 25% aqueous ammonia solution (4 mL) to pH = 8–9 and extracting with EtOAc (4 × 5 mL). The combined organic phases were concentrated, and the crude product was purified by preparative column chromatography eluting with acetone and hexane.

**(2-Ethyl-5-(3-ethyl-[1,2,4]triazolo[4,3-*a*]pyridin-7-yl)-1,3,4-oxadiazole) (3aa):** This compound was obtained via Method A employing 2-hydrazinylisonicotinohydrazide (**2a**) (167 mg, 1.00 mmol) and 1-nitropropane (**1a**) (534 mg, 6.00 mmol), purifying by silica gel column chromatography (EtOAc/hexane, gradient 1:1–2:1, *v*/*v*). White powder, m.p. 230–232 °C (EtOAc), R*_f_* 0.63, acetone/hexane (2:1, *v*/*v*). Yield: 218 mg (0.90 mmol, 90%). ^1^H-NMR (400 MHz, CDCl_3_) δ 8.23 (q, *J* = 1.4 Hz, 1H), 8.01 (d, *J* = 7.2 Hz, 1H), 7.52 (dt, *J* = 7.2, 1.8 Hz, 1H), 3.11 (qd, *J* = 7.6, 1.4 Hz, 2H), 2.96 (qd, *J* = 7.6, 1.5 Hz, 2H), 1.50 (td, *J* = 7.6, 1.6 Hz, 3H), 1.42 (td, *J* = 7.6, 1.5 Hz, 3H). ^13^C-NMR (101 MHz, CDCl_3_) δ 168.9, 162.6, 149.2, 149.0, 122.8, 122.5, 115.0, 111.1, 19.3, 18.2, 10.9, 10.8. FTIR (ZnSe) *ν* (cm^−1^): 3740, 2997, 1561, 1525, 1433, 1371, 1192, 1021, 951, 867. HRMS (ES TOF, *m*/*z*) calculated for C_12_H_13_NaN_5_O^+^ ([M + Na]^+^): 266.1011, found: 266.1012 (0.4 ppm).

**(2-([1,2,4]Triazolo[4,3-*a*]pyridin-7-yl)-1,3,4-oxadiazole)** (**3ac**): This compound was obtained via Method A employing 2-hydrazinylisonicotinohydrazide (**2a**) (167 mg, 1.00 mmol) and 1-nitromethane (**1c**) (427 mg, 7.00 mmol), purifying by silica gel column chromatography (gradient acetone/hexane 1:1, *v*/*v*–acetone). Pale brown solid, m.p. > 300 °C (acetone), R*_f_* 0.40, acetone. Yield: 103 mg (0.55 mmol, 55%). ^1^H-NMR (400 MHz, DMSO-*d*_6_) δ 9.35 (s, 1H), 8.66 (dt, *J* = 7.2, 1.3 Hz, 1H), 7.94 (s, 1H), 7.55 (s, 1H), 7.38 (dt, *J* = 7.3, 1.6 Hz, 1H). ^13^C-NMR (101 MHz, DMSO) δ 164.5, 155.5, 148.1, 137.3, 126.2, 123.5, 110.4, 110.1. FTIR (ZnSe) *ν* (cm^−1^): 3614, 3085, 1743, 1689, 1532, 1505, 1469, 1414, 1245, 1166, 1101, 1040, 970. HRMS (ES TOF, *m*/*z*) calculated for C_8_H_5_N_5_NaO^+^ ([M + Na]^+^): 210.0392, found: 210.0386 (−2.8 ppm).

**(2-Phenyl-5-(3-phenyl-[1,2,4]triazolo[4,3-*a*]pyridin-7-yl)-1,3,4-oxadiazole) (3ad):** This compound was obtained via Method B employing 2-hydrazinylisonicotinohydrazide (**2a**) (167 mg, 1.00 mmol) and α-nitroacetophenone (**1d**) (495 mg, 3.00 mmol), purifying by silica gel column chromatography (benzene/Et_3_N, 20:1, *v*/*v*). Light-green solid, m.p. 225–226 °C (EtOAc), R*_f_* 0.63, acetone/hexane (1:1, *v*/*v*). Yield: 298 mg, 88%. ^1^H-NMR (400 MHz, CDCl_3_) δ 9.04 (dd, *J* = 7.5, 0.8 Hz, 1H), 8.49 (dd, *J* = 1.8, 0.8 Hz, 1H), 8.48–8.42 (m, 2H), 8.23–8.19 (m, 2H), 7.93 (dd, *J* = 7.6, 1.8 Hz, 1H), 7.65–7.59 (m, 3H), 7.55–7.52 (m, 3H). ^13^C-NMR (101 MHz, CDCl_3_) δ 165.8, 162.4, 149.9, 147.2, 132.6, 131.0, 129.7 (2C), 129.5 (2C), 128.5 (2C), 127.4 (2C), 125.9, 123.7, 123.3, 123.2, 115.3, 112.2. FTIR (ZnSe) *ν* (cm^−1^): 3315, 3070, 1672, 1657, 1604, 1578, 1549, 1455, 1383, 1281, 1180, 1077, 1028, 930. HRMS (ES TOF, *m*/*z*) calculated for C_20_H_13_NaN_5_O^+^ ([M + Na]^+^): 362.1003, found: 362.1012 (2.6 ppm).

**(2-Ethyl-5-(3-ethyl-[1,2,4]triazolo[4,3-*a*]pyridin-6-yl)-1,3,4-oxadiazole) (3ba):** This compound was obtained via Method A employing 6-hydrazinylnicotinohydrazide (**2b**) (167 mg, 1.00 mmol) and 1-nitropropane (**1a**) (534 mg, 6.00 mmol), purifying by silica gel column chromatography (gradient acetone/hexane, 1:1, *v*/*v*–acetone). Pale-brown solid, m.p. 116–117 °C (EtOAc), R*_f_* 0.23, acetone/hexane (1:1, *v*/*v*). Yield: 298 mg (0.88 mmol, 88%). ^1^H-NMR (400 MHz, CDCl_3_) δ 8.60 (d, *J* = 1.5 Hz, 1H), 7.82 (dd, *J* = 4.1, 1.3 Hz, 2H), 3.15 (q, *J* = 7.5 Hz, 2H), 2.96 (q, *J* = 7.6 Hz, 2H), 1.53 (t, *J* = 7.5 Hz, 3H), 1.43 (t, *J* = 7.6 Hz, 3H). ^13^C-NMR (101 MHz, CDCl_3_) δ 172.0, 168.2, 161.7, 149.1, 124.4, 121.7, 117.5, 112.4, 19.3, 18.3, 10.91, 10.85. FTIR (ZnSe) *ν* (cm^−1^): 3325, 3205, 2935, 1665, 1633, 1520, 138, 1245, 1043, 937. HRMS (ES TOF, *m*/*z*) calculated for C_12_H_13_NaN_5_O^+^ ([M + Na]^+^): 266.1004, found: 266.1014 (2.9 ppm).

**(1-Ethyl-5-(5-ethyl-1,3,4-oxadiazol-2-yl)[1,2,4]triazolo[4,3-*a*]quinoline) (3ca):** This compound was obtained via Method A employing 2-hydrazinylquinoline-4-carbohydrazide (**2c**) (217 mg, 1.00 mmol) and 1-nitropropane (**1a**) (534 mg, 6.00 mmol), purifying by silica gel column chromatography (acetone/hexane, gradient 1:2–1:1–2:1, *v*/*v*). Colorless powder, m.p. 235–236 °C (acetone), R*_f_* 0.50, acetone/hexane (1:1, *v*/*v*). Yield: 254 mg (0.87 mmol, 87%). ^1^H-NMR (400 MHz, CDCl_3_) δ 9.47 (dd, *J* = 8.3, 1.6 Hz, 1H), 8.35 (s, 1H), 8.28 (dd, *J* = 8.6, 1.2 Hz, 1H), 7.79 (ddd, *J* = 8.6, 7.2, 1.5 Hz, 1H), 7.68 (ddd, *J* = 8.4, 7.2, 1.2 Hz, 1H), 3.55 (q, *J* = 7.4 Hz, 2H), 3.05 (q, *J* = 7.6 Hz, 2H), 1.69 (t, *J* = 7.3 Hz, 3H), 1.50 (t, *J* = 7.6 Hz, 3H). ^13^C-NMR (101 MHz, CDCl_3_) δ 168.2, 162.5, 152.0, 148.9, 132.6, 130.3, 129.3, 126.8, 122.0, 121.7, 117.6, 116.4, 23.5, 19.3, 11.5, 10.9. FTIR (ZnSe) *ν* (cm^−1^): 2930, 2862, 1737, 1657, 1561, 1440, 1380, 1240, 1180, 1055, 997, 879. HRMS (ES TOF, *m/z*) calculated for C_16_H_15_NaN_5_O^+^ ([M + Na]^+^): 316.1166, found: 316.1169 (1.0 ppm).

**(2-Heptyl-5-(1-heptyl-[1,2,4]triazolo[4,3-*a*]quinolin-5-yl)-1,3,4-oxadiazole) (3cb):** This compound was obtained via Method A employing 2-hydrazinylquinoline-4-carbohydrazide (**2c**) (217 mg, 1.00 mmol) and 1-nitrooctane (**1b**) (636 mg, 4.00 mmol), purifying by silica gel column chromatography (EtOAc/hexane, gradient 1:3–1:2–1:1, *v*/*v*). Dark brown solid, m.p. 198–200 °C (EtOAc), R*_f_* 0.73, acetone/hexane (2:1, *v*/*v*). Yield: 355 mg (0.82 mmol, 82%). ^1^H-NMR (400 MHz, CDCl_3_-*d*) δ 9.48 (d, *J* = 8.1 Hz, 1H), 8.33 (s, 1H), 8.26 (d, *J* = 8.4 Hz, 1H), 7.79 (t, *J* = 7.7 Hz, 1H), 7.68 (t, *J* = 7.6 Hz, 1H), 3.50 (t, *J* = 7.6 Hz, 2H), 3.01 (t, *J* = 7.5 Hz, 2H), 2.08 (quint, *J* = 7.7 Hz, 2H), 1.91 (quint, *J* = 7.4 Hz, 2H), 1.62–1.54 (m, 3H), 1.32 (ddt, *J* = 10.4, 7.4, 3.6 Hz, 13H), 0.89 (t, *J* = 6.7 Hz, 6H). ^13^C-NMR (101 MHz, CDCl_3_) δ 167.5, 162.5, 151.1, 148.9, 132.7, 130.3, 129.3, 126.8, 121.9, 120.8, 117.7, 116.4, 31.8, 31.7, 29.8, 29.5, 29.2, 29.1, 28.9, 26.8, 26.7, 25.53, 22.8, 22.7, 14.22, 14.19. FTIR (ZnSe) *ν* (cm^−1^): 3026, 2935, 2848, 1869, 1727, 1566, 1469, 1399, 1243, 1166, 997, 946. HRMS (ES TOF, *m/z*) calcd for C_26_H_35_NaN_5_O^+^ ([M + Na]^+^): 456.2728, found: 456.2734 (1.4 ppm).

**(2-Phenyl-5-(1-phenyl-[1,2,4]triazolo[4,3-*a*]quinolin-5-yl)-1,3,4-oxadiazole) (3cd):** This compound was obtained via Method B employing 2-hydrazinylquinoline-4-carbohydrazide (**2c**) (217 mg, 1.00 mmol) and α-nitroacetophenone (**1d**) (495 mg, 3.00 mmol), purifying by silica gel column chromatography (EtOAc/hexane, gradient 1:1– 3:1, *v*/*v*). Pale-brown solid, m.p. 243–245 °C (acetone), R*_f_* 0.53, EtOAc/hexane (3:1, *v*/*v*). Yield: 350 mg (0.90 mmol, 90%). ^1^H-NMR (400 MHz, CDCl_3_) δ 9.57 (dd, *J* = 8.3, 1.5 Hz, 1H), 8.68 (s, 1H), 8.28–8.21 (m, 2H), 7.75 (dd, *J* = 8.1, 1.5 Hz, 3H), 7.72–7.58 (m, 7H), 7.54–7.49 (m, 1H). ^13^C-NMR (101 MHz, CDCl_3_) δ 165.0, 162.2, 149.9, 148.3, 137.0, 132.7, 132.0, 131.3, 130.3, 130.11 (2C), 129.52 (4C), 129.4, 128.7, 127.52 (3C), 123.3, 120.9, 117.3, 116.8. FTIR (ZnSe) *ν* (cm^−1^): 3335, 3195, 2988, 1783, 1761, 1655, 1554, 1375, 1243, 1050, 937. HRMS (ES TOF, *m/z*) calculated for C_24_H_15_NaN_5_O^+^ ([M + Na]^+^): 412.1177, found: 412.1169 (−2.1 ppm).

**2-(*p*-Tolyl)-5-(1-(*p*-tolyl)-[1,2,4]triazolo[4,3-*a*]quinolin-5-yl)-1,3,4-oxadiazole (3cf):** This compound was obtained via Method B employing 2-hydrazinylquinoline-4-carbohydrazide (**2c**) (217 mg, 1.00 mmol) and 1-(4-methylphenyl)-2-nitroethan-1-one (**1f**) [28] (394 mg, 2.2 mmol), purifying by silica gel column chromatography (ACETONE/hexane, gradient 1:3–1:2, *v*/*v*). Pale-brown solid, m.p. 190–191 °C (acetone), R*_f_* 0.53, acetone/hexane (1:1, *v*/*v*). Yield: 304 mg (0.73 mmol, 73%). ^1^H-NMR (400 MHz, CDCl_3_) δ 9.50 (d, *J* = 9.3 Hz, 1H), 8.56 (s, 1H), 8.10 (d, *J* = 8.2 Hz, 2H), 7.79–7.76 (m, 1H), 7.62 (d, *J* = 8.1 Hz, 3H), 7.50–7.46 (m, 1H), 7.42 (dd, *J* = 13.7, 7.9 Hz, 4H), 2.53 (s, 3H), 2.48 (s, 3H). ^13^C-NMR (101 MHz, CDCl_3_) δ 165.0, 162.1, 150.2, 148.8, 143.3, 141.3, 132.2, 130.2 (2C), 130.1 (2C), 129.90 (2C), 129.88, 129.2, 127.4 (2C), 127.1, 126.1, 122.4, 120.8, 120.5, 117.4, 117.2, 21.9, 21.8. FTIR (ZnSe) *ν* (cm^−1^): 2920, 2858, 1713, 1614, 1554,1513, 1465, 1380, 1250, 1180, 1016, 951. HRMS (ES TOF, *m*/*z*) calculated for C_26_H_19_NaN_5_O^+^ ([M + Na]^+^): 440.1470, found: 440.1482 (−2.7 ppm).

**2-Methyl-5-(1-methyl-[1,2,4]triazolo[4,3-*a*]quinolin-5-yl)-1,3,4-oxadiazole (3cg):** This compound was obtained via Method B employing 2-hydrazinylquinoline-4-carbohydrazide (**2c**) (217 mg, 1.00 mmol) and nitroethane (**1g**) (225 mg, 3.00 mmol), purifying by silica gel column chromatography (acetone/hexane, gradient 1:1–acetone, *v*/*v*). White solid, m.p. 250–251 °C (acetone), R*_f_* 0.29, acetone. Yield: 219 mg (0.83 mmol, 83%). ^1^H-NMR (400 MHz, CDCl_3_) δ 9.46 (dd, *J* = 8.3, 1.5 Hz, 1H), 8.32 (dd, *J* = 8.5, 1.2 Hz, 1H), 8.29 (s, 1H), 7.78 (ddd, *J* = 8.6, 7.2, 1.5 Hz, 1H), 7.68 (ddd, *J* = 8.3, 7.2, 1.2 Hz, 1H), 3.21 (s, 3H), 2.72 (s, 3H). ^13^C-NMR (101 MHz, CDCl_3_) δ 164.1, 162.7, 148.8, 147.3, 132.7, 130.4, 129.3, 126.9, 121.9, 120.6, 117.5, 116.1, 16.4, 11.3. FTIR (ZnSe) *ν* (cm^−1^): 2925, 2853, 1703, 1652, 1614, 1578, 1508, 1472, 1395, 12540, 1180, 1161, 1055, 939. HRMS (ES TOF, *m*/*z*) calculated for C_14_H_11_NaN_5_O^+^ ([M + Na]^+^): 288.0849, found: 288.0856 (2.5 ppm).

**2-(4-Methylbenzyl)-5-(1-(4-methylbenzyl)-[1,2,4]triazolo[4,3-*a*]quinolin-5-yl)-1,3,4-oxadiazole (3ch):** This compound was obtained via Method B employing 2-hydrazinylquinoline-4-carbohydrazide (**2c**) (217 mg, 1.00 mmol) and 1-methyl-4-(2-nitroethyl)benzene (**1h**) [29] (330 mg, 2.00 mmol), purifying by silica gel column chromatography (acetone/hexane, gradient 1:3–1:2, *v*/*v*). White solid, m.p. 214–215 °C (acetone), R*_f_* 0.49, acetone/hexane (1:2, *v*/*v*). Yield: 187 mg (0.42 mmol, 42%). ^1^H-NMR (400 MHz, CDCl_3_) δ 9.38 (dd, *J* = 6.4, 3.5 Hz, 1H), 8.30 (s, 1H), 8.10 (dd, *J* = 6.4, 3.4 Hz, 1H), 7.58 (dd, *J* = 6.4, 3.4 Hz, 2H), 7.31–7.28 (m, 2H), 7.20 (d, *J* = 7.8 Hz, 2H), 7.13–7.06 (m, 4H), 4.90 (s, 2H), 4.32 (s, 2H), 2.36 (s, 3H), 2.29 (s, 3H). ^13^C-NMR (101 MHz, CDCl_3_) δ 165.9, 162.9, 149.2, 149.1, 137.8, 137.3, 132.0, 131.7, 130.4, 130.3, 130.0 (3C), 130.0, 129.0 (3C), 128.1 (2C), 126.9, 122.1, 120.5, 117.7, 117.1, 34.5, 31.6, 21.3, 21.2. FTIR (ZnSe) ν (cm^−1^): 3672, 2930, 2858, 1744, 1718, 1684, 1652, 1558, 1508, 1457, 1428, 1243, 1168, 1055, 992. HRMS (ES TOF, *m*/*z*) calculated for C_28_H_23_NaN_5_O^+^ ([M + Na]^+^): 468.1802, found: 468.1795 (−1.6 ppm).

**((2-(7-Bromo-1-ethyl-[1,2,4]triazolo[4,3-*a*]quinolin-5-yl)-5-ethyl-1,3,4-oxadiazole) (3da):** This compound was obtained via Method A employing 6-bromo-2-hydrazinylquinoline-4-carbohydrazide (**2d**) (295 mg, 1.00 mmol) and 1-nitropropane (**1a**) (534 mg, 6.00 mmol), purifying by silica gel column chromatography (gradient acetone/hexane 1:1, *v*/*v*–acetone). Light-brown solid, m.p. 320–322 °C (acetone), R*_f_* 0.66, acetone. Yield: 218 mg (0.59 mmol, 59%). ^1^H-NMR (400 MHz, DMSO-*d*_6_) δ 10.65 (d, *J* = 1.3 Hz, 1H), 10.09 (d, *J* = 1.3 Hz, 1H), 8.47 (d, *J* = 2.3 Hz, 1H), 8.34 (d, *J* = 9.2 Hz, 1H), 7.98 (dd, *J* = 9.1, 2.4 Hz, 1H), 7.83 (s, 1H), 3.49 (q, *J* = 7.3 Hz, 2H), 2.26 (q, *J* = 7.6 Hz, 2H), 1.50 (t, *J* = 7.3 Hz, 3H), 1.11 (t, *J* = 7.6 Hz, 3H). ^13^C-NMR (101 MHz, DMSO-*d*_6_) δ 173.0, 165.5, 152.1, 148.2, 133.1, 132.7, 131.6, 129.7, 123.6, 119.9, 119.1, 116.0, 27.0, 22.7, 11.5, 10.1. FTIR (ZnSe) *ν* (cm^−1^): 3301, 3205, 2992, 2906, 2848, 1775, 1669, 1534, 1457, 1375, 1243, 1166, 1048, 932. HRMS (ES TOF, *m*/*z*) calculated for C_16_H_14_BrNaN_5_O^+^ ([M + Na]^+^): 394.0270, found: 394.0274 (0.9 ppm).

**(2-(7-Bromo-[1,2,4]triazolo[4,3-*a*]quinolin-5-yl)-1,3,4-oxadiazole) (3dc):** This compound was obtained via Method A employing 6-bromo-2-hydrazinylquinoline-4-carbohydrazide (**2d**) (295 mg, 1.00 mmol) and 1-nitromethane (**1c**) (427 mg, 7.00 mmol), purifying by silica gel column chromatography (gradient acetone/hexane, 2:1, *v*/*v*–acetone). Colorless solid, m.p. 311–312 °C (acetone), R*_f_* 0.67, acetone. Yield: 195 mg, 62%. ^1^H-NMR (400 MHz, DMSO-*d*_6_) δ 10.17 (d, *J* = 0.8 Hz, 1H), 9.59 (s, 1H), 9.36 (d, *J* = 2.2 Hz, 1H), 8.57 (d, *J* = 8.9 Hz, 1H), 8.51 (d, *J* = 0.8 Hz, 1H), 8.15 (dd, *J* = 8.9, 2.2 Hz, 1H). ^13^C-NMR (101 MHz, DMSO) δ 161.5, 154.8, 146.1, 137.6, 133.4 (2C), 129.9, 129.8, 120.8, 120.4, 119.5, 118.3. FTIR (ZnSe) *ν* (cm^−1^): 3629, 3099, 1746, 1705, 1684, 1532, 1505, 1469, 1414, 1245, 1166, 1101, 1040, 970. HRMS (ES TOF, *m*/*z*) calculated for C_12_H_6_BrN_5_NaO^+^ ([M + Na]^+^): 337.9652, found: 337.964 (−1.3 ppm).

**(2-(7-Bromo-1-phenyl-[1,2,4]triazolo[4,3-*a*]quinolin-5-yl)-5-phenyl-1,3,4-oxadiazole) (3dd):** This compound was obtained via Method B employing 6-bromo-2-hydrazinylquinoline-4-carbohydrazide (**2d**) (295 mg, 1.00 mmol) and α-nitroacetophenone (**1d**) (495 mg, 3.00 mmol), purifying by silica gel column chromatography (gradient acetone/hexane, 1:1, *v*/*v*–acetone). Brown solid, m.p. 247–248 °C (acetone), R*_f_* 0.51 (acetone/hexane, 1:1, *v*/*v*). Yield: 378 mg (0.68 mmol, 68%). ^1^H-NMR (400 MHz, chloroform-*d*) δ 9.78 (t, *J* = 1.3 Hz, 1H), 8.64 (s, 1H), 8.24–8.21 (m, 2H), 7.74–7.62 (m, 8H), 7.59 (d, *J* = 1.3 Hz, 2H). ^13^C-NMR (101 MHz, DMSO) δ 133.3, 132.8, 131.85, 131.4, 130.0 (5C), 129.7 (3C), 129.6 (5C), 127.5 (4C), 118.6, 117.91, 117.90. FTIR (ZnSe) *ν* (cm^−1^): 3613, 3080, 1747, 1675, 1601, 1568, 1557, 1457, 1373, 1282, 1114, 1077, 945. HRMS (ES TOF, *m*/*z*) calculated for C_24_H_14_BrN_5_NaO^+^ ([M + Na]^+^): 490.0258, found: 490.0274 (3.3 ppm).

**(Ethyl 5-(3-(ethoxycarbonyl)-[1,2,4]triazolo[4,3-*a*]pyridin-7-yl)-1,3,4-oxadiazole-2-carboxylate) (3ae):** This compound was obtained via Method C employing 2-hydrazinylisonicotinohydrazide (**2a**) (167 mg, 1.00 mmol) and ethyl 2-nitroacetate (**1e**) (400 mg, 3.00 mmol), purifying by silica gel column chromatography (acetone/hexane 1:4, *v*/*v*). Colorless power, m.p. 199–200 °C (acetone), R*_f_* 0.37, acetone/hexane (1:1, *v*/*v*). Yield: 215 mg, 65%. ^1^H-NMR (400 MHz, CDCl_3_) δ 9.33 (dd, *J* = 7.3, 1.1 Hz, 1H), 8.80–8.62 (m, 1H), 7.88 (dd, *J* = 7.3, 1.6 Hz, 1H), 4.61 (dq, *J* = 11.1, 7.1 Hz, 4H), 1.52 (dt, *J* = 10.6, 7.1 Hz, 6H). ^13^C-NMR (101 MHz, CDCl_3_) δ 163.6, 158.1, 157.3, 153.9, 150.5, 138.6, 127.0, 123.5, 116.4, 113.3, 64.1, 63.0, 14.3, 14.1. FTIR (ZnSe) *ν* (cm^−1^): 2925, 2848, 1739, 1698, 1563, 1532, 1462, 1387, 1313, 1255, 1187, 157, 1011, 934, 848. HRMS (ES TOF, *m*/*z*) calculated for C_14_H_13_N_5_NaO_5_^+^ ([M + Na]^+^): 354.0797, found: 354.0809 (3.4 ppm).

**Ethyl [1,2,4]triazolo[4,3-*a*]quinoline-1-carboxylate (10a).** This compound was obtained via Method C (only PPA) employing 2-hydrazinylquinoline (159 mg, 1.00 mmol) and ethyl 2-nitroacetate (**2e**) (400 mg, 3.00 mmol). This material can be used in the subsequent transformation in crude form. The sample for analytical purposes was purified by silica gel column chromatography (acetone/hexane 1:2, *v*/*v*) to obtain a pale-yellow solid, m.p. 145–146 °C (acetone); yield: 140 mg, 58%. R*_f_* 0.33, acetone/hexane (1:1, *v*/*v*). ^1^H-NMR (400 MHz, CDCl_3_) δ 8.85 (dq, *J* = 8.7, 0.8 Hz, 1H), 7.84 (dd, *J* = 7.8, 1.6 Hz, 1H), 7.77–7.68 (m, 3H), 7.59 (ddd, *J* = 8.2, 7.3, 1.1 Hz, 1H), 4.64 (q, *J* = 7.1 Hz, 2H), 1.55 (t, *J* = 7.2 Hz, 3H). ^13^C-NMR (101 MHz, CDCl_3_) δ 159.9, 151.2, 142.0, 132.0, 131.6, 129.9, 129.4, 127.3, 124.9, 119.4, 114.6, 63.4, 14.3. FTIR (ZnSe) *ν* (cm^−1^): 3325, 3243, 3002, 1761, 1677, 1527, 1443, 1375, 1248, 1091, 1055, 949. HRMS (ES TOF, *m*/*z*) calculated for C_13_H_11_N_3_NaO_2_^+^ ([M + Na]^+^): 264.0739, found: 264.0743 (1.9 ppm).

**1-(5-Ethyl-1,3,4-oxadiazol-2-yl)[1,2,4]triazolo[4,3-*a*]quinoline (12a).** This compound was obtained via Method A employing [1,2,4]triazolo[4,3-*a*]quinoline-1-carbohydrazide (**11a**) (227 mg, 1.00 mmol) and 1-nitropropane (**1a**) (267 mg, 3.00 mmol), purifying by silica gel column chromatography (acetone/hexane 1:2–1:1, *v*/*v*). Pale-brown solid, m.p. 160–161 °C (acetone); yield: 140 mg, 43%. R*_f_* 0.37, acetone/hexane (1:1, *v*/*v*). ^1^H-NMR (400 MHz, CDCl_3_) δ 9.56 (dq, *J* = 8.6, 0.8 Hz, 1H), 7.88 (dd, *J* = 7.9, 1.6 Hz, 1H), 7.82–7.72 (m, 3H), 7.63 (ddd, *J* = 8.2, 7.3, 1.1 Hz, 1H), 3.11 (q, *J* = 7.6 Hz, 2H), 1.54 (t, *J* = 7.6 Hz, 3H). ^13^C-NMR (101 MHz, CDCl_3_) δ 169.7, 157.2, 151.4, 136.8, 132.0, 131.8, 130.3, 129.4, 127.6, 124.9, 119.3, 114.6, 19.5, 10.9. FTIR (ZnSe) *ν* (cm^−1^): 3320, 3234, 2992, 1763, 1679, 1554, 1378, 1250, 1052, 937. HRMS (ES TOF, *m*/*z*) calculated for C_14_H_11_N_5_NaO^+^ ([M + Na]^+^): 288.0847, found: 288.0856 (3.1 ppm).

**2-Ethyl-5-(5-methyl-[1,2,4]triazolo[4,3-*a*]quinolin-1-yl)-1,3,4-oxadiazole (12b).** This compound was obtained via Method A employing 5-methyl-[1,2,4]triazolo[4,3-*a*]quinoline-1-carbohydrazide (**11b**) (241 mg, 1.00 mmol) and 1-nitropropane (**1a**) (267 mg, 3.00 mmol), purifying by silica gel column chromatography (acetone/hexane 1:2–1:1, *v*/*v*). Pale-yellow solid, m.p. 147–149 °C (acetone); yield: 127 mg, 47%. R*_f_* 0.40, acetone/hexane (1:1, *v*/*v*). ^1^H-NMR (400 MHz, CDCl_3_) δ 9.59 (d, *J* = 8.5 Hz, 1H), 8.00 (dd, *J* = 8.0, 1.5 Hz, 1H), 7.75 (ddd, *J* = 8.7, 7.3, 1.5 Hz, 1H), 7.70–7.62 (m, 2H), 3.10 (q, *J* = 7.6 Hz, 2H), 2.71 (s, 3H), 1.53 (t, *J* = 7.6 Hz, 3H). ^13^C-NMR (101 MHz, CDCl_3_) δ 169.3, 157.9, 150.9, 139.3, 136.8, 132.0, 130.0, 127.5, 125.9, 125.4, 119.4, 113.7, 19.95, 19.4, 10.9. FTIR (ZnSe) *ν* (cm^−1^): 2930, 2858, 1746, 1648, 1566, 1460, 1385, 1243, 1163, 1115, 1062, 966, 848, 807. HRMS (ES TOF, *m*/*z*) calculated for C_15_H_13_N_5_NaO^+^ ([M + Na]^+^): 302.1003, found: 302.1012 (3.1 ppm).

## 4. Conclusions

In conclusion, an efficient protocol for simultaneous parallel cyclization of [1,2,4]triazolo[4,3-*a*]quinoline and 1,3,4-oxadiazole heterocyclic rings was developed. The featured transformation involved an unprecedented double-fold nucleophilic attack by two different hydrazine moieties of 2-hydrazineylpyridinecarbohydrazide substrates on nitroalkanes electrophilically activated in a polyphosphoric acid medium. The method provides an expeditious and direct access to [1,2,4]triazolo[4,3-*a*]quinolines bearing a 1,3,4-oxadiazole substituent, which might be of interest for medicinal chemistry. Furthermore, this research paves the road for development of other acid-mediated cascade transformations for preparation of complex heterocyclic compounds. Synthesis of a focused library for biological studies is currently underway in our laboratories.

## Figures and Tables

**Table 1:**
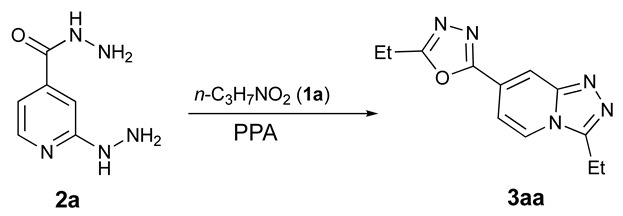
Optimization of the reaction conditions for a one-pot double-annulation toward a chimeric product **3aa**.

	Medium ^a^	T(°C)	PrNO_2_, eq.	Yield % ^b^
1	PPA, 80%	130	4 + 2	71
2	PPA, 87%	120	4 + 2	80
3	PPA, 87%	130	4 + 2	84
4	PPA, 85%	110	4 + 2	52
5	PPA, 85%	120	4 + 2	79
**6**	**PPA, 85%**	**130**	**4 + 2**	**90 ^c^**
7	PPA, 85%	140	4 + 2	79

^a^ P_2_O_5_ content is shown in %; ^b^ NMR yields are reported unless specified otherwise; ^c^ isolated yield of purified product is provided.

## Data Availability

Supplementary Materials include NMR and HRMS spectral charts and X-ray crystallography data.

## Supplementary Materials

Supporting Information data include NMR and HRMS spectral charts and X-ray crystallography data.
