# Supplementary material for: Electrophilically Activated Nitroalkanes in Double Annulation of [1,2,4]Triazolo[4,3-a]quinolines and 1,3,4-Oxadiazole Rings
Source: Molecules. 2021 Sep 20;26(18):5692. doi: 10.3390/molecules26185692 (PMC8464891; doi:10.3390/molecules26185692)
Supplement: Supplementary file 1 [file molecules-26-05692-s001.zip › molecules-1357868-supplementary.pdf]

Electronic Supplementary Material (ESI)

Electrophilically activated nitroalkanes in double annulation of [1,2,4]triazolo[4,3-a]quinolines and 1,3,4-oxadiazole rings

Alexander V. Aksenov,[a*] Nikita K. Kirilov,[a] Nicolai A. Aksenov,a Dmitrii A. Aksenov,[a] Elena A. Sorokina,[b]

Carolyn Lower,[c] and Michael Rubin,[*a,c]

a. Department of Chemistry, North Caucasus Federal University, 1a Pushkin St., Stavropol 355017, Russia. E-mail: aaksenov@ncfu.ru
b. Organic Chemistry Department, Peoples' Friendship, University of Russia (RUDN University), 6, Miklukho-Maklaya St., Moscow, 117198, Russian Federation
c. Department of Chemistry, University of Kansas, 1567 Irving Hill Road, Lawrence, KS 66045, USA. Tel: +1-785-864- 5071, E-mail: mrubin@ku.edu

Supporting Information

NMR Spectral Charts........ **S2**
^{1}H and ^{13}C NMR spectral charts for starting hydrazineylcarbohydrazides........ **S2**
^{1}H and ^{13}C NMR spectral charts for [1,2,4]triazolo[4,3-a]pyridin-(6)7-yl)-1,3,4-oxadiazoles........ **S12**
^{1}H and ^{13}C NMR spectral charts for 1,3,4-oxadiazol-2-yl)[1,2,4]triazolo[4,3-a]quinolines........ **S22**
HRMS charts........ **S46**
HRMS charts for starting hydrazineylcarbohydrazides........ **S46**
HRMS charts for for [1,2,4]triazolo[4,3-a]pyridin-(6)7-yl)-1,3,4-oxadiazoles........ **S48**
HRMS charts for 1,3,4-oxadiazol-2-yl)[1,2,4]triazolo[4,3-a]quinolines........ **S50**
X-Ray crystallography data........ **S56**
References........ **S72**

^{1}H and ^{13}C NMR spectral charts for starting hydrazineylcarbohydrazides

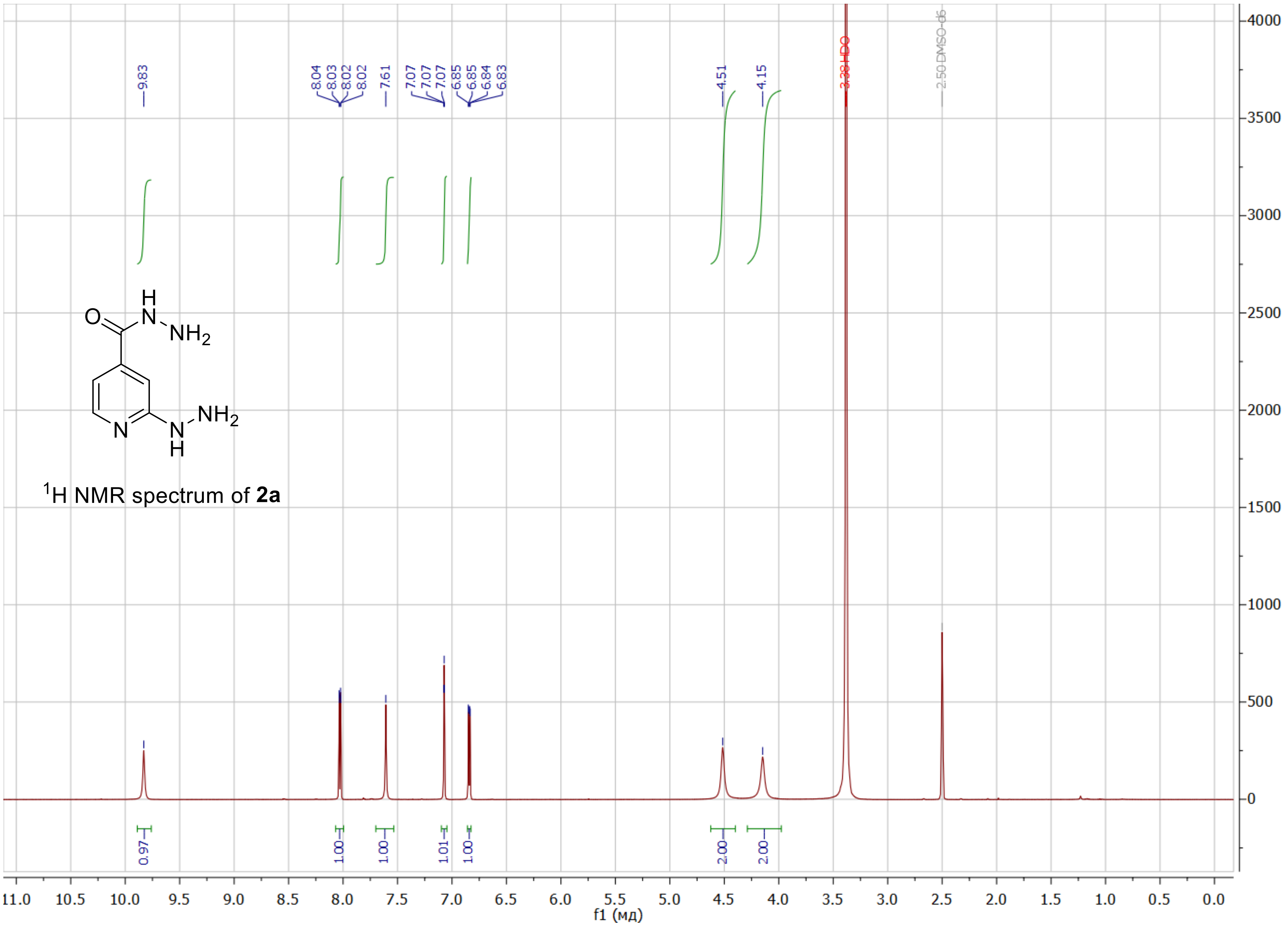

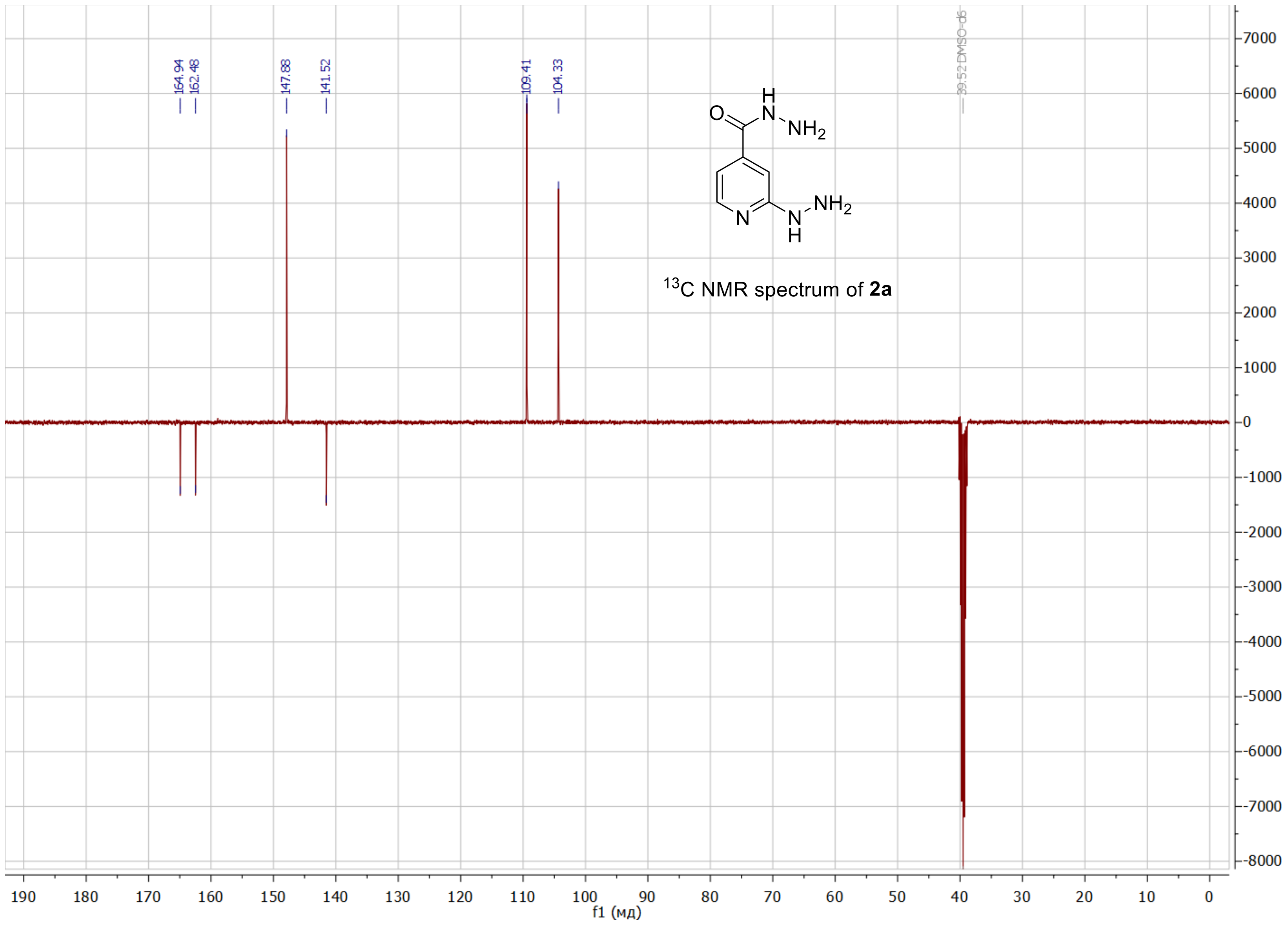
164.94
162.48
147.88
141.52
109.41
104.33
39.52 DMSO-d6
O
H
N
NH2
N
N
H
NH2
13C NMR spectrum of 2a
f1 (мд)

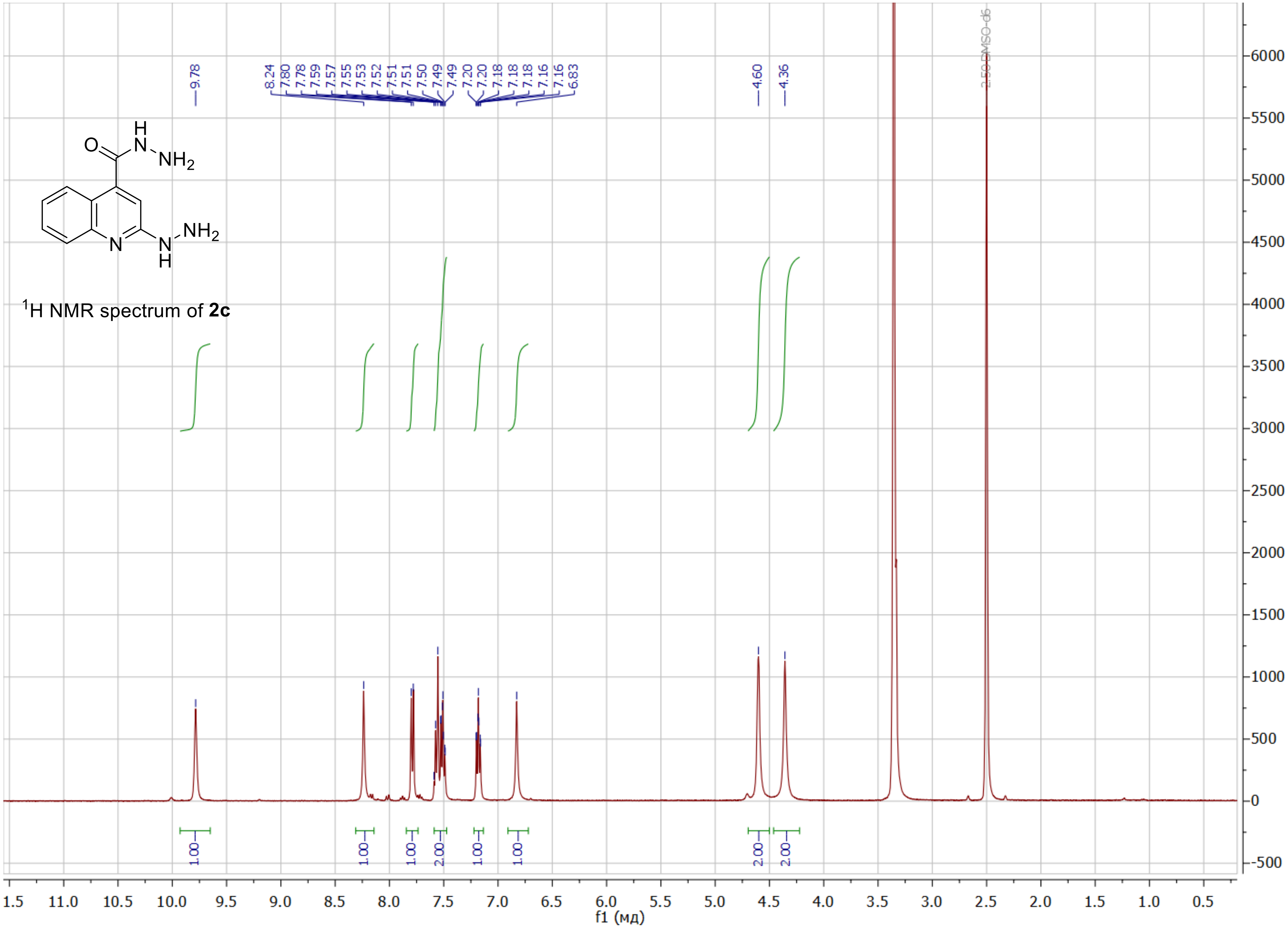

^{1}H NMR spectrum of **2c**
f1 (мд)
2.50 DMSO-d6

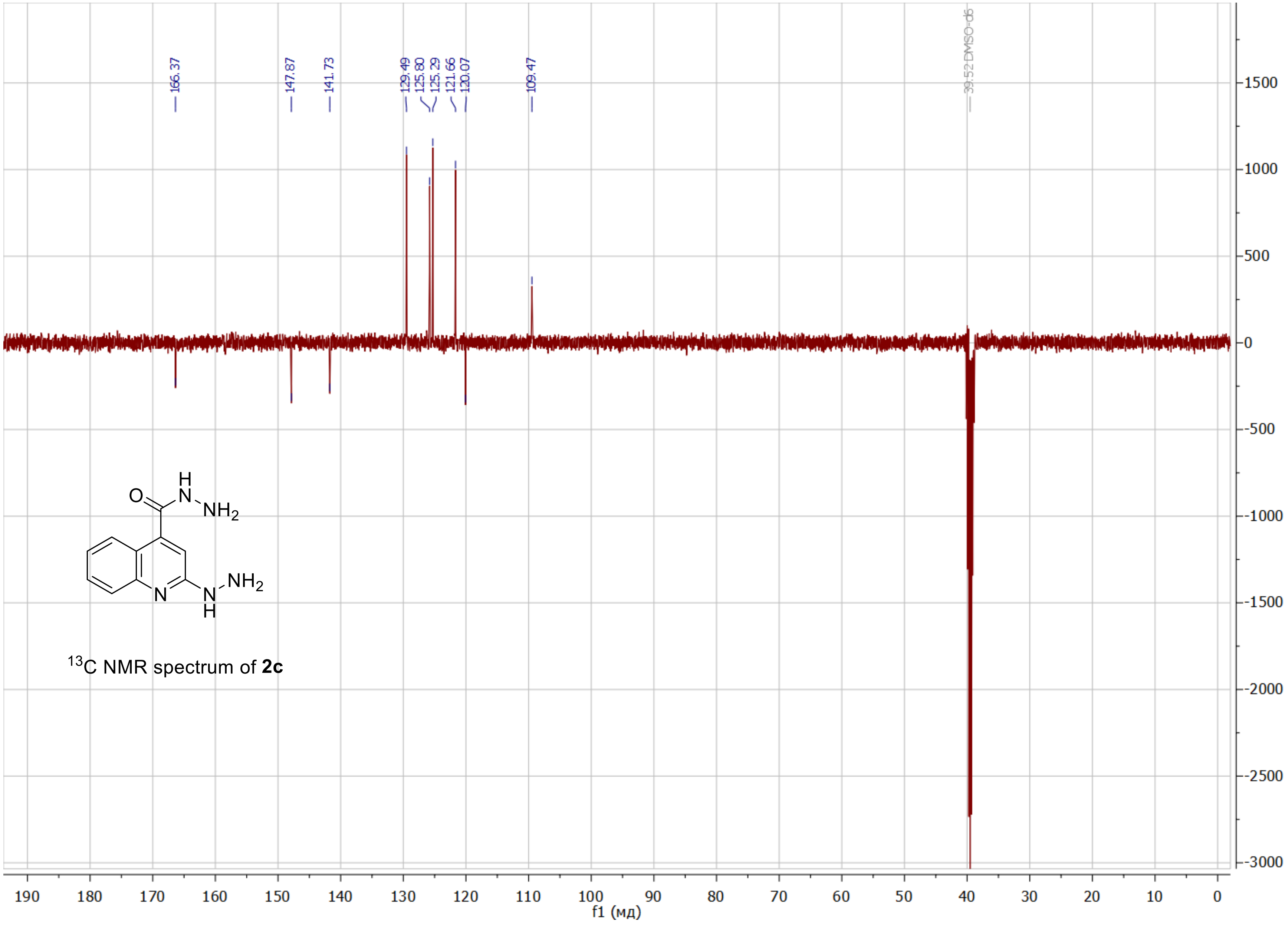

^{13}C NMR spectrum of **2c**

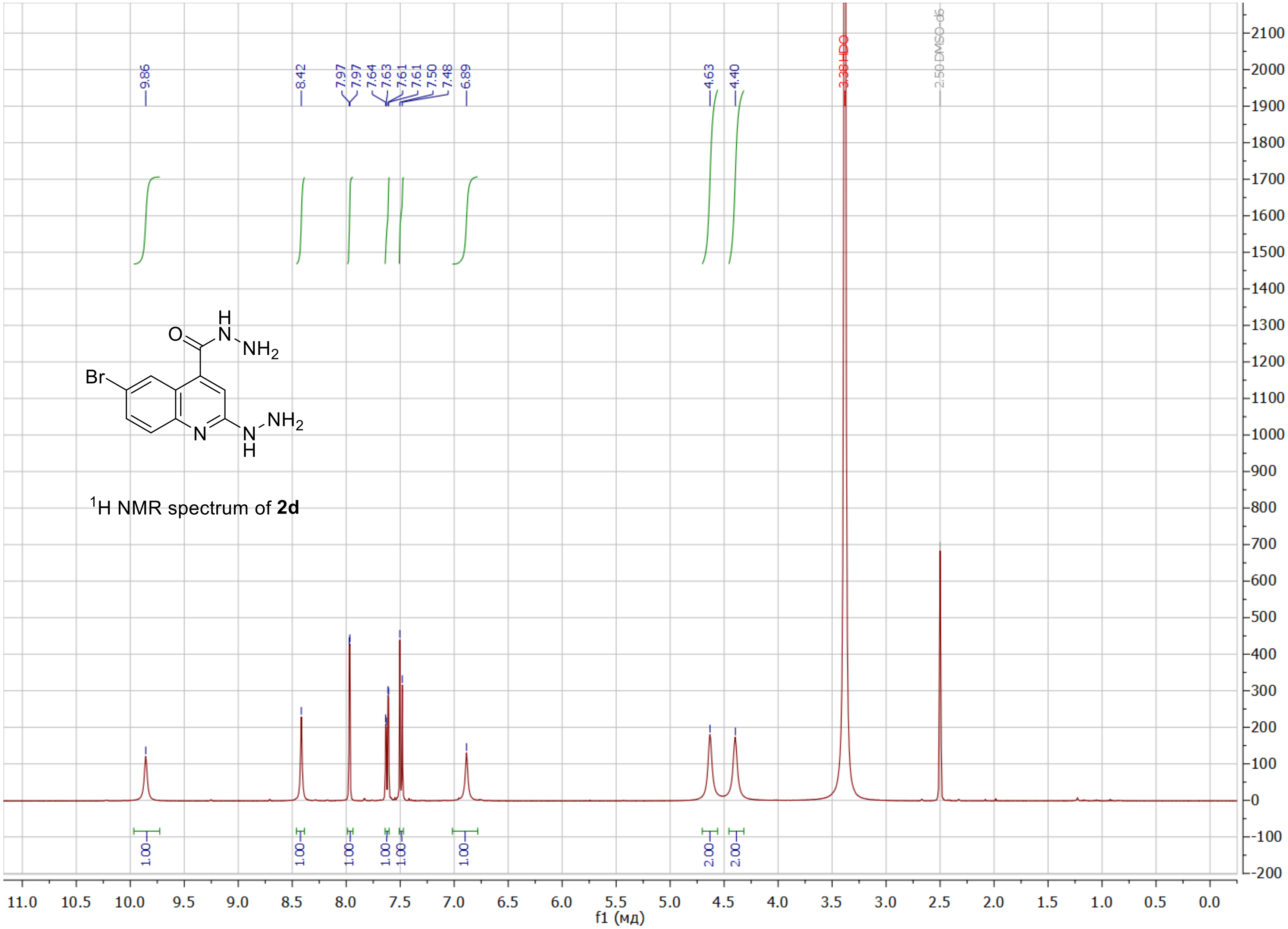

9.86
8.42
7.97
7.97
7.64
7.63
7.61
7.61
7.50
7.48
6.89
4.63
4.40
3.38 HDO
2.50 DMSO-d6
1.00
1.00
1.00
1.00
1.00
1.00
2.00
2.00
Br
O
H
N
NH2
N
N
H
NH2
^{1}H NMR spectrum of **2d**
f1 (мд)

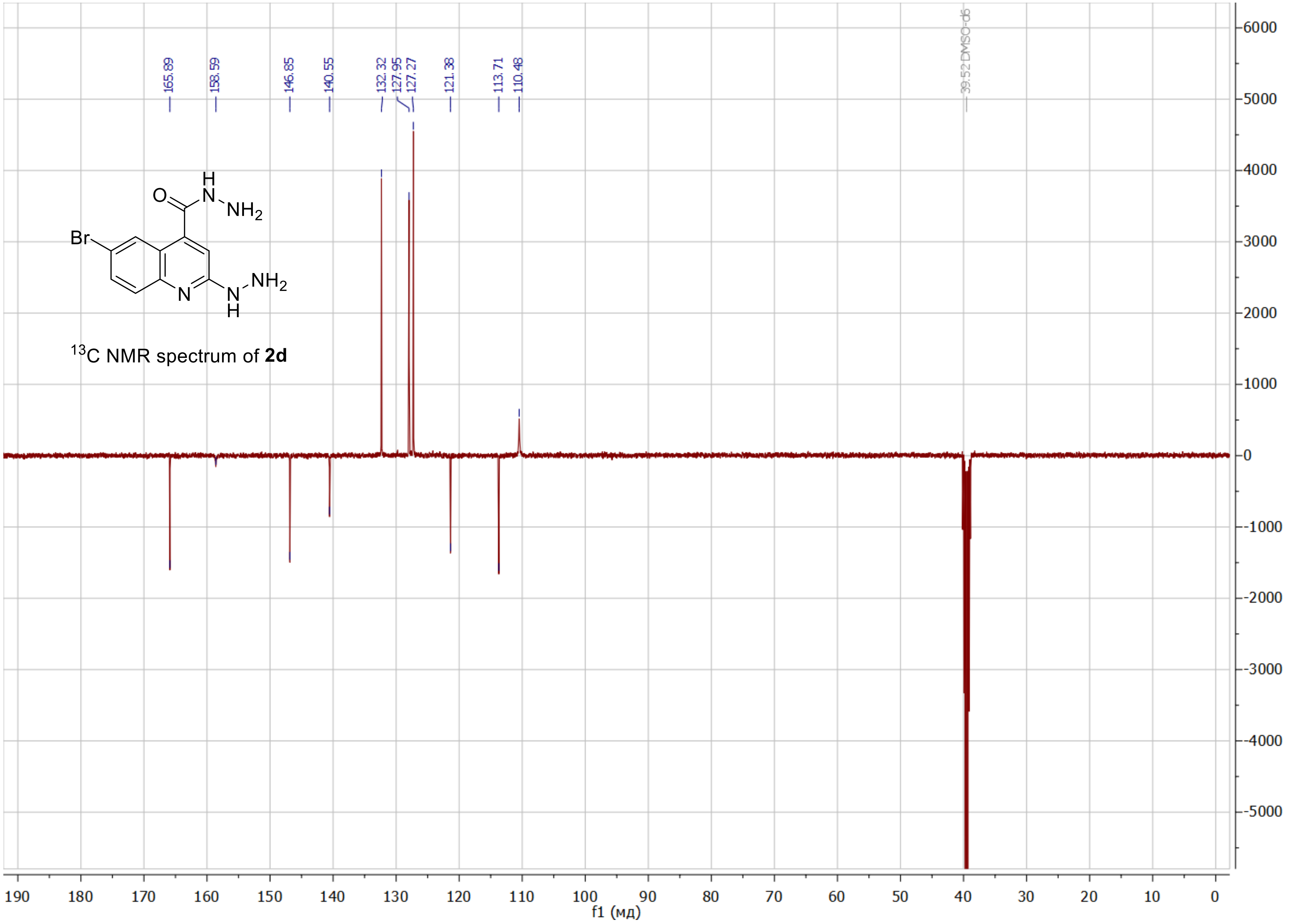
165.89
158.59
146.85
140.55
132.32
127.95
127.27
121.38
113.71
110.48
39.52 DMSO-d6
O
H
N
NH2
Br
N
N
H
NH2
13C NMR spectrum of 2d
6000
5000
4000
3000
2000
1000
0
-1000
-2000
-3000
-4000
-5000
190
180
170
160
150
140
130
120
110
100
90
80
70
60
50
40
30
20
10
0
f1 (мд)

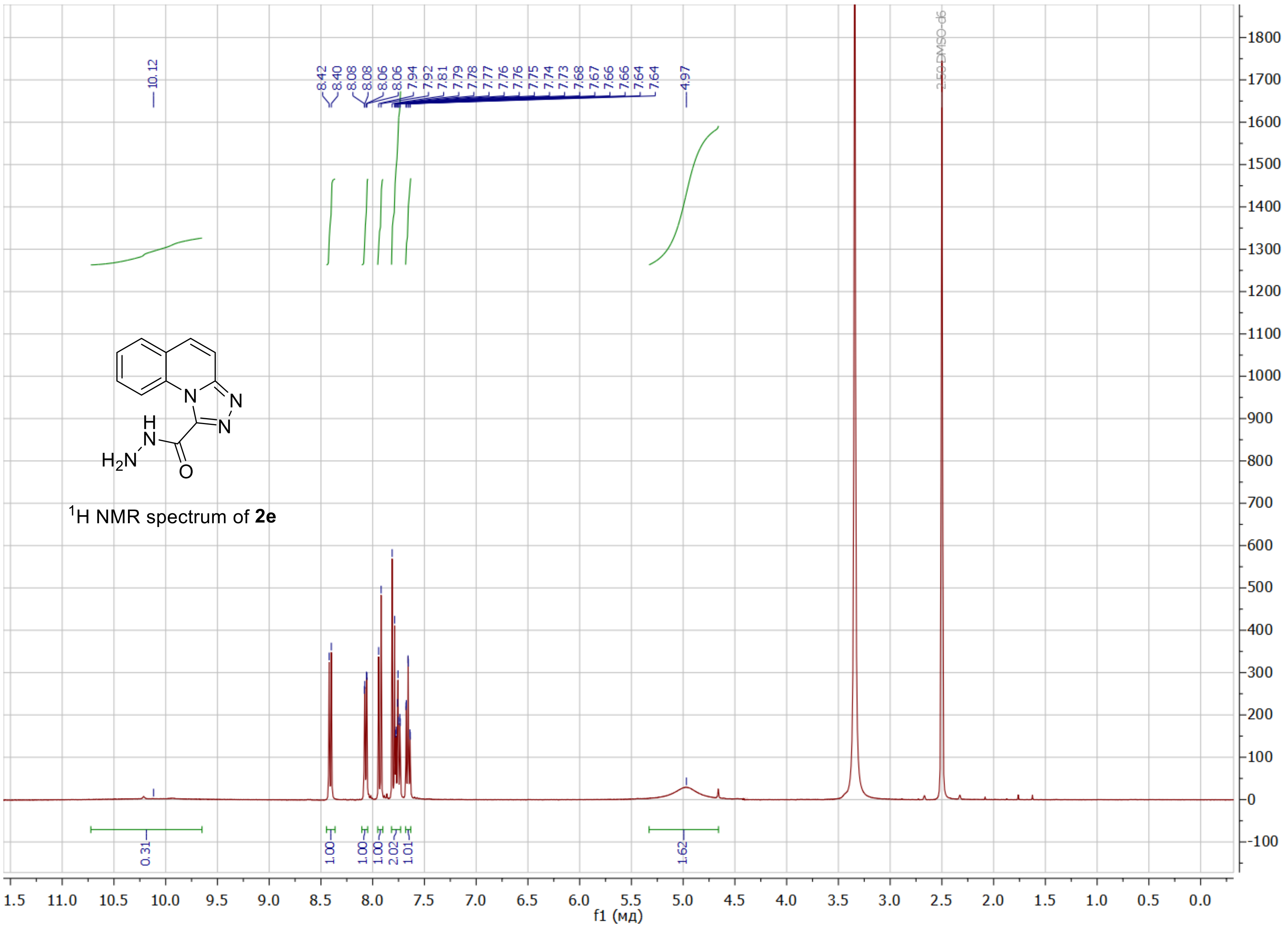

^{1}H NMR spectrum of **2e**

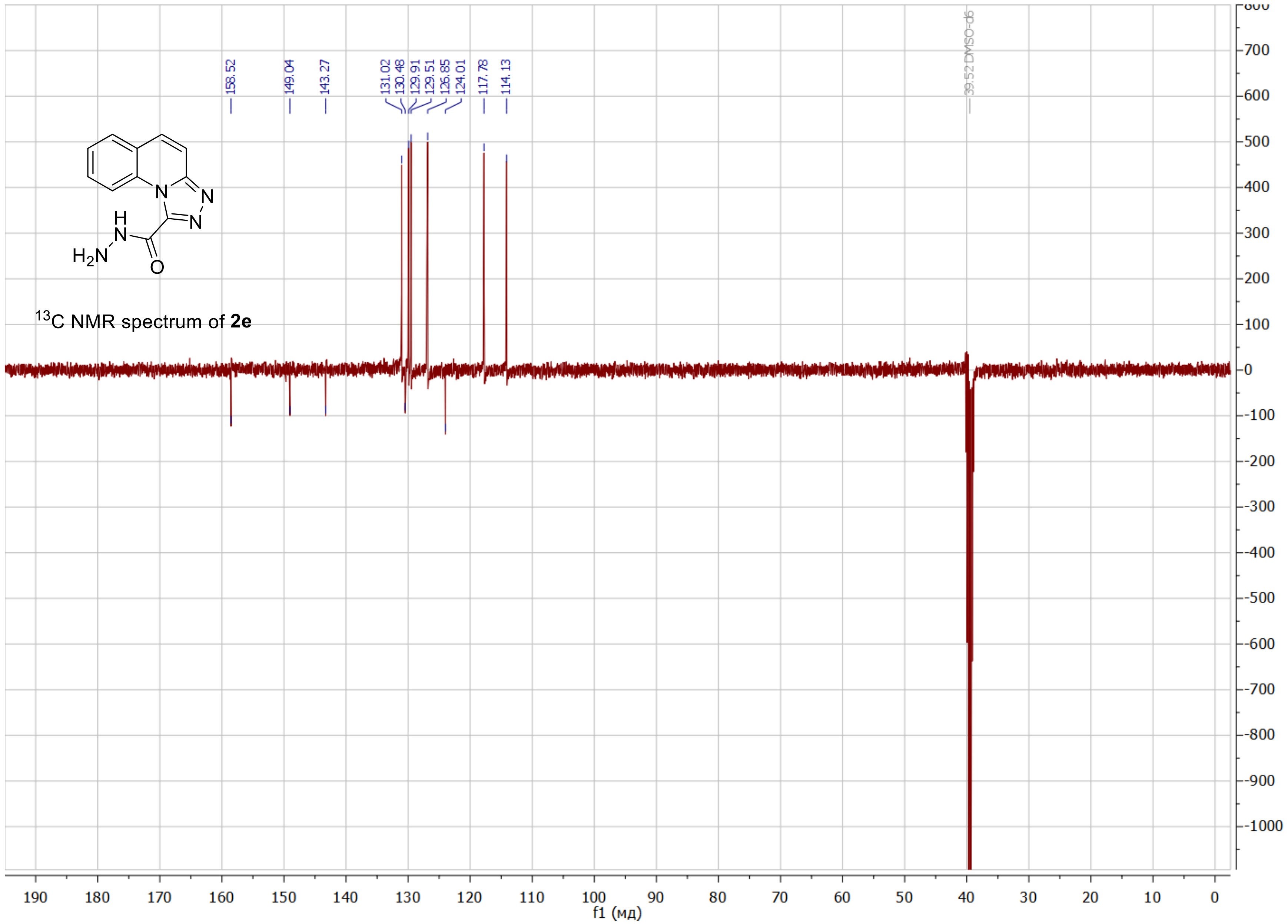

158.52
149.04
143.27
131.02
130.48
129.91
129.51
126.85
124.01
117.78
114.13
39.52 DMSO-d6
^{13}C NMR spectrum of **2e**
f1 (мд)

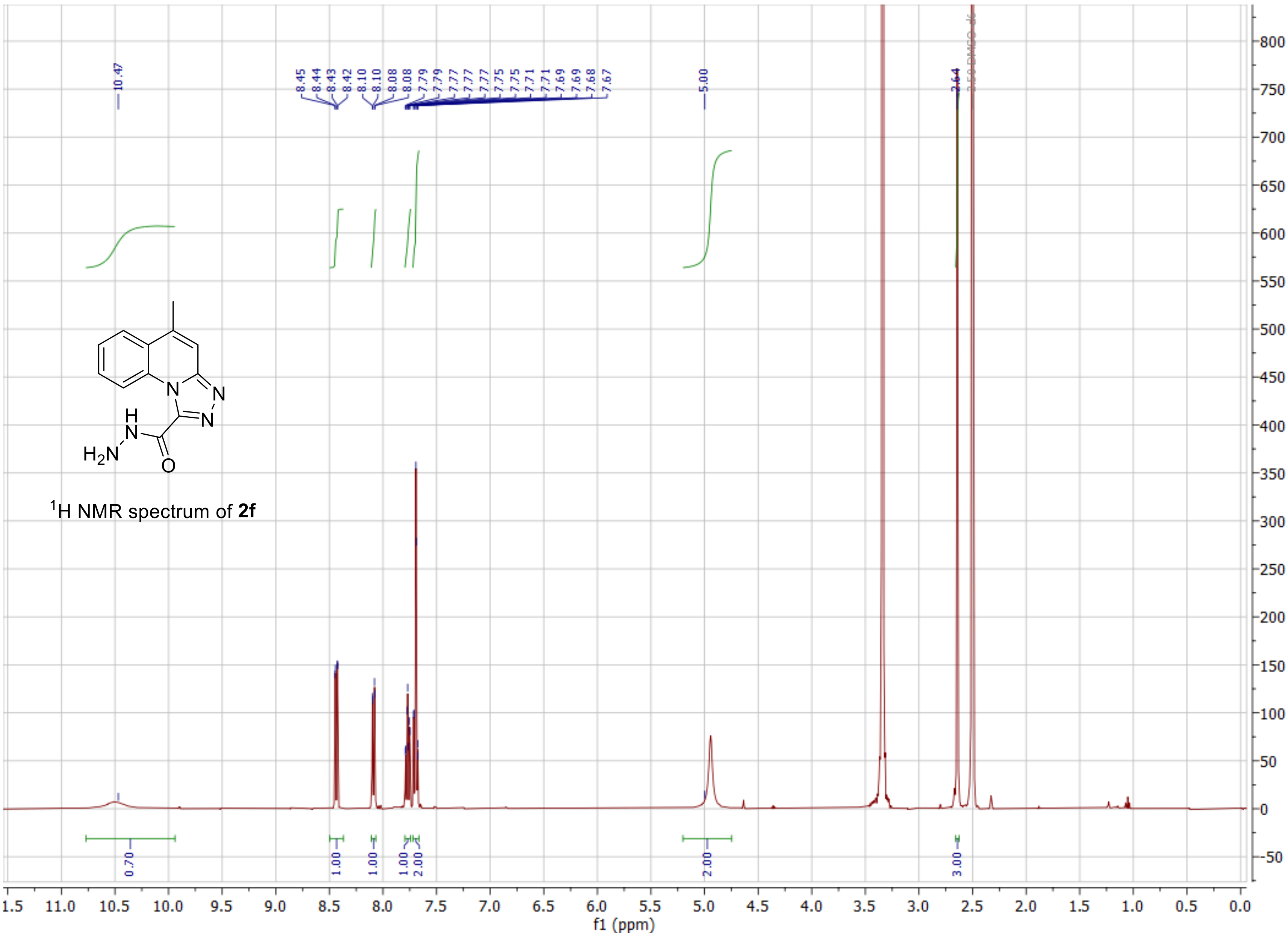

^{1}H NMR spectrum of **2f**

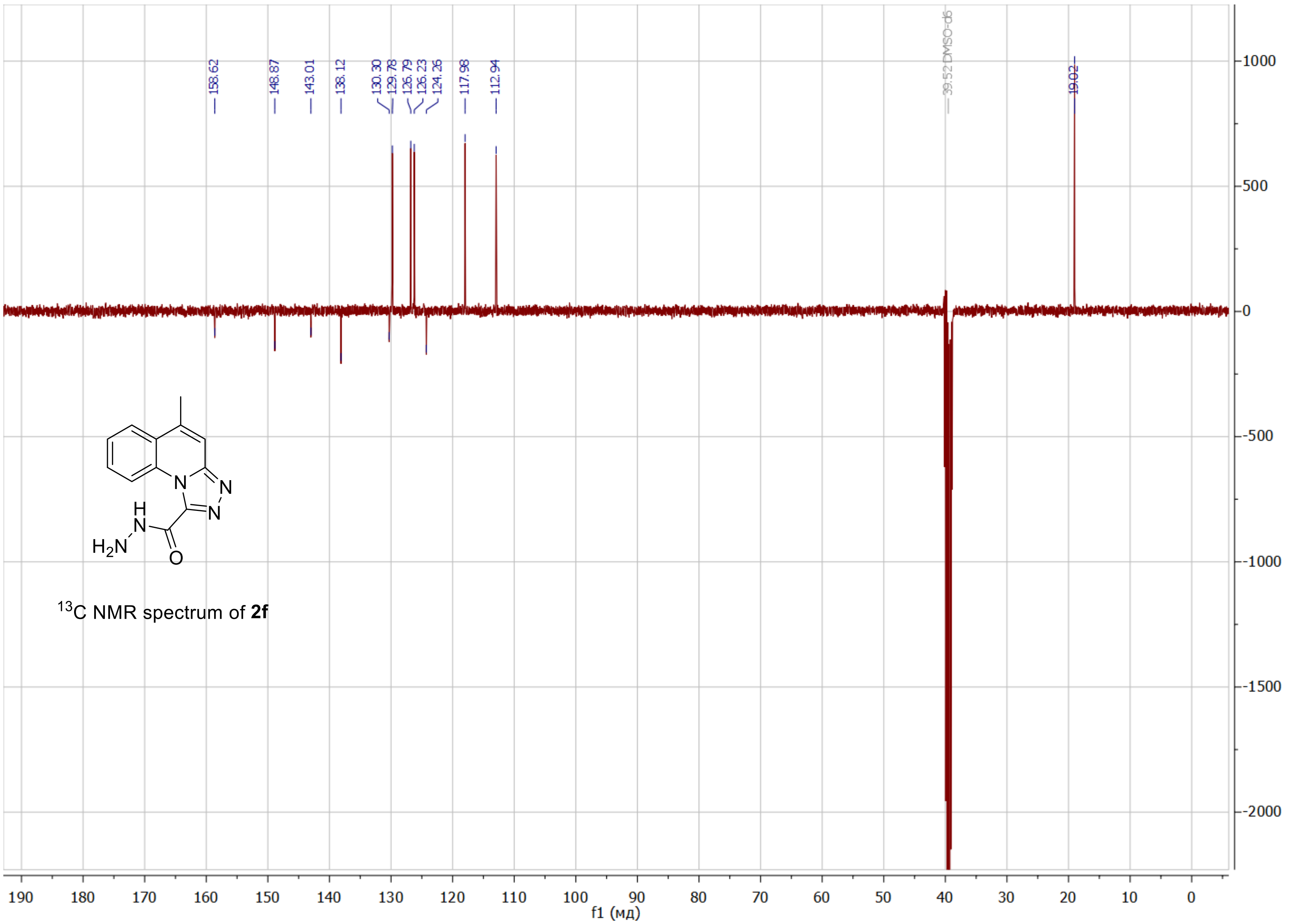

^{13}C NMR spectrum of **2f**

^{1}H and ^{13}C NMR spectral charts for ([1,2,4]triazolo[4,3-a]pyridin-(6)7-yl)-1,3,4-oxadiazoles

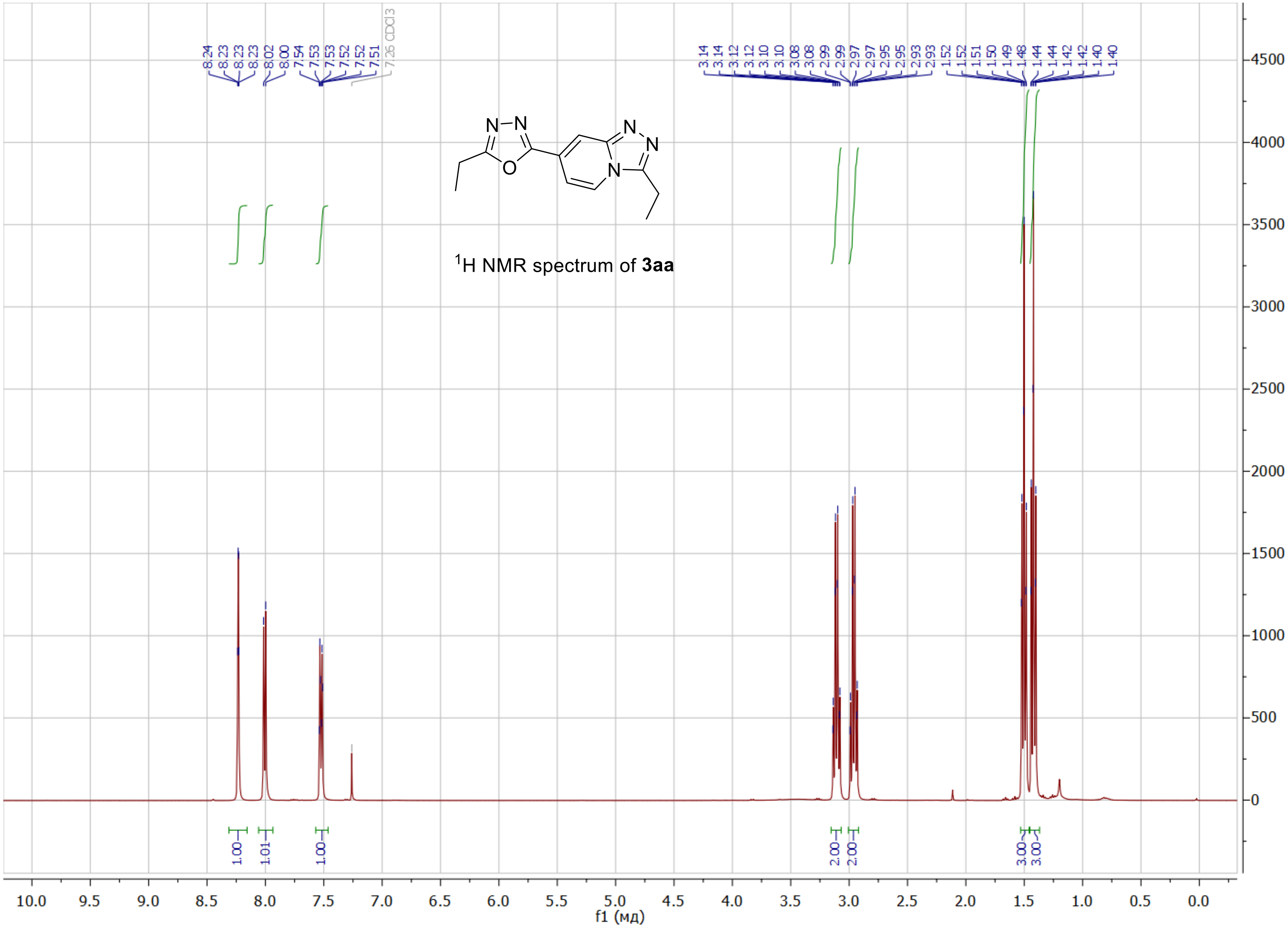

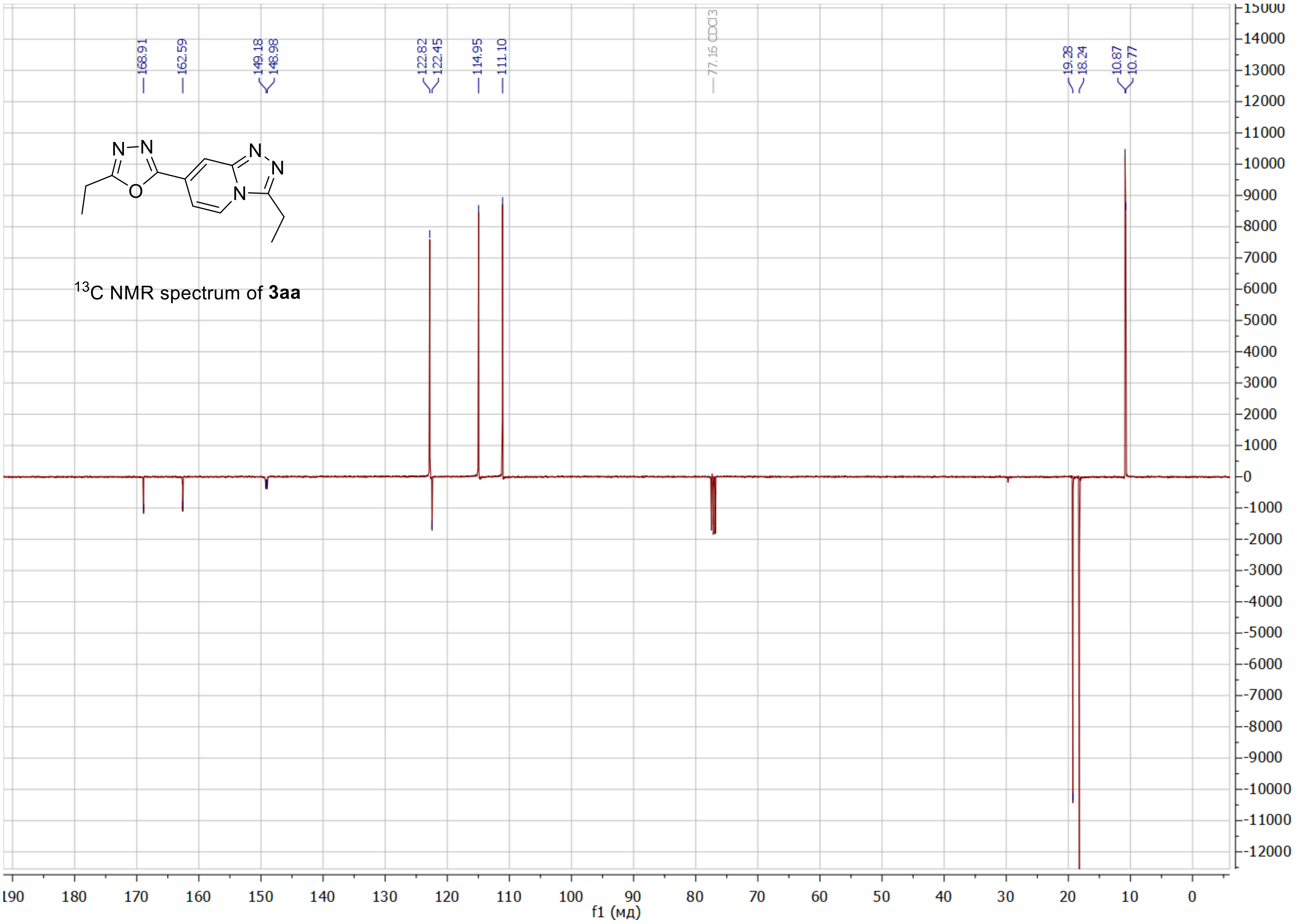

168.91
162.59
149.18
148.98
122.82
122.45
114.95
111.10
77.16 CDCl3
19.28
18.24
10.87
10.77
^{13}C NMR spectrum of **3aa**
f1 (мд)

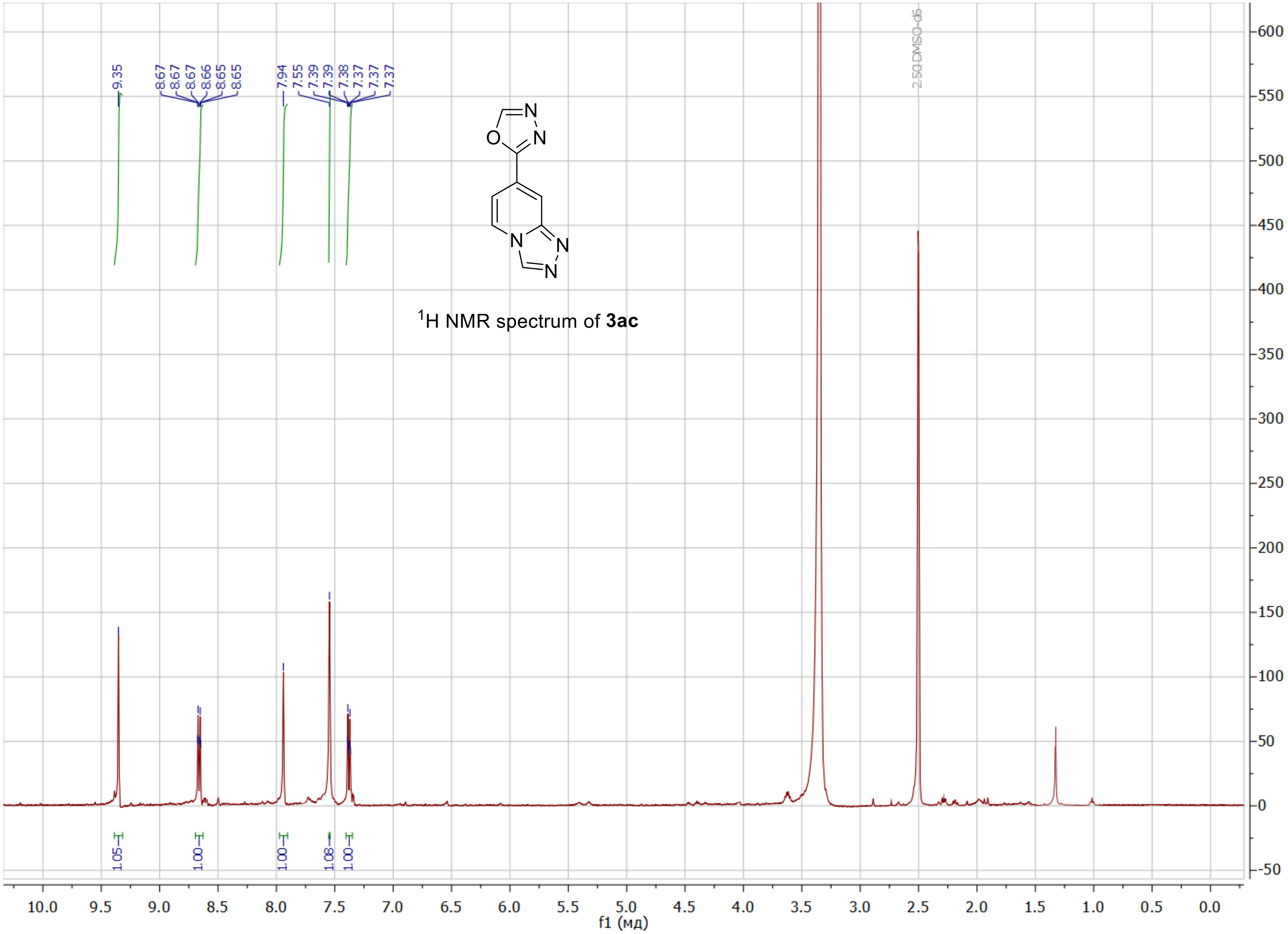

^{1}H NMR spectrum of **3ac**

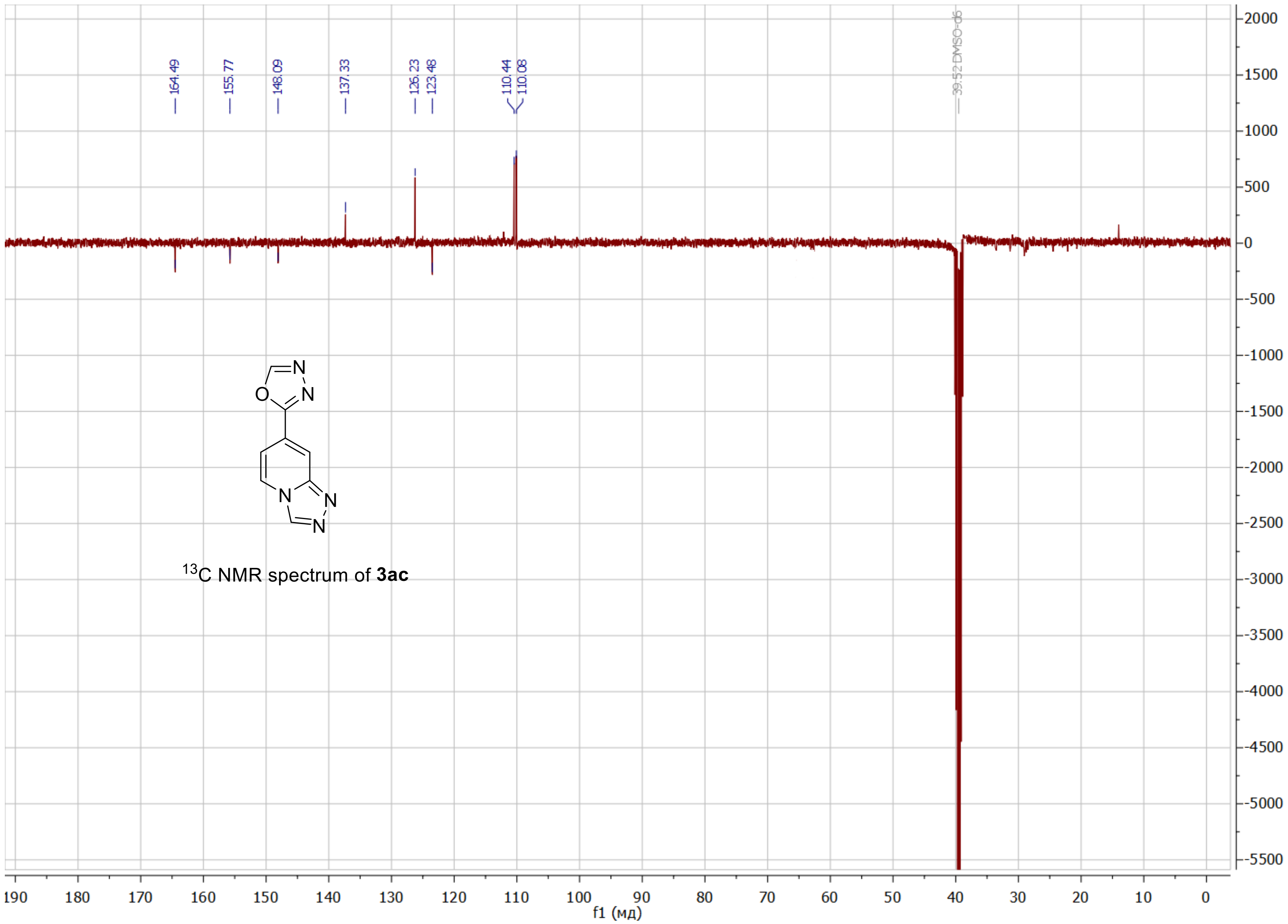

^{13}C NMR spectrum of **3ac**

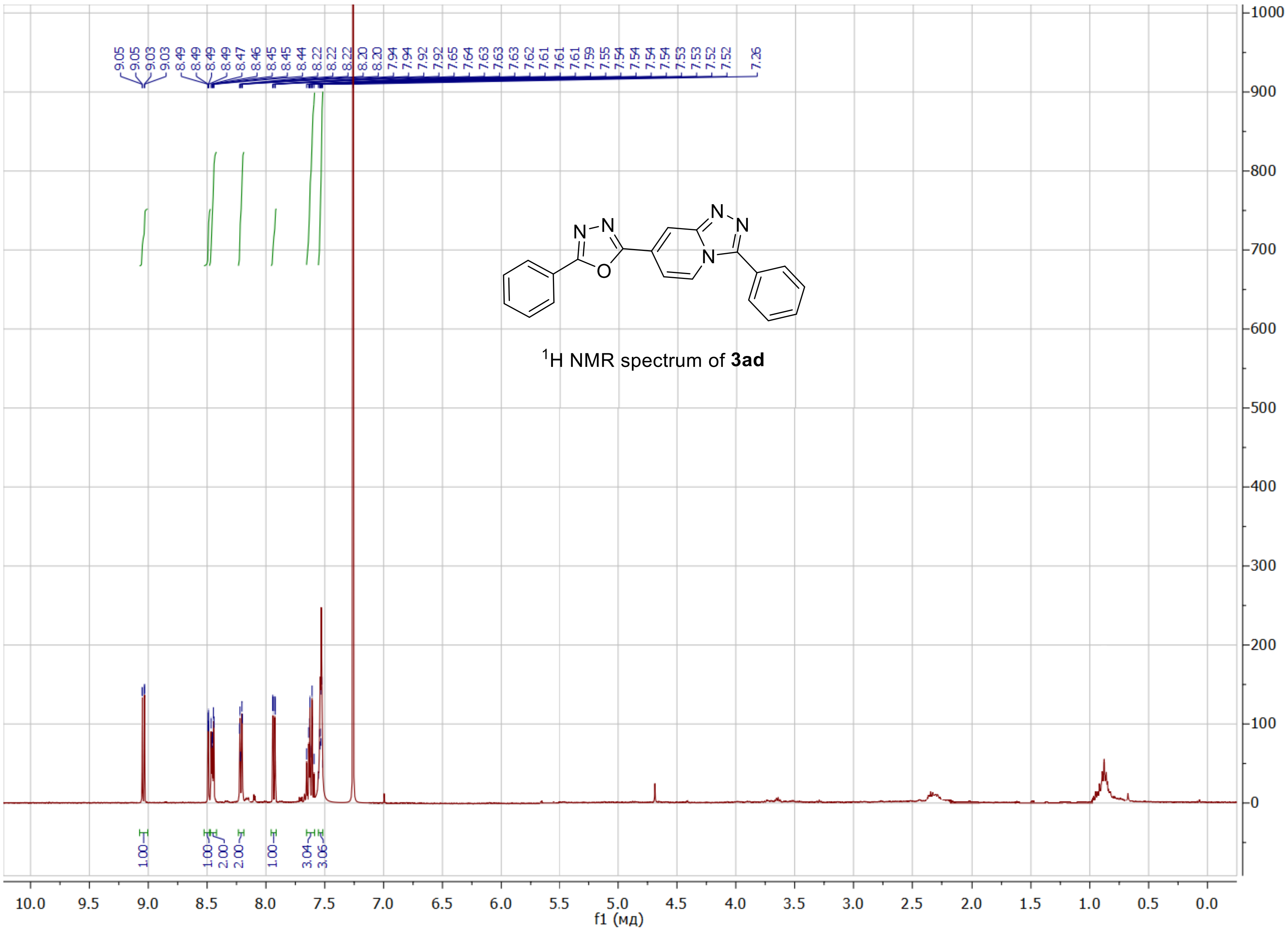

^{1}H NMR spectrum of **3ad**

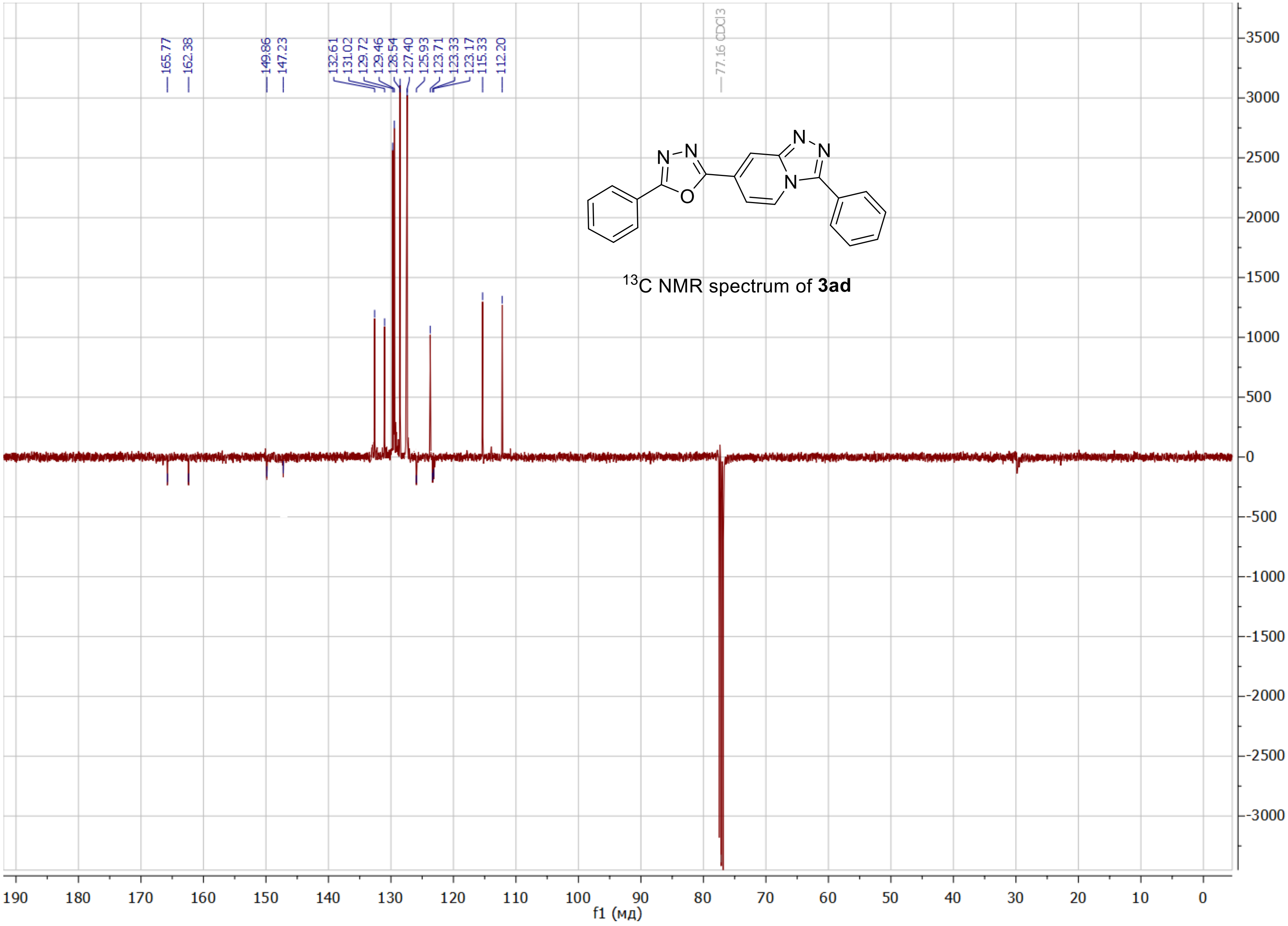

^{13}C NMR spectrum of **3ad**

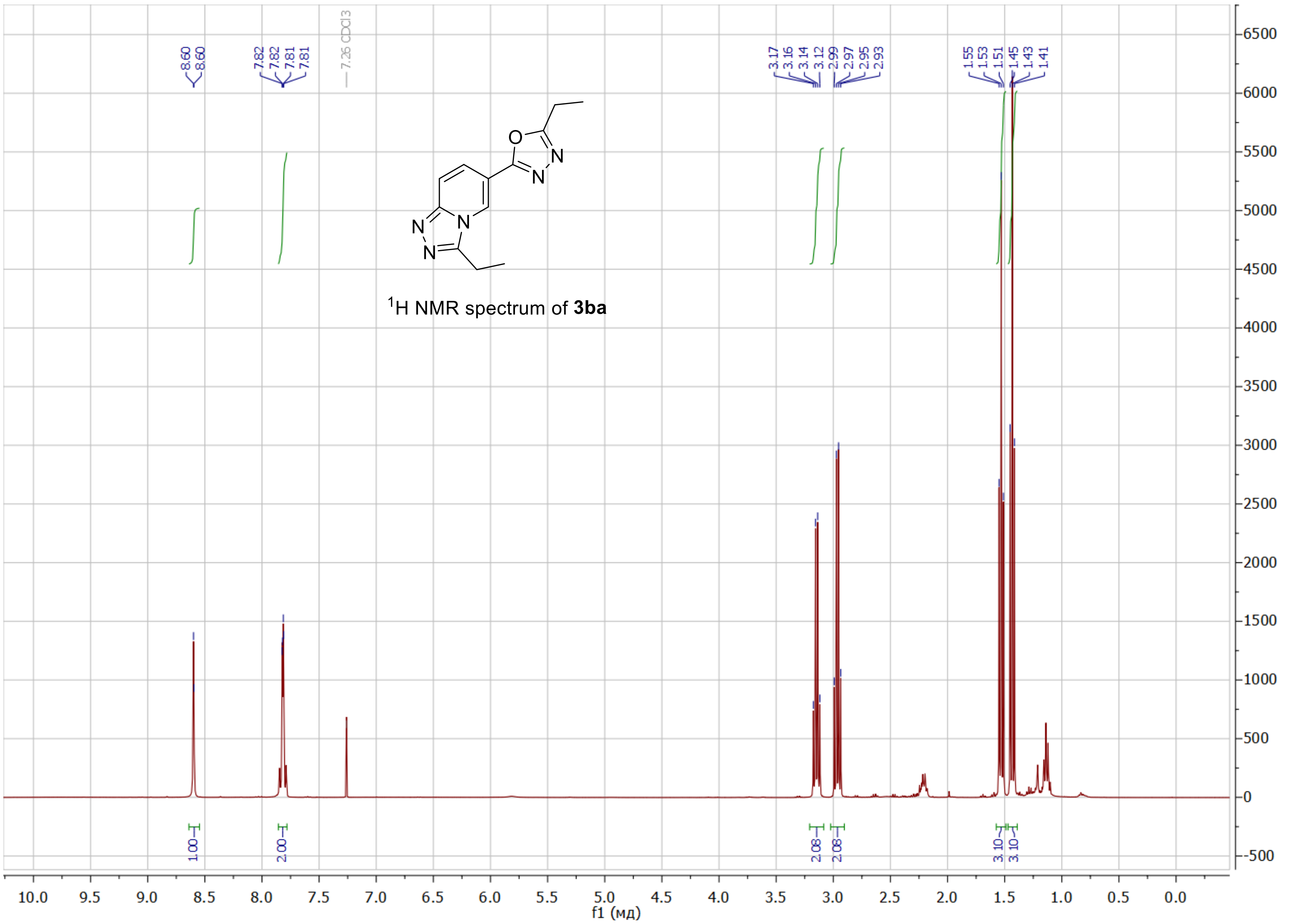
8.60
8.60
7.82
7.82
7.81
7.81
7.26 CDCl3
3.17
3.16
3.14
3.12
2.99
2.97
2.95
2.93
1.55
1.53
1.51
1.45
1.43
1.41
O
N
N
N
N
N
^{1}H NMR spectrum of **3ba**
1.00
2.00
2.08
2.08
3.10
3.10
f1 (мд)

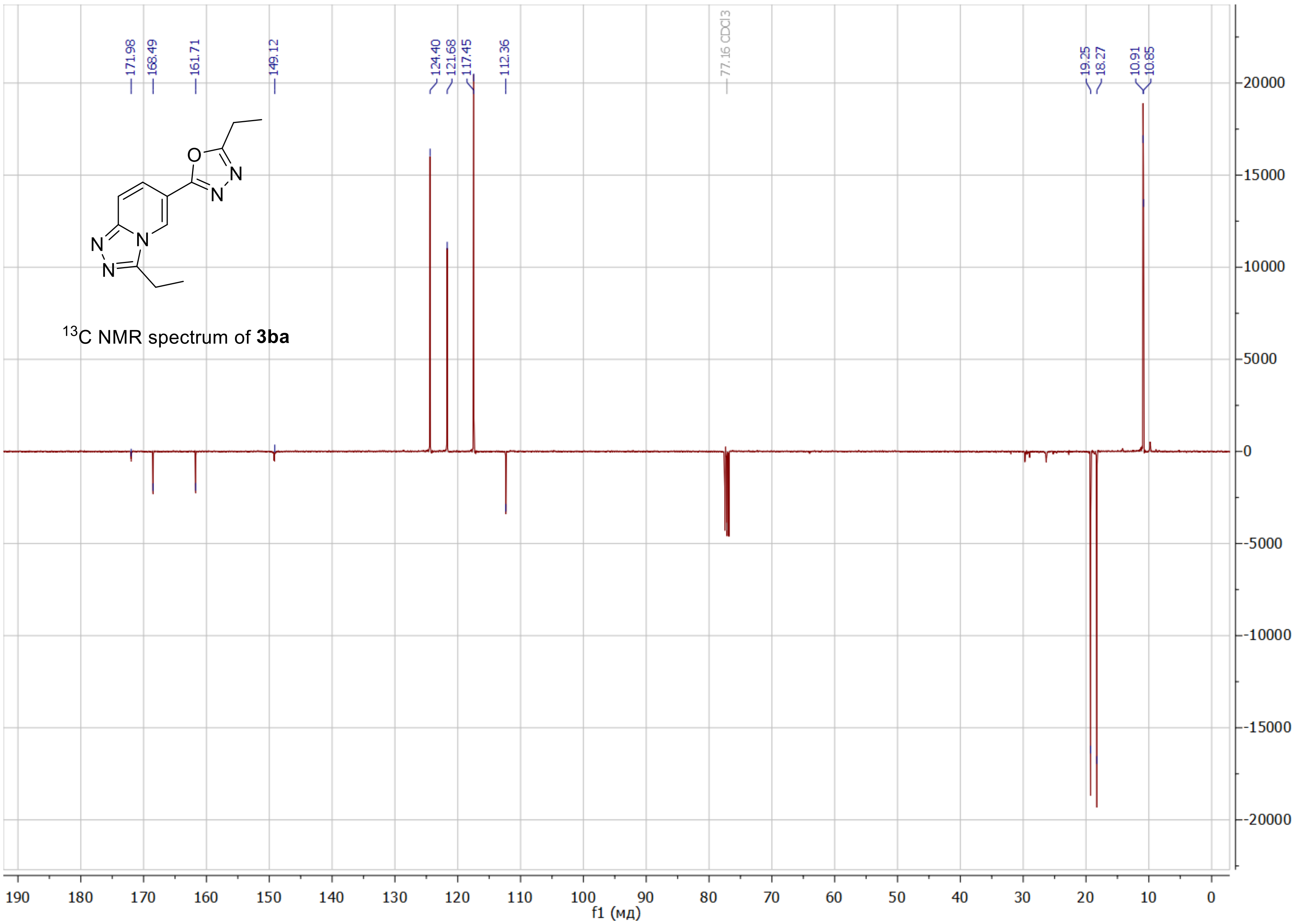
171.98
168.49
161.71
149.12
124.40
121.68
117.45
112.36
77.16 CDCl3
19.25
18.27
10.91
10.85
13C NMR spectrum of 3ba
f1 (мд)

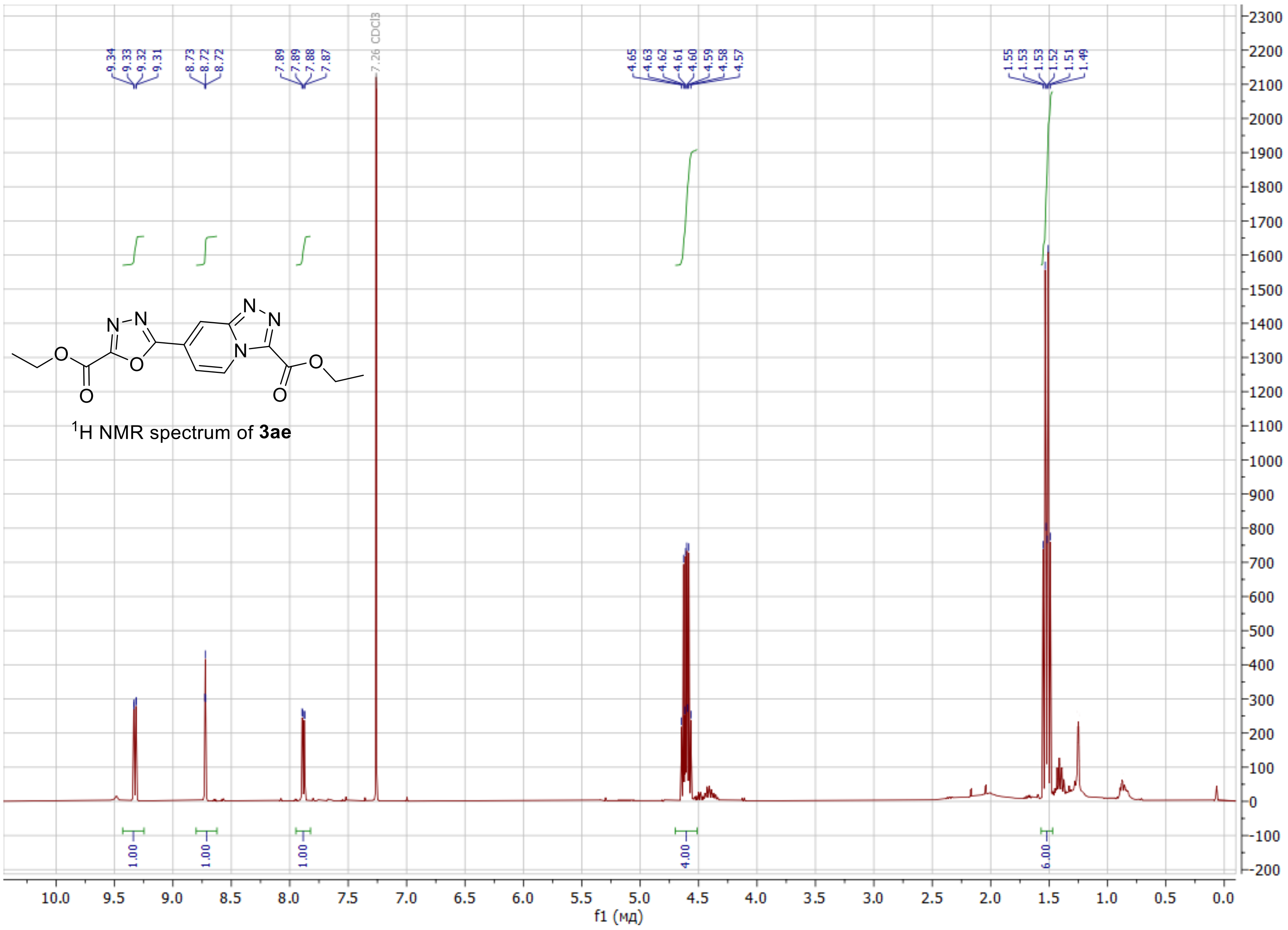
9.34
9.33
9.32
9.31
8.73
8.72
8.72
7.89
7.89
7.88
7.87
7.26 CDCl3
4.65
4.63
4.62
4.61
4.60
4.59
4.58
4.57
1.55
1.53
1.53
1.52
1.51
1.49
1.00
1.00
1.00
4.00
6.00
f1 (мд)
^{1}H NMR spectrum of **3ae**

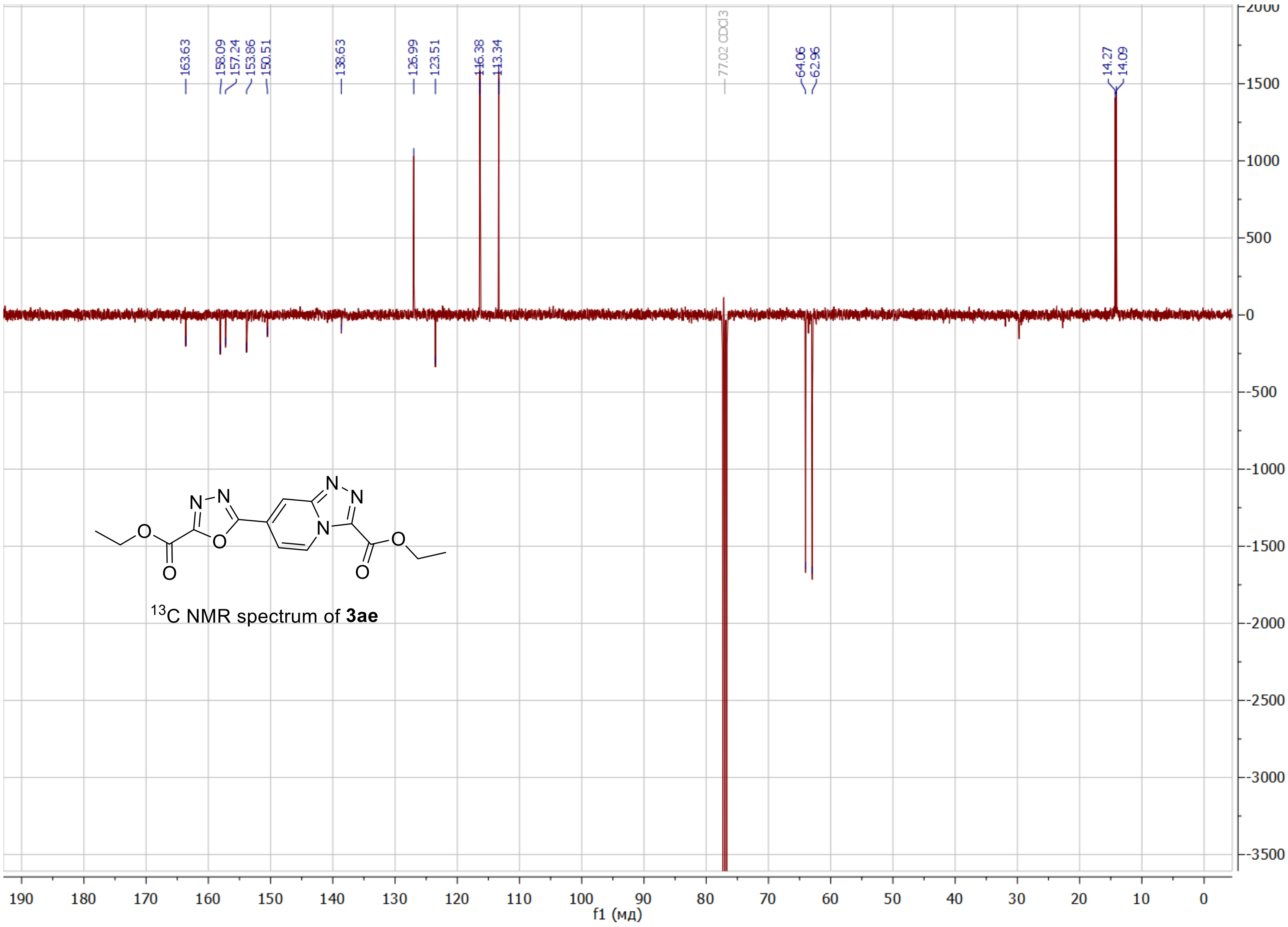

^{13}C NMR spectrum of **3ae**

^{1}H and ^{13}C NMR spectral charts for 1,3,4-oxadiazol-2-yl)[1,2,4]triazolo[4,3-a]quinolines

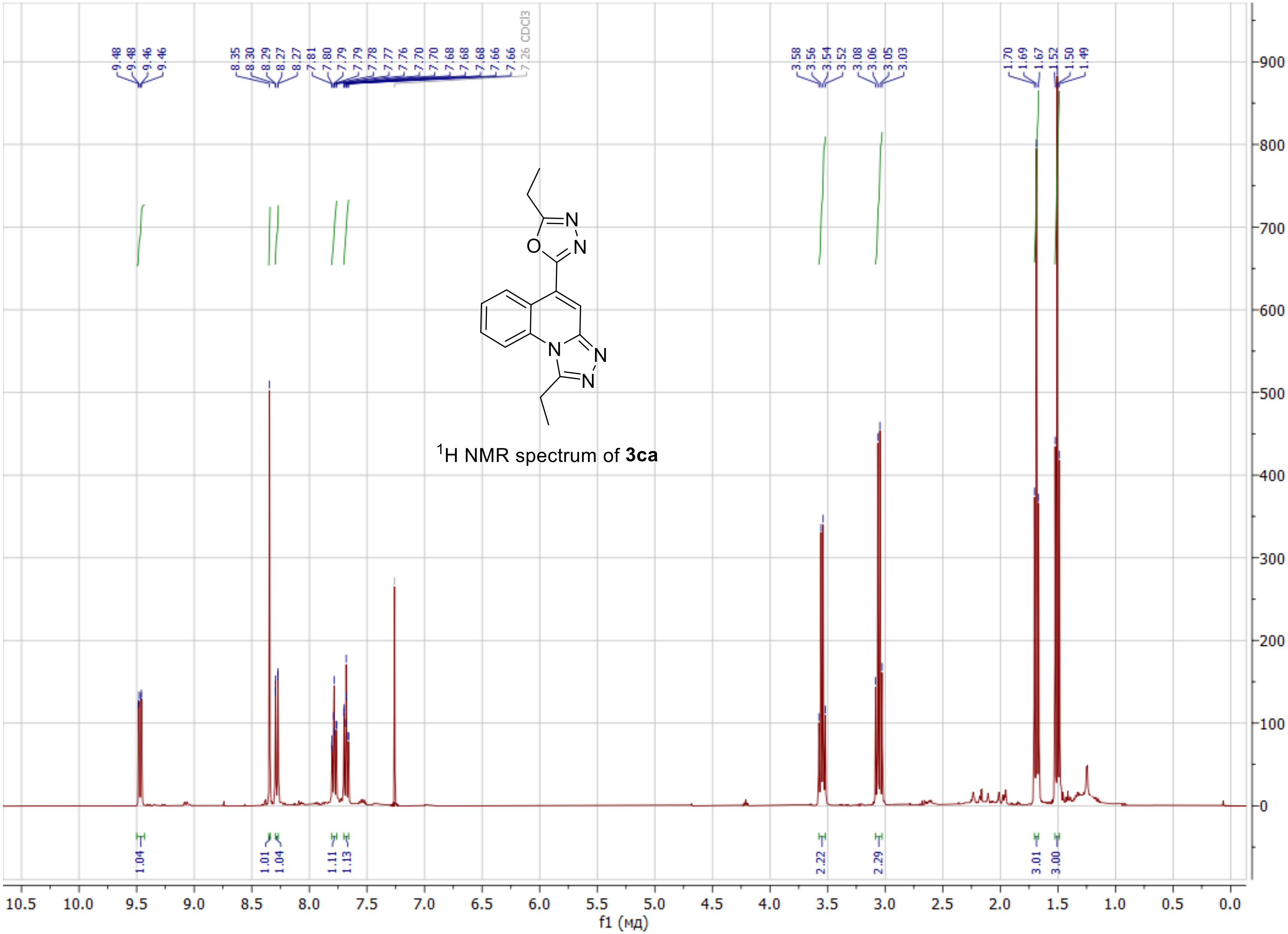

^{1}H NMR spectrum of **3ca**

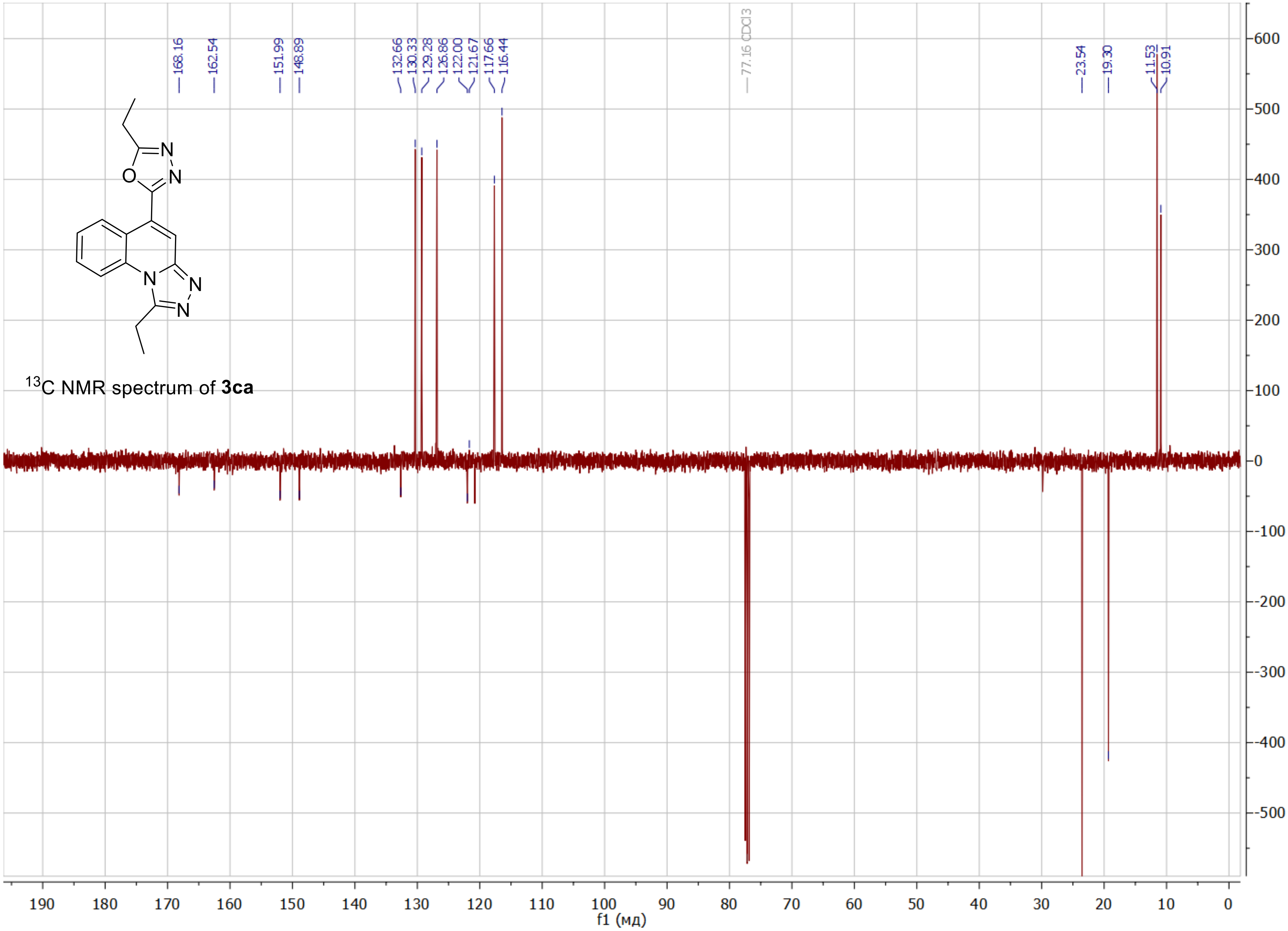

^{13}C NMR spectrum of **3ca**

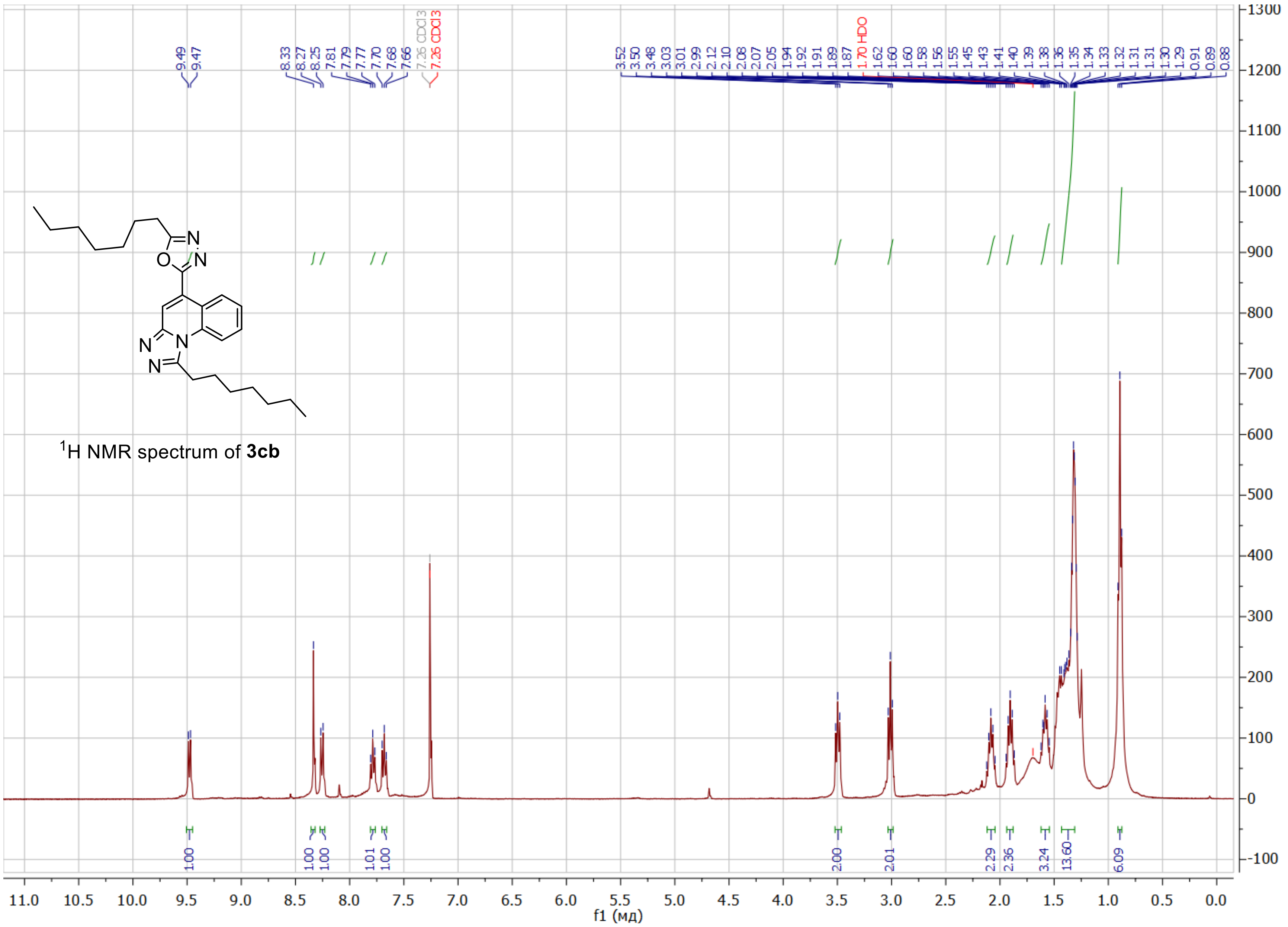

^{1}H NMR spectrum of **3cb**

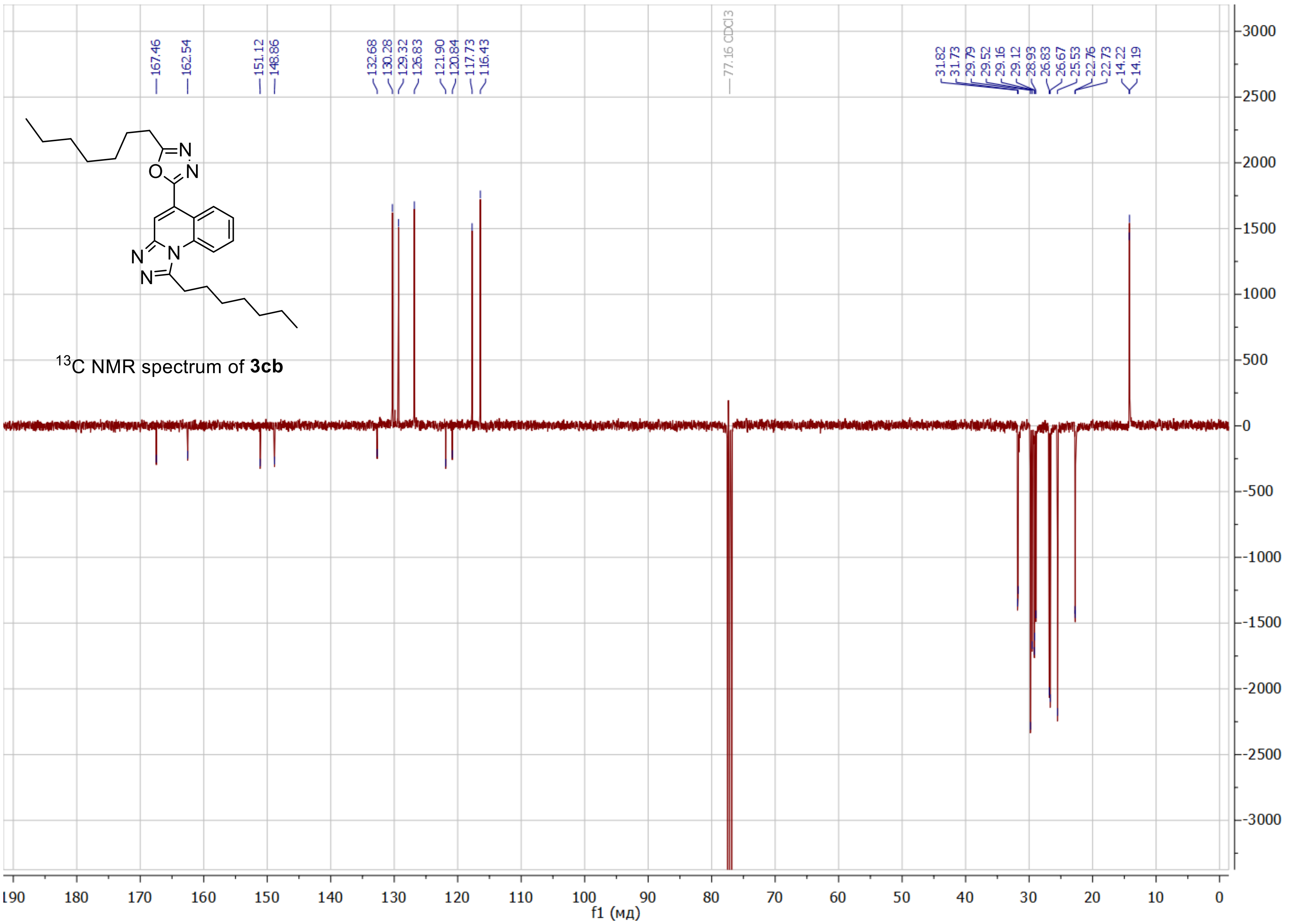

167.46
162.54
151.12
148.86
132.68
130.28
129.32
126.83
121.90
120.84
117.73
116.43
77.16 CDCl3
31.82
31.73
29.79
29.52
29.16
29.12
28.93
26.83
26.67
25.53
22.76
22.73
14.22
14.19
^{13}C NMR spectrum of **3cb**
f1 (мд)

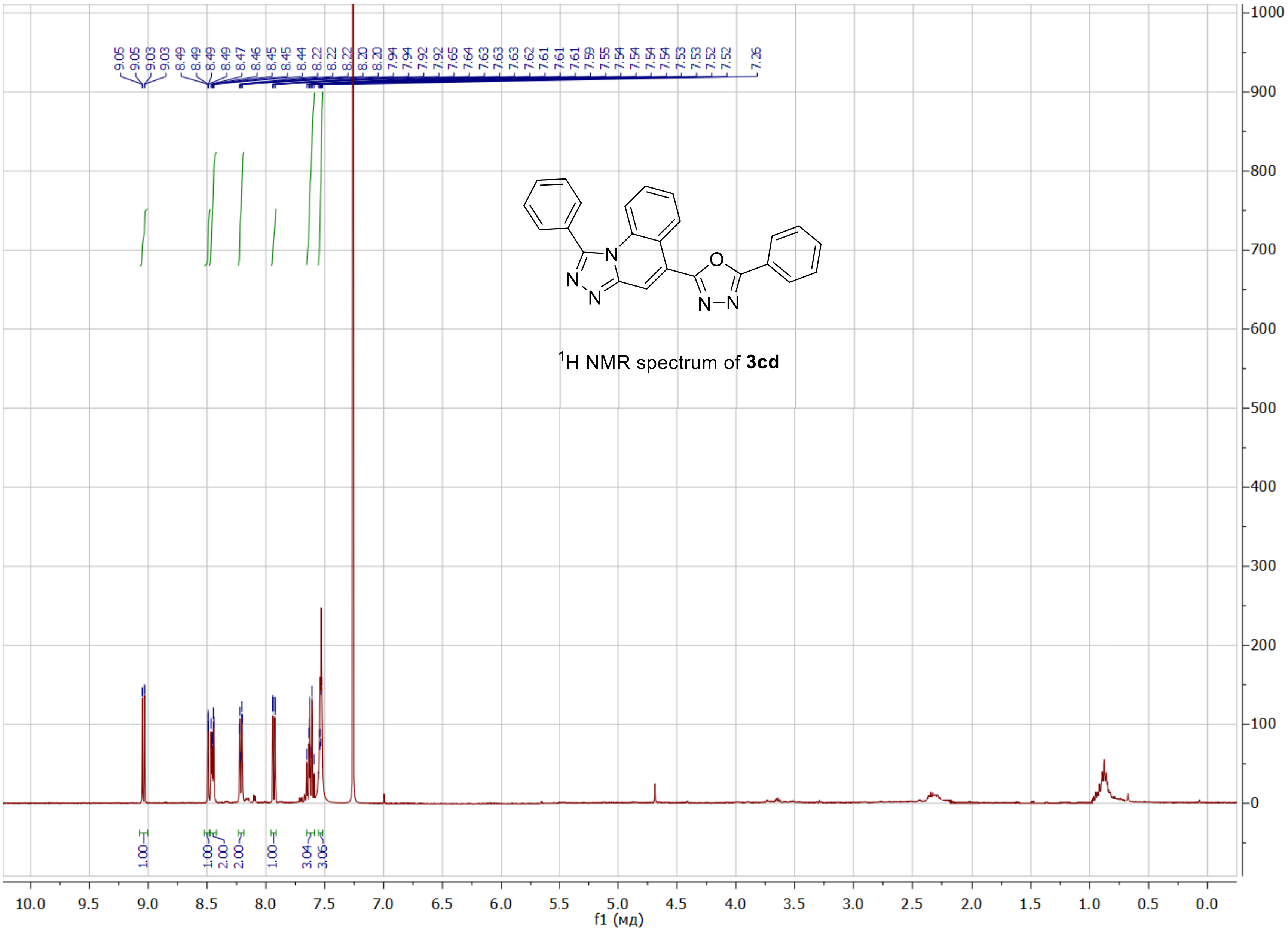

9.05 9.05 9.03 9.03 8.49 8.49 8.49 8.49 8.47 8.46 8.45 8.45 8.44 8.22 8.22 8.22 8.20 8.20 7.94 7.94 7.92 7.92 7.65 7.64 7.63 7.63 7.62 7.61 7.61 7.61 7.59 7.55 7.54 7.54 7.54 7.54 7.53 7.53 7.52 7.52 7.26
1.00 1.00 2.00 2.00 1.00 3.04 3.06
N
N
N
O
N
N
^{1}H NMR spectrum of **3cd**
f1 (мд)

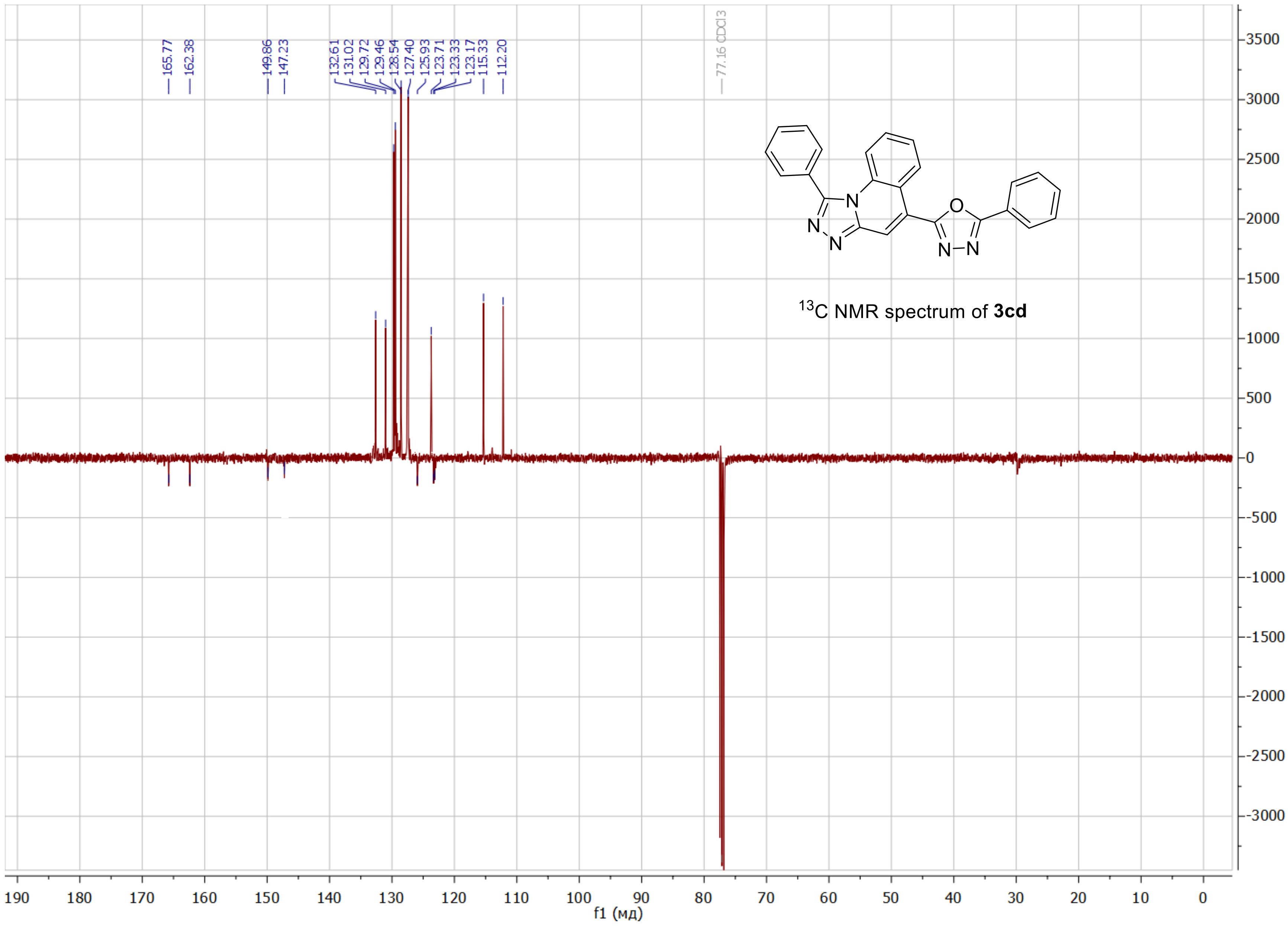

165.77
162.38
149.86
147.23
132.61
131.02
129.72
129.46
128.54
127.40
125.93
123.71
123.33
123.17
115.33
112.20
77.16 CDCl3
N
N
N
O
N
N
^{13}C NMR spectrum of **3cd**
f1 (мд)

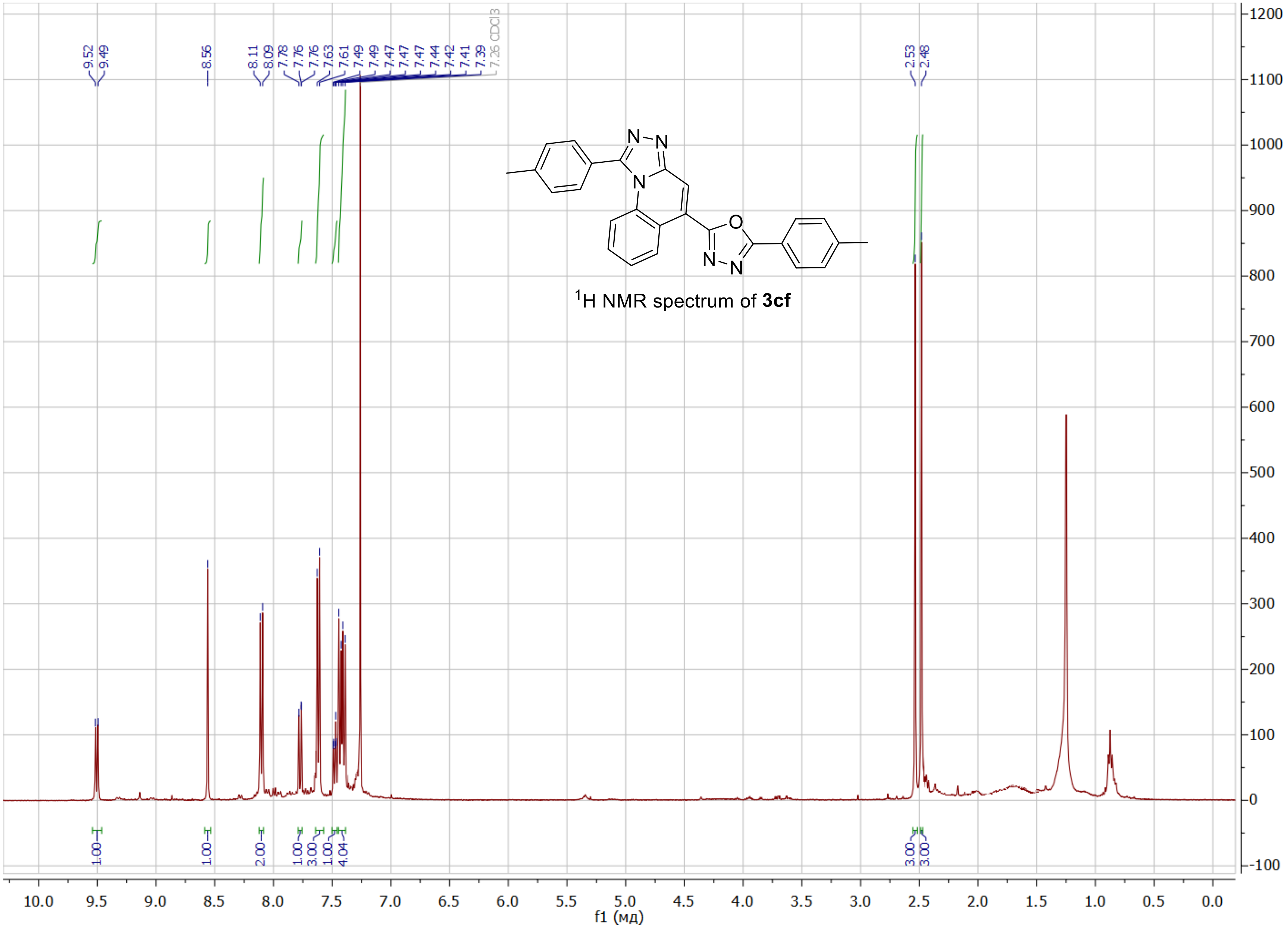

^{1}H NMR spectrum of **3cf**

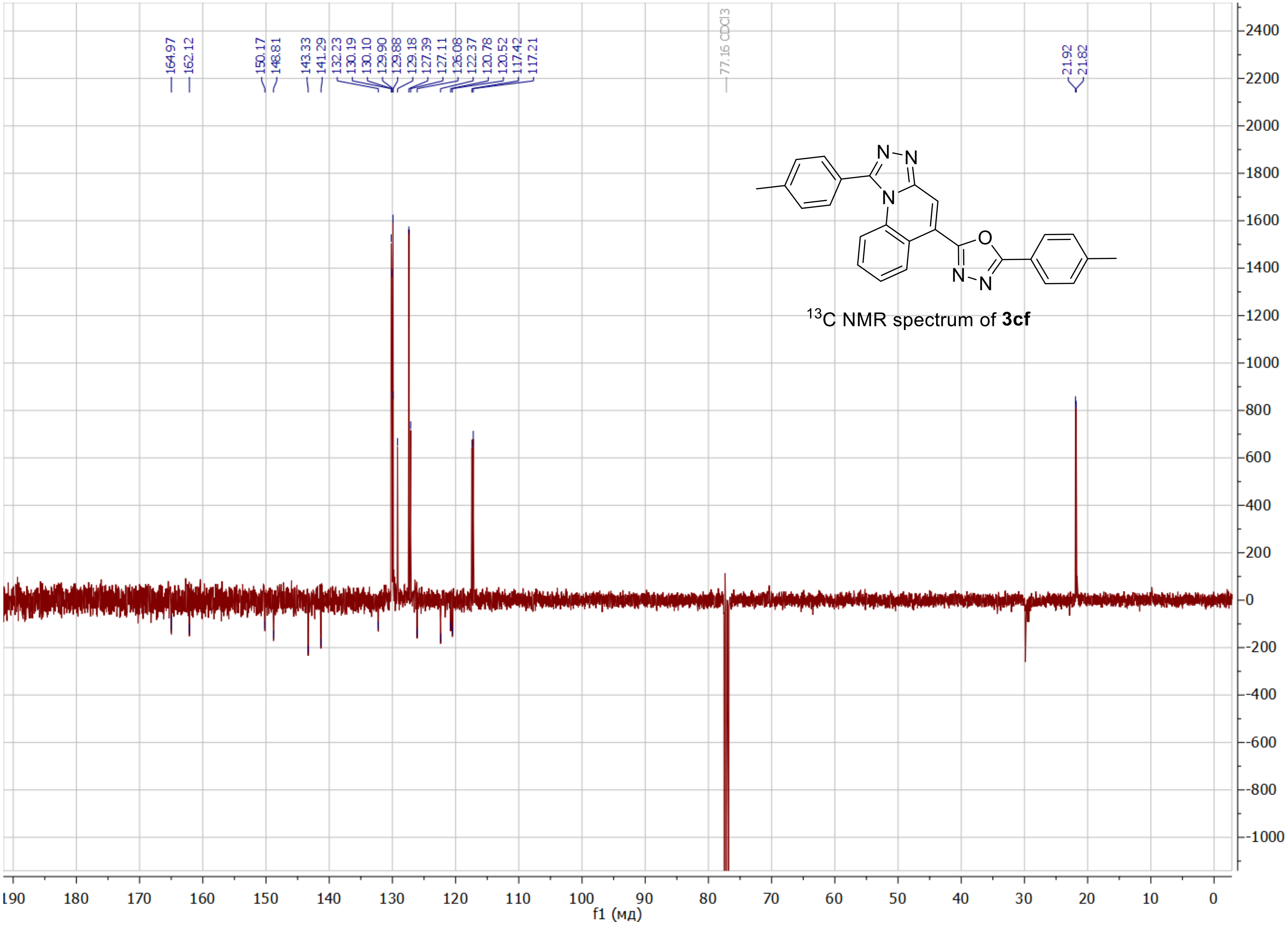

^{13}C NMR spectrum of **3cf**

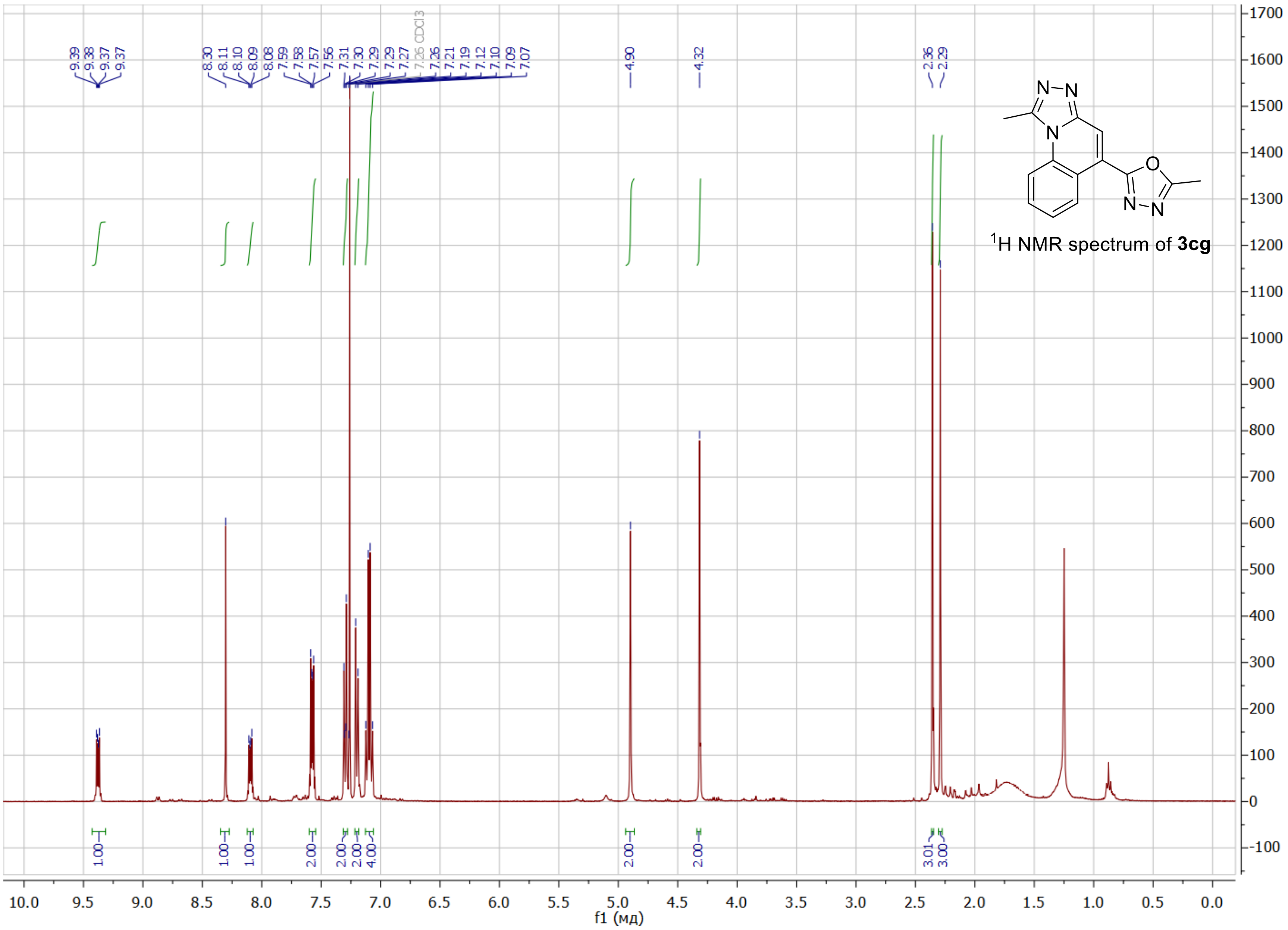

9.39
9.38
9.37
9.37
8.30
8.11
8.10
8.09
8.08
7.59
7.58
7.57
7.56
7.31
7.30
7.29
7.29
7.27
7.26 CDCl3
7.26
7.21
7.19
7.12
7.10
7.09
7.07
4.90
4.32
2.36
2.29
1.00
1.00
1.00
2.00
2.00
2.00
4.00
2.00
2.00
3.01
3.00
f1 (мд)

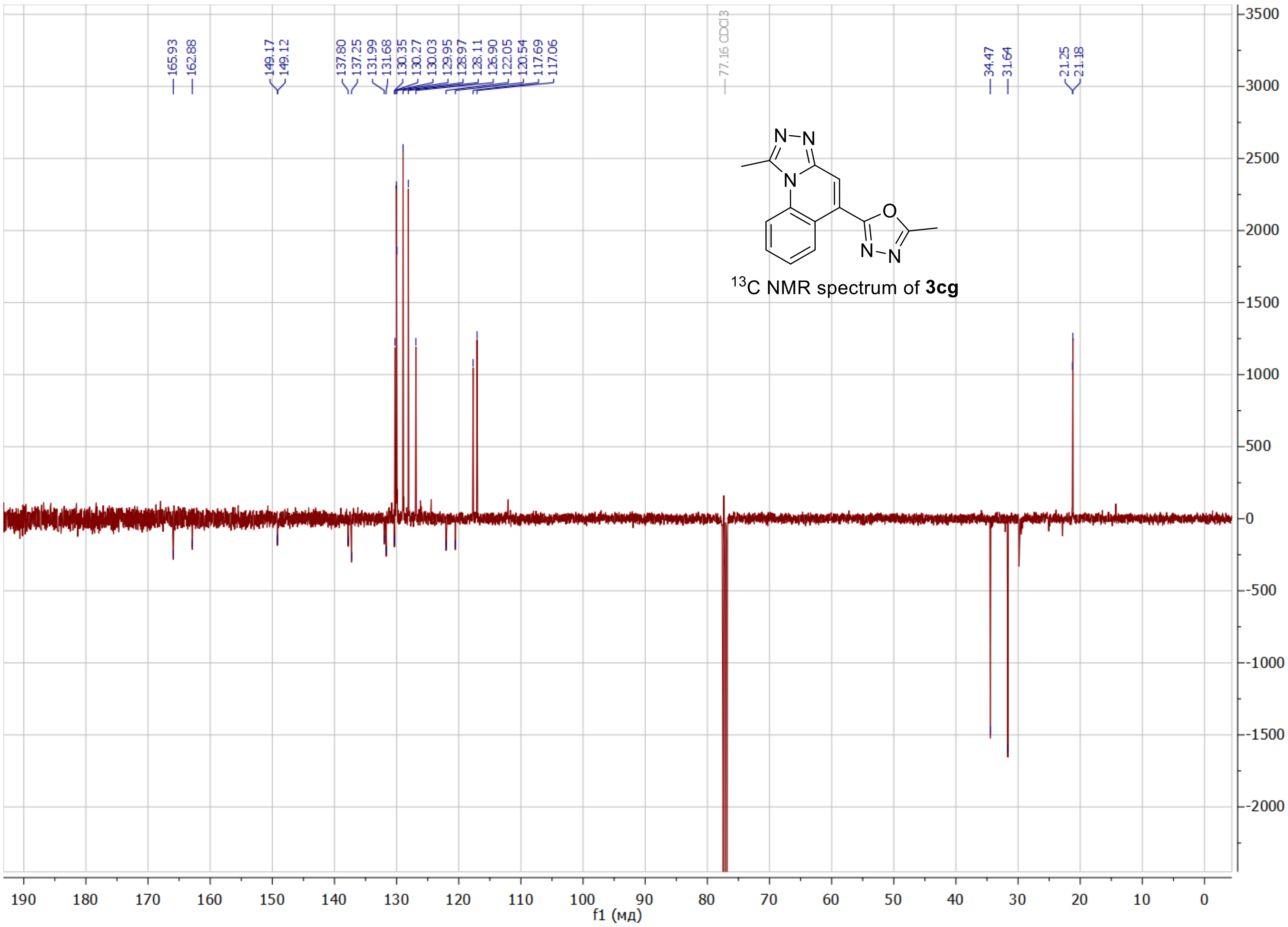

165.93
162.88
149.17
149.12
137.80
137.25
131.99
131.68
130.35
130.27
130.03
129.95
128.97
128.11
126.90
122.05
120.54
117.69
117.06
77.16 CDCl3
34.47
31.64
21.25
21.18
^{13}C NMR spectrum of **3cg**
f1 (мд)

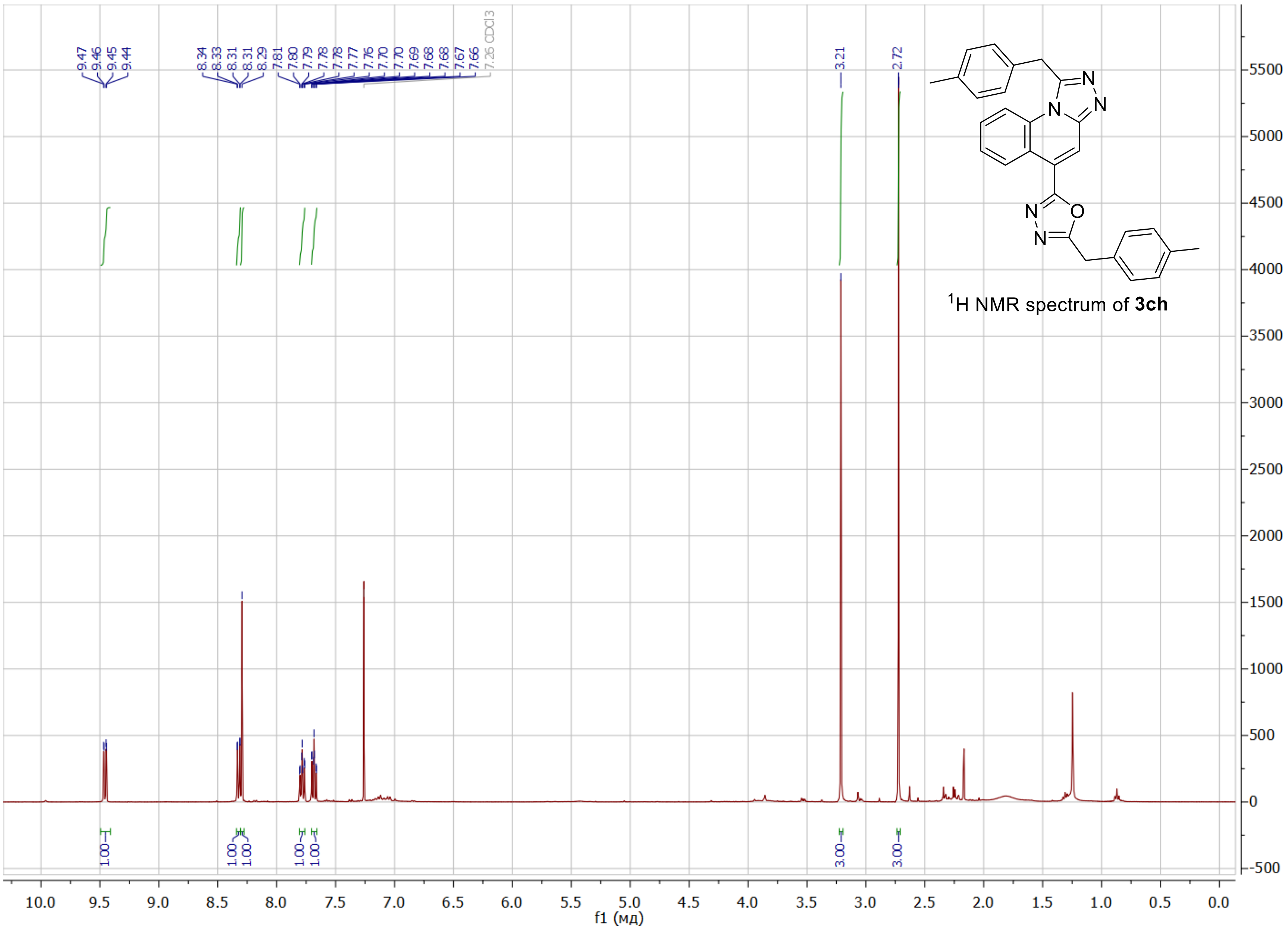

^{1}H NMR spectrum of **3ch**

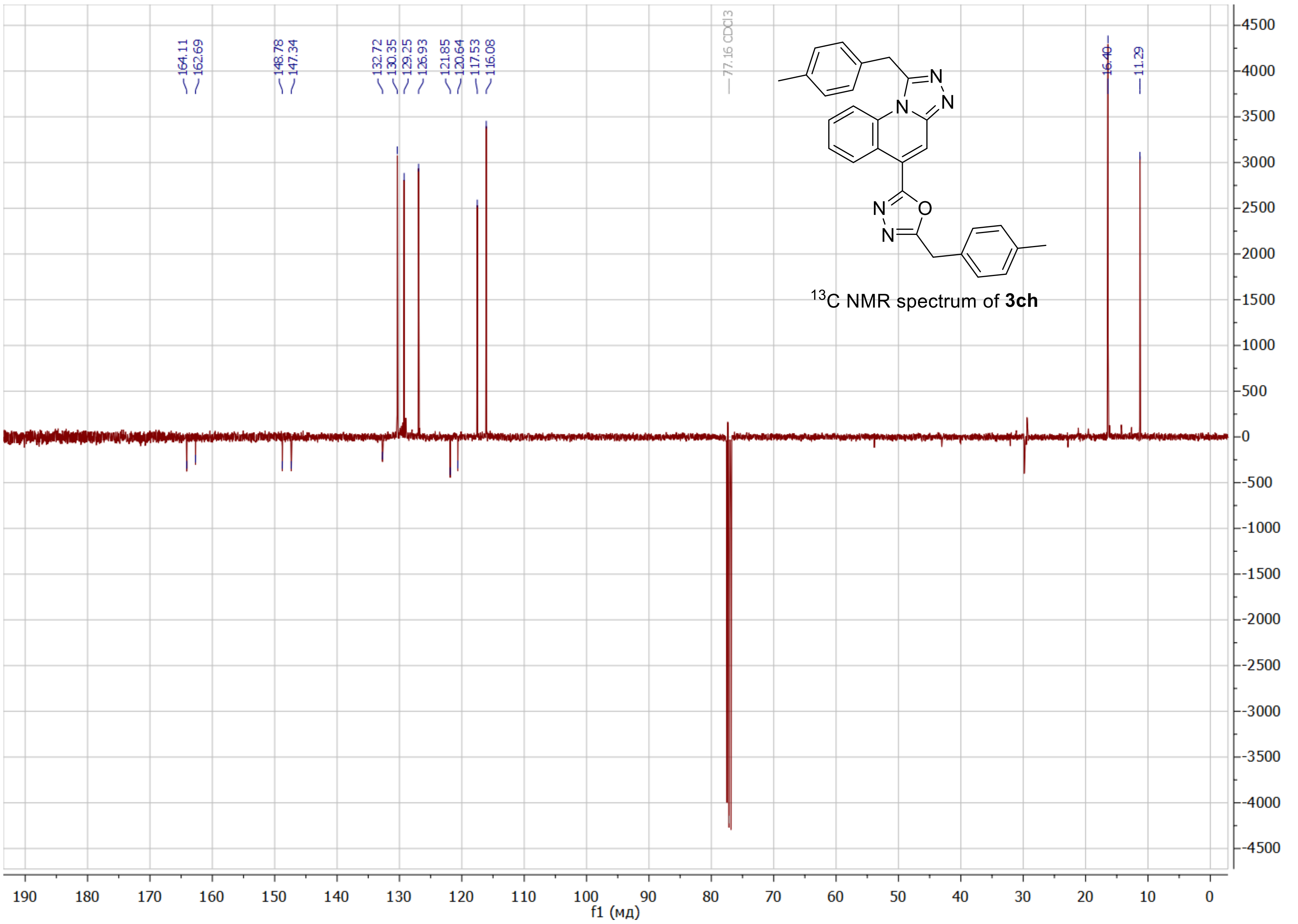

^{13}C NMR spectrum of **3ch**

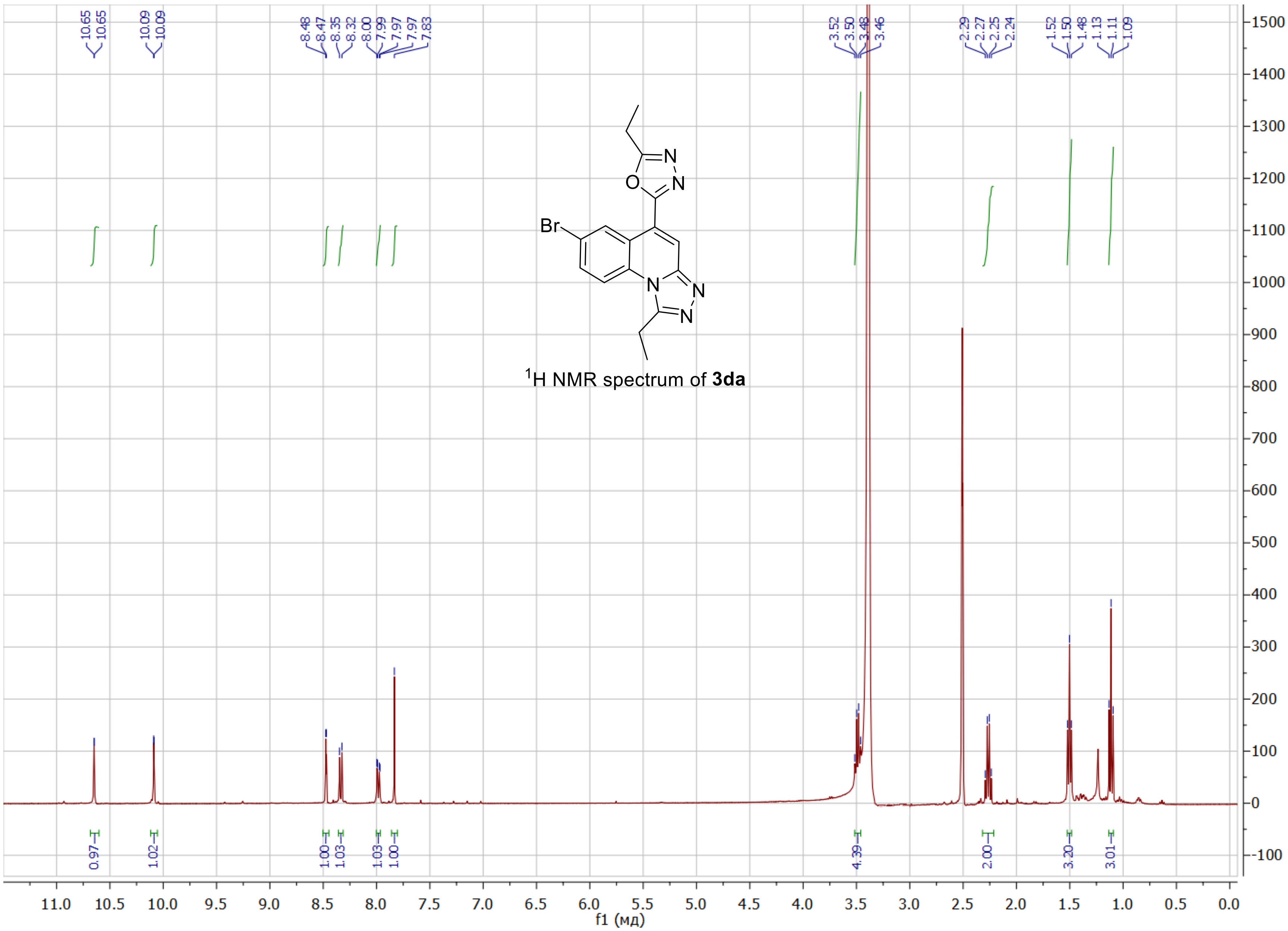

^{1}H NMR spectrum of **3da**

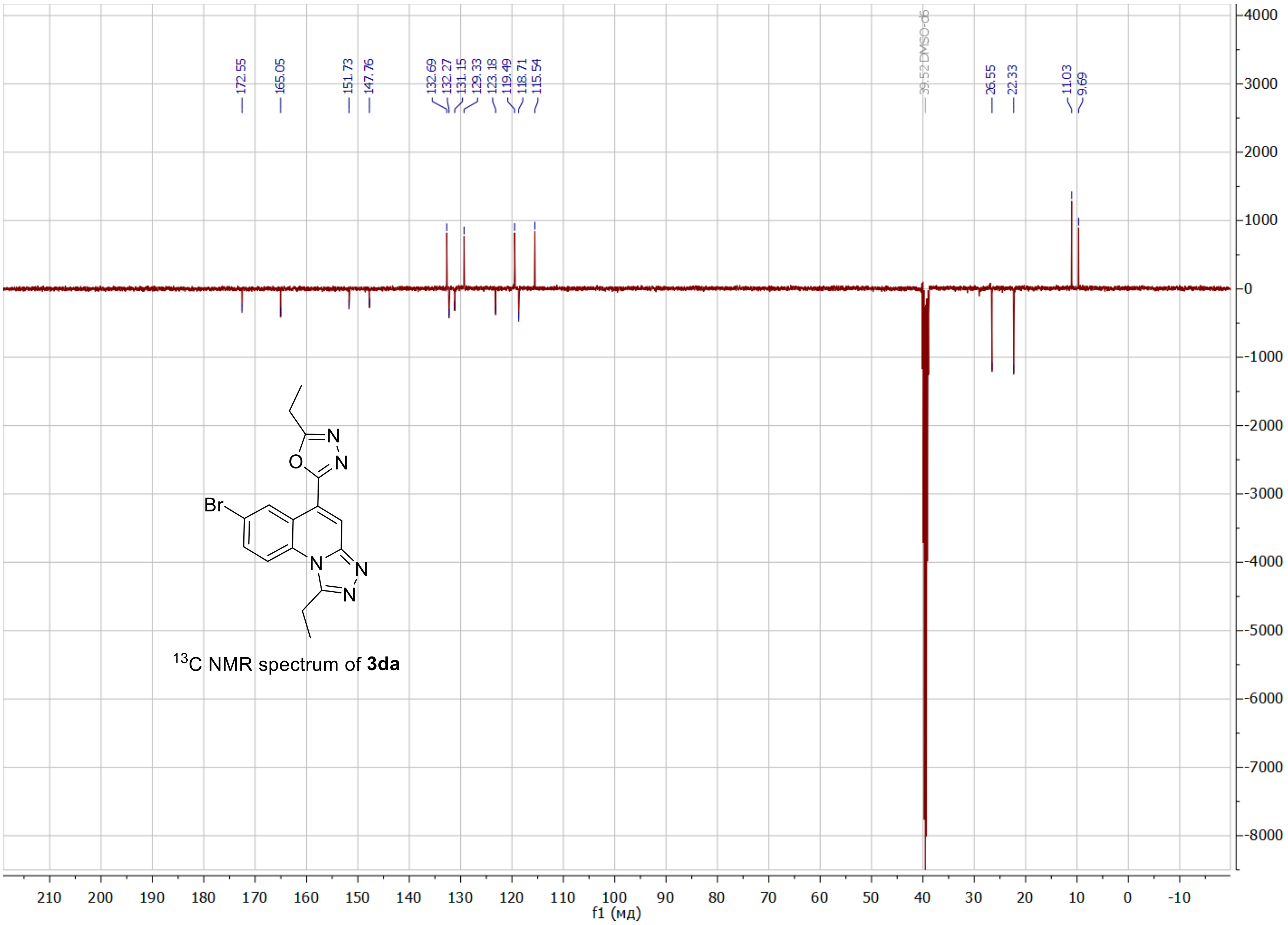

^{13}C NMR spectrum of **3da**

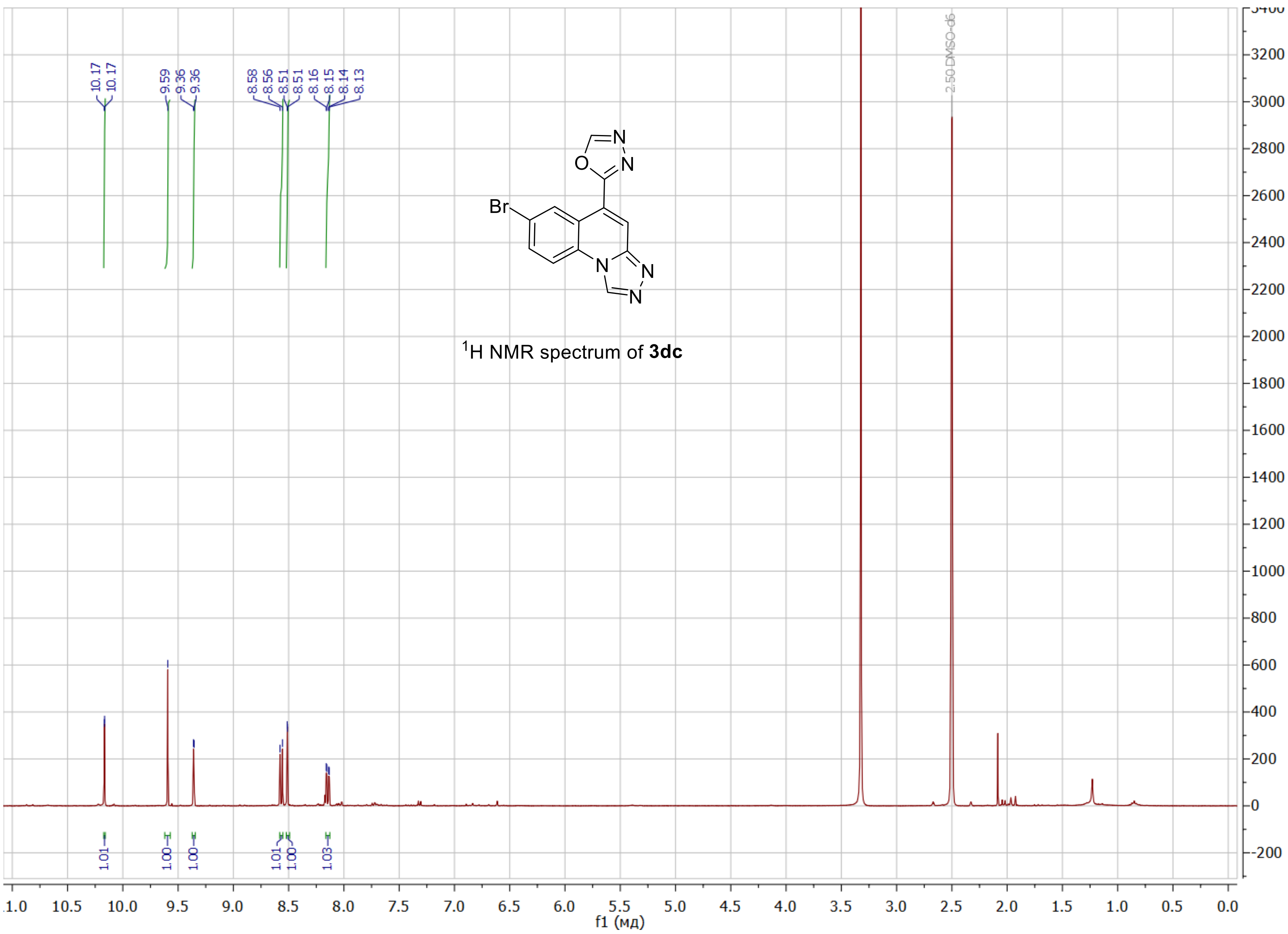

^{1}H NMR spectrum of **3dc**

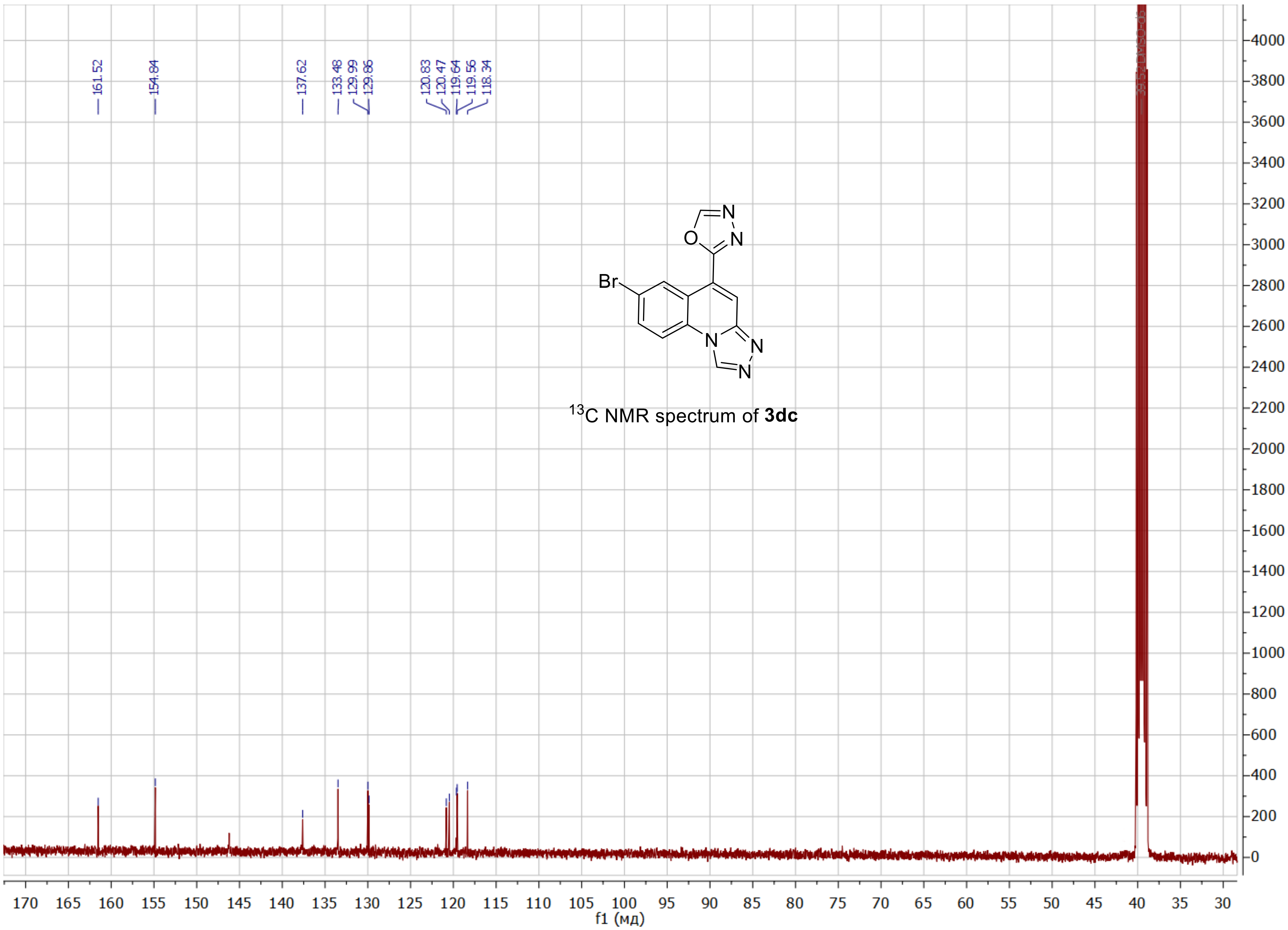

^{13}C NMR spectrum of **3dc**

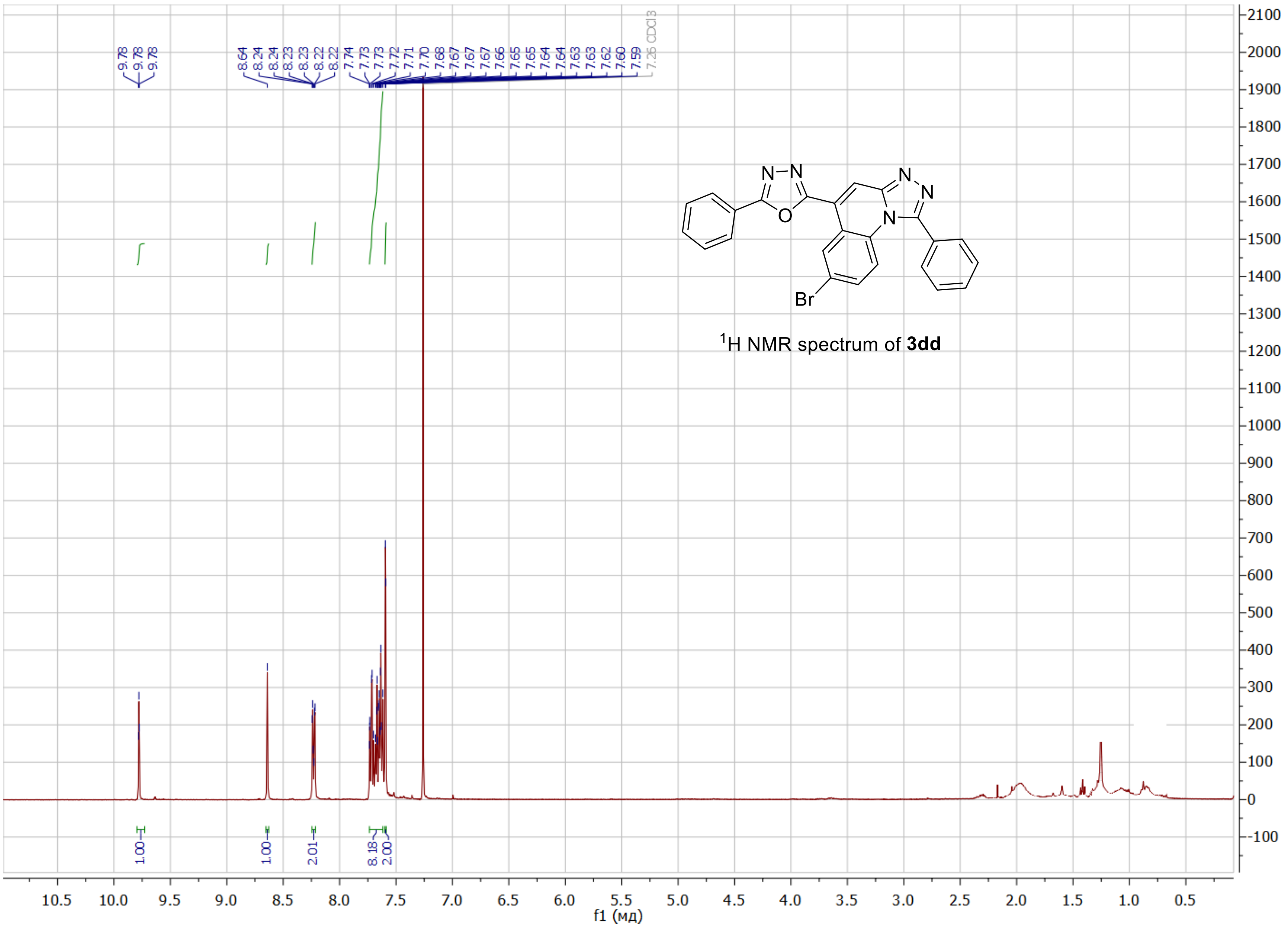

Br
N
O
1H NMR spectrum of 3dd
f1 (мд)
7.26 CDCl3
1.00
1.00
2.01
8.18
2.00

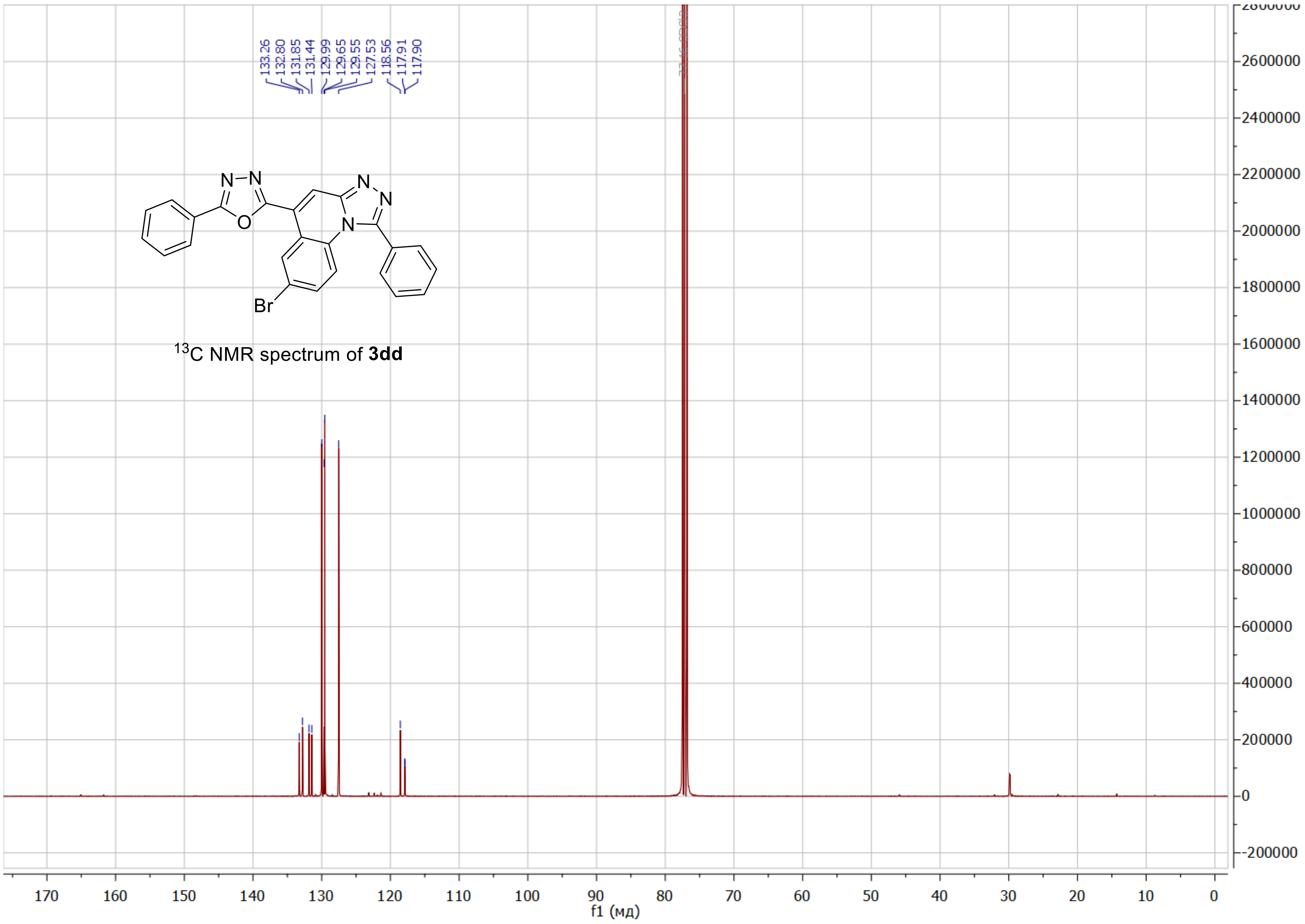
133.26
132.80
131.85
131.44
129.99
129.65
129.55
127.53
118.56
117.91
117.90
N
N
O
N
N
N
Br
^{13}C NMR spectrum of **3dd**
f1 (мд)

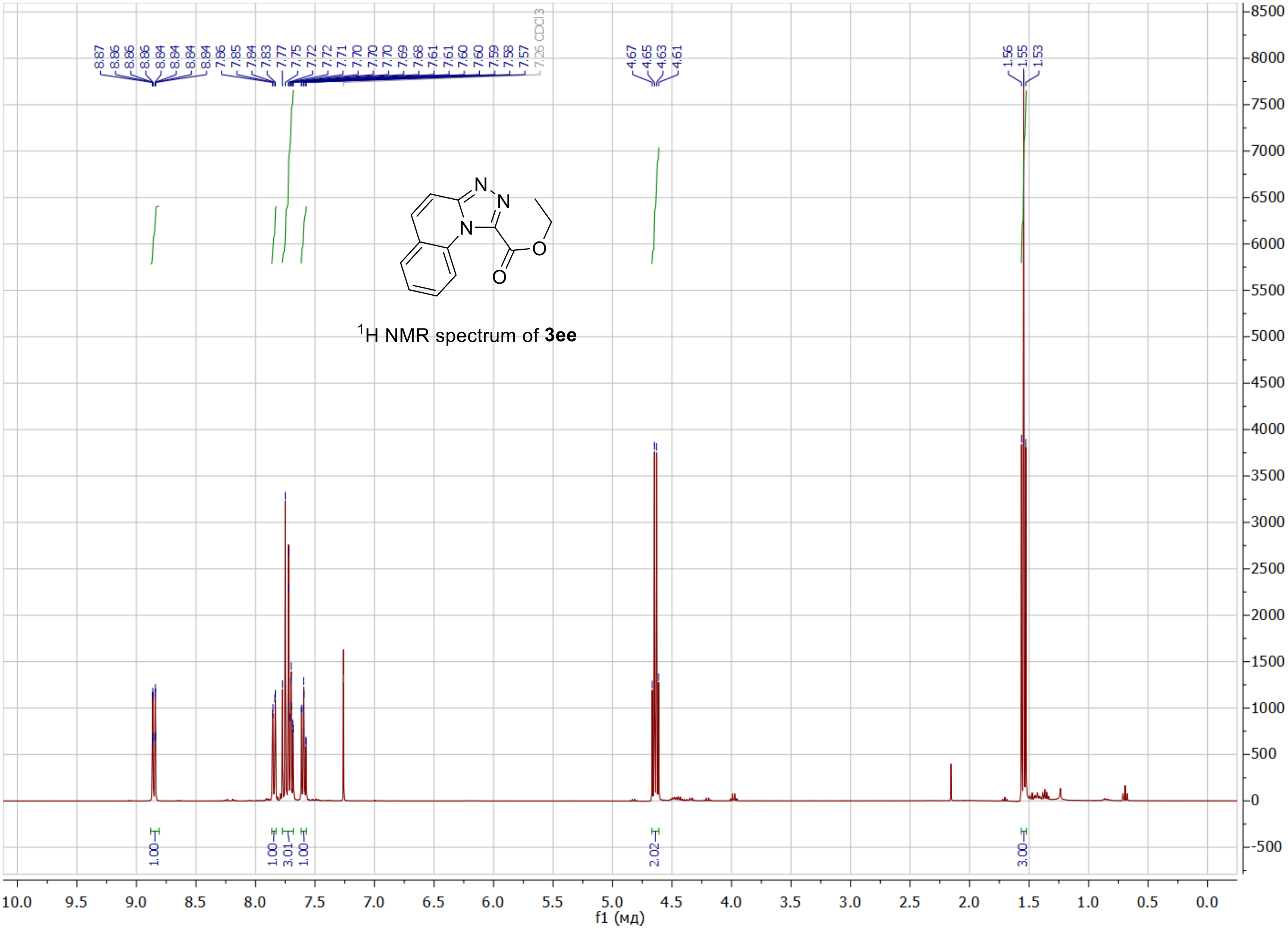

1H NMR spectrum of 3ee
f1 (мд)
7.26 CDCl3

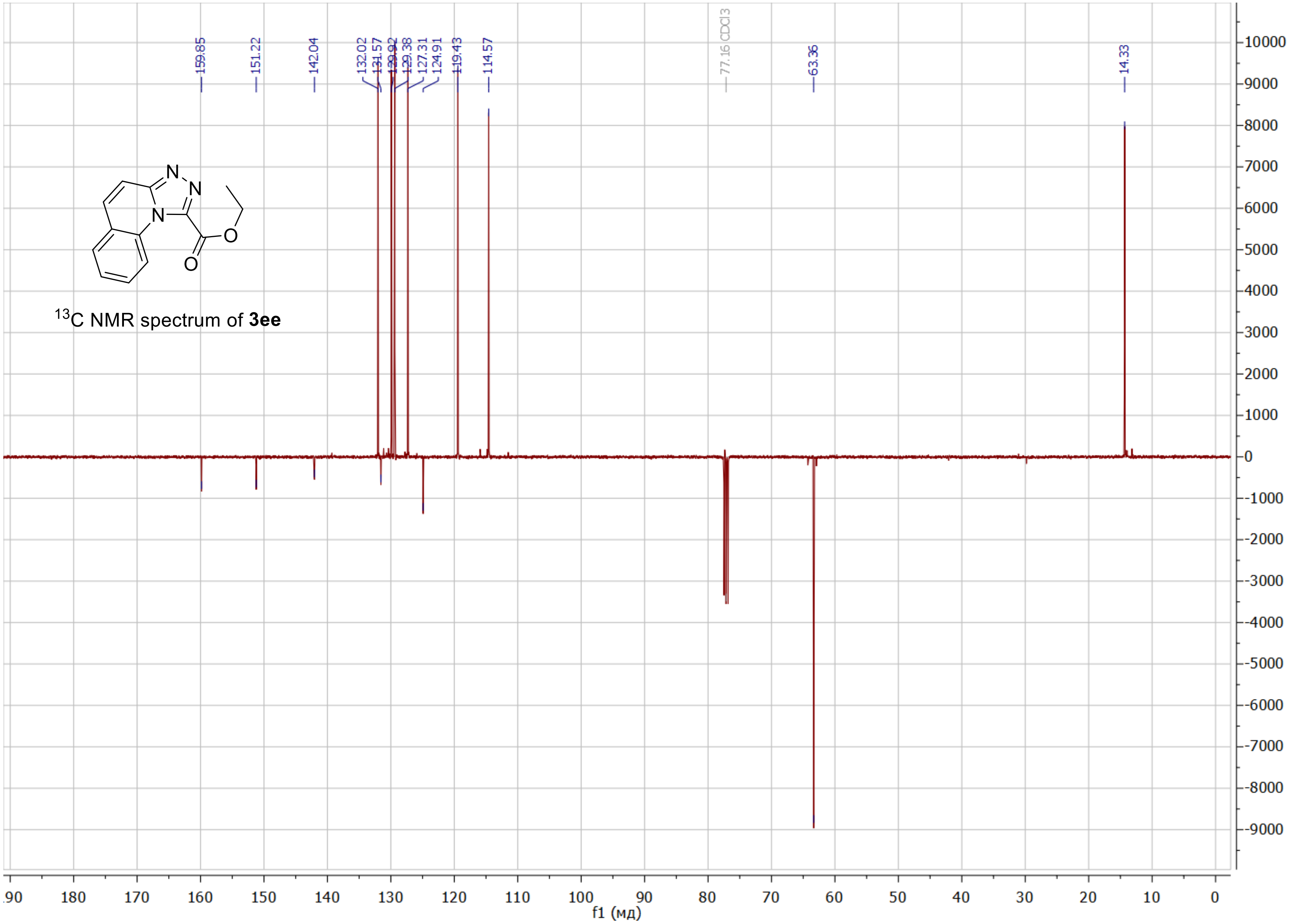

159.85
151.22
142.04
132.02
131.57
129.92
129.38
127.31
124.91
119.43
114.57
77.16 CDCl3
63.36
14.33
N
N
N
O
O
^{13}C NMR spectrum of **3ee**
f1 (мд)

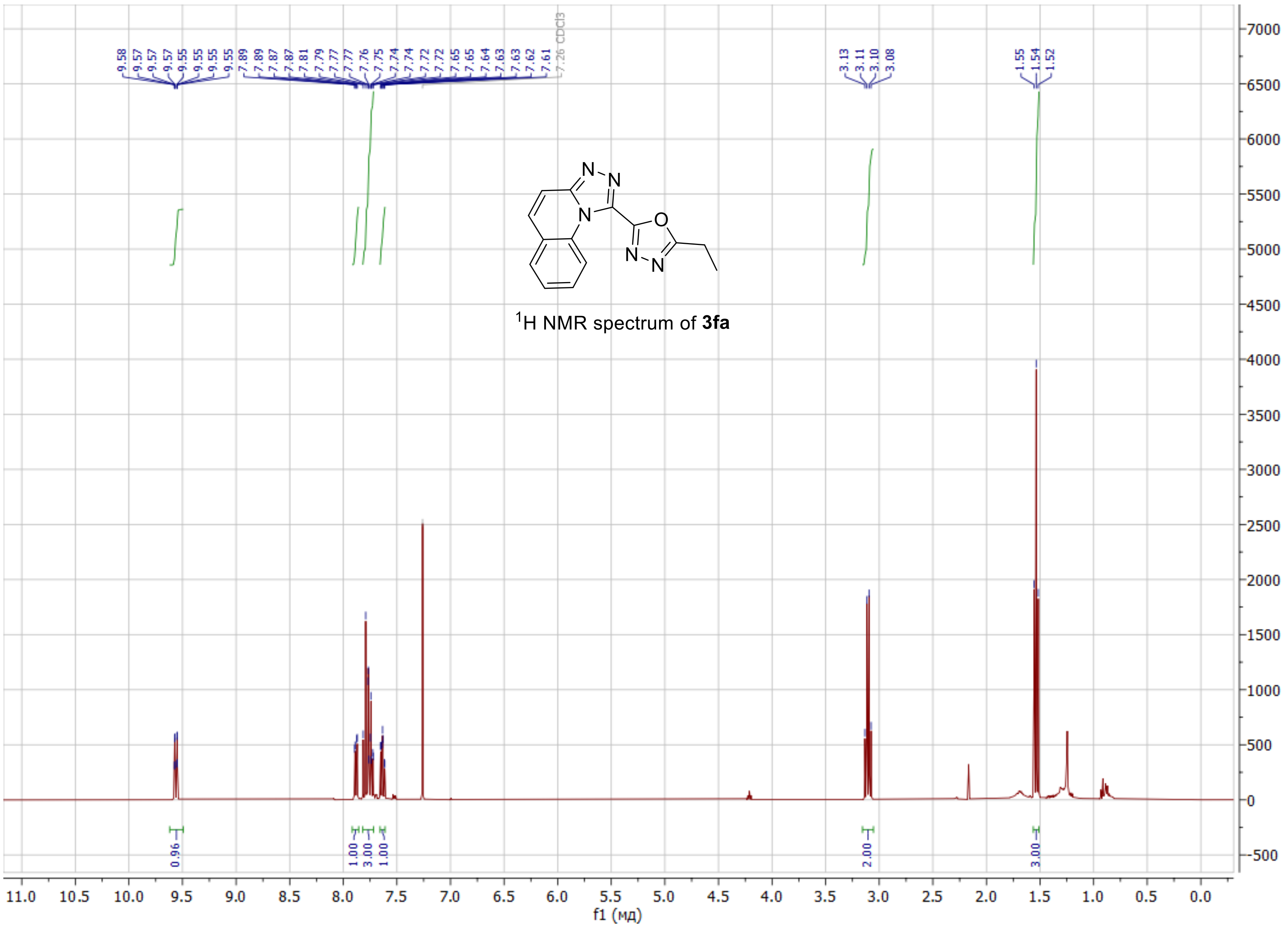

N
N
N
O
N
N
^{1}H NMR spectrum of **3fa**
9.58
9.57
9.57
9.57
9.55
9.55
9.55
9.55
7.89
7.89
7.87
7.87
7.81
7.79
7.77
7.77
7.76
7.75
7.74
7.74
7.72
7.72
7.65
7.65
7.64
7.63
7.63
7.62
7.61
7.26 CDCl3
3.13
3.11
3.10
3.08
1.55
1.54
1.52
0.96
1.00
3.00
1.00
2.00
3.00
f1 (мд)

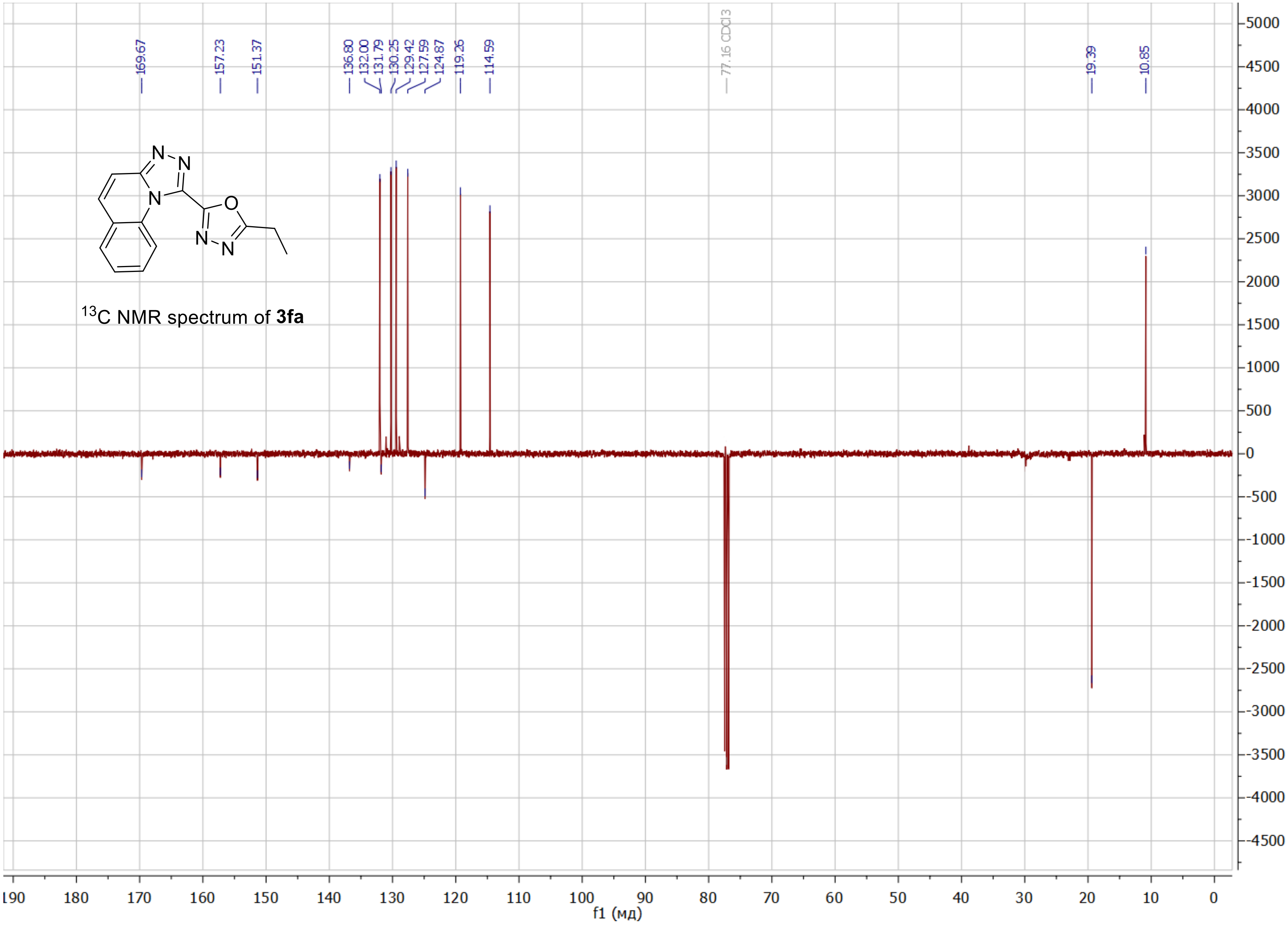

169.67
157.23
151.37
136.80
132.00
131.79
130.25
129.42
127.59
124.87
119.26
114.59
77.16 CDCl3
19.39
10.85
N
N
N
O
N
N
13C NMR spectrum of 3fa
f1 (мд)

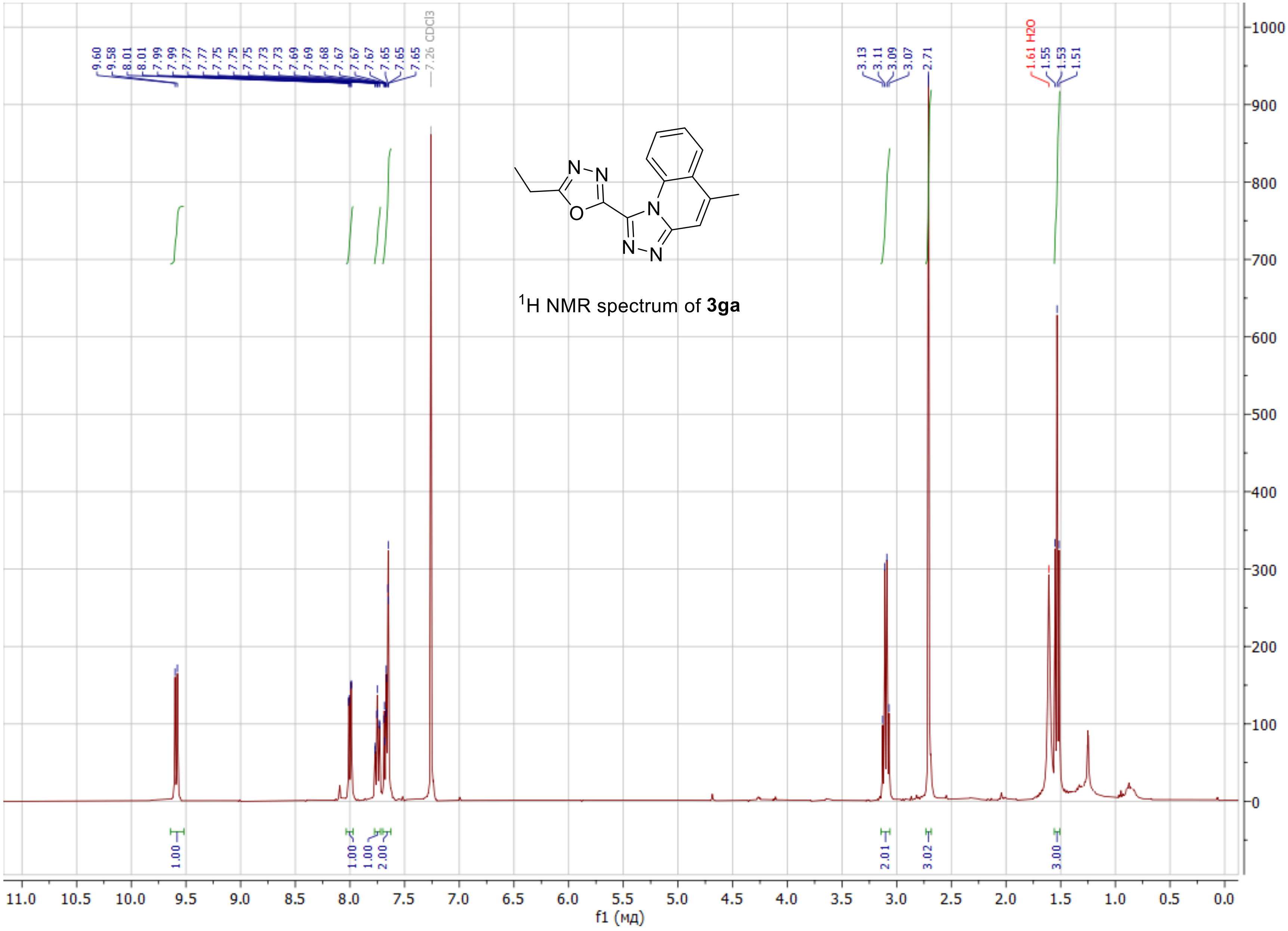

^{1}H NMR spectrum of **3ga**

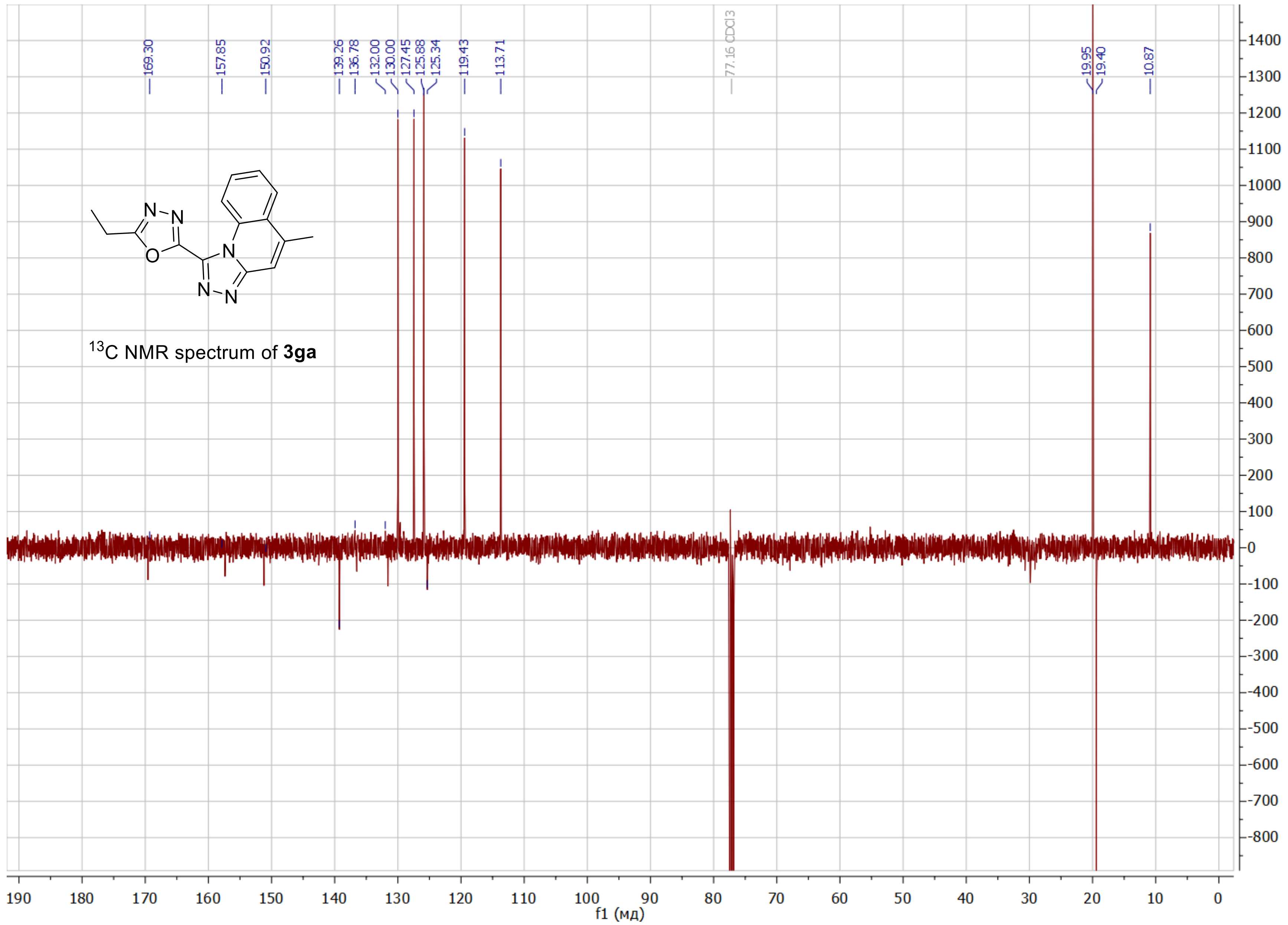

^{13}C NMR spectrum of **3ga**

HRMS spectral charts for starting hydrazineylcarbohydrazides

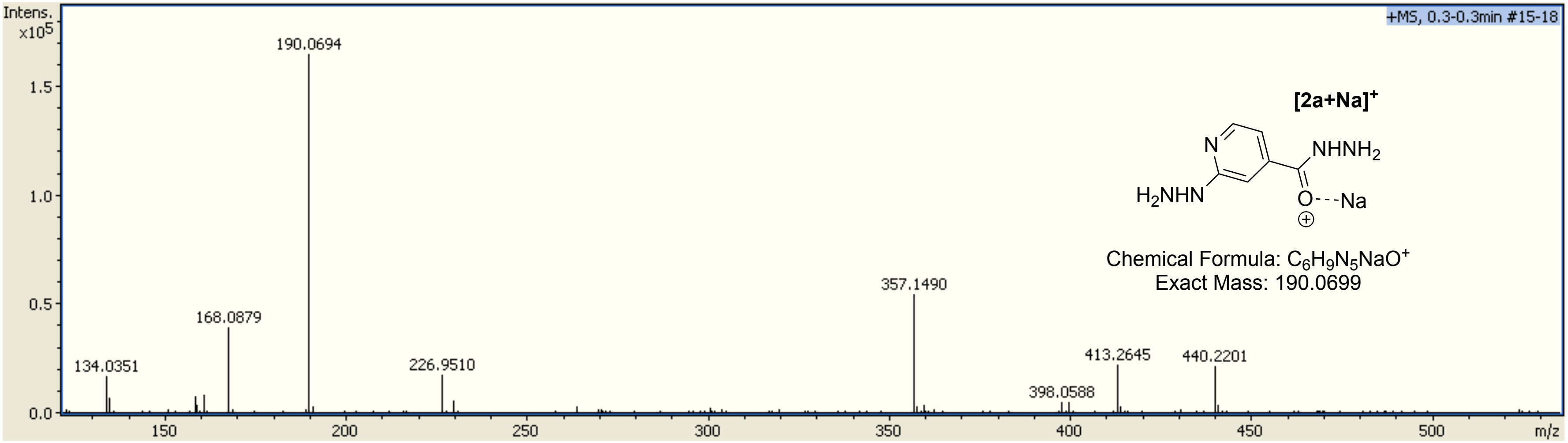

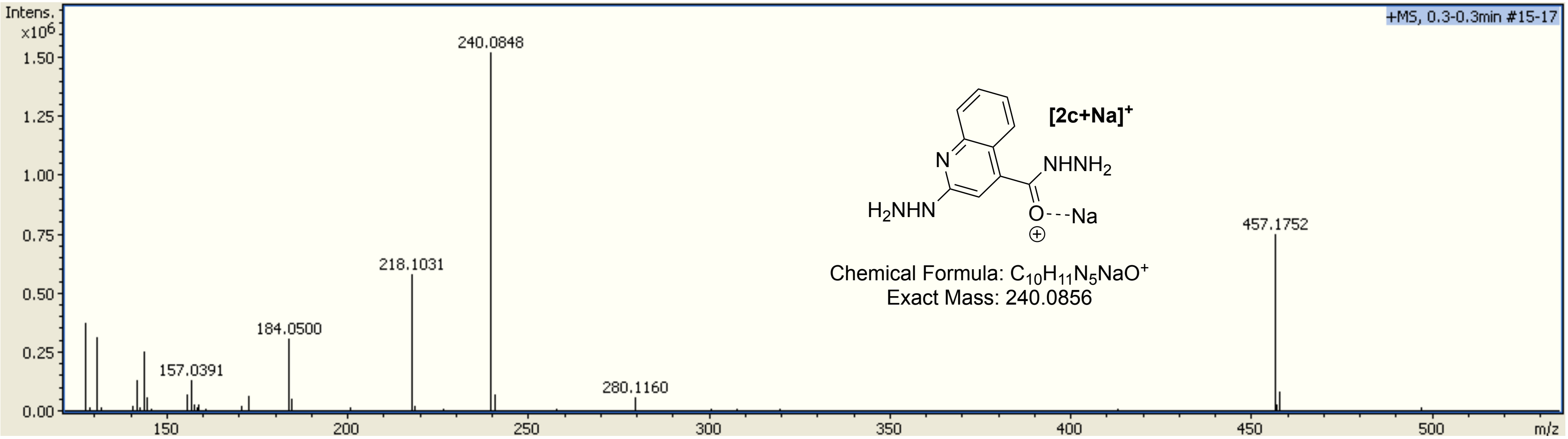

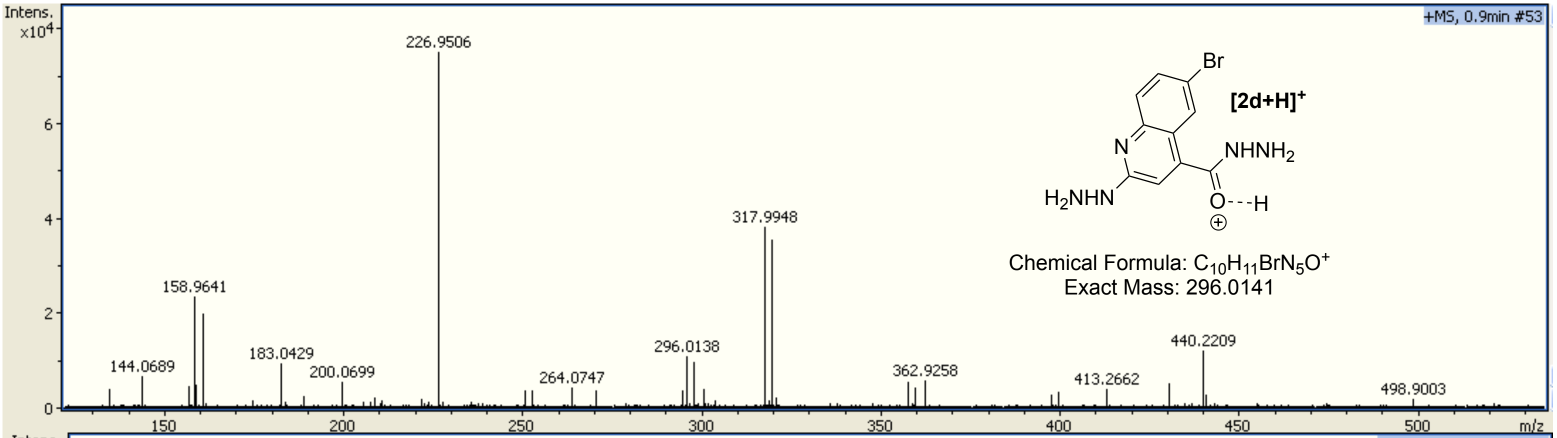

Intens.
x10^4
+MS, 0.9min #53
226.9506
317.9948
158.9641
296.0138
183.0429
144.0689
200.0699
264.0747
362.9258
413.2662
440.2209
498.9003
m/z
Br
[2d+H]+
N
NHNH2
H2NHN
O---H
Chemical Formula: C10H11BrN5O+
Exact Mass: 296.0141

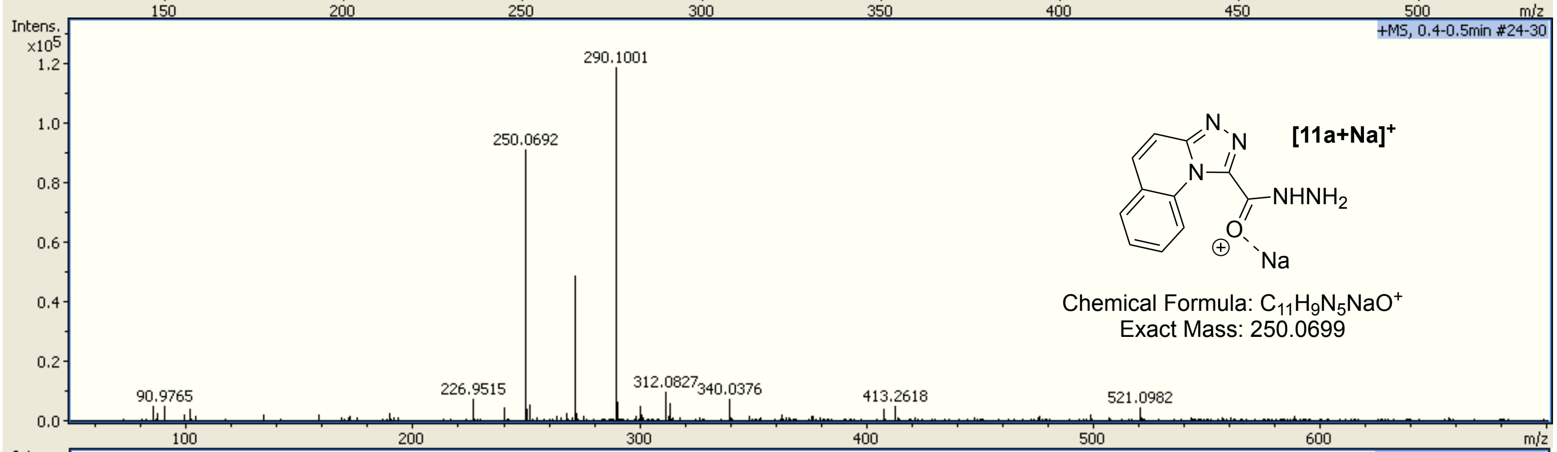

Intens.
x10^5
+MS, 0.4-0.5min #24-30
290.1001
250.0692
90.9765
226.9515
312.0827
340.0376
413.2618
521.0982
m/z
[11a+Na]+
N
N
N
NHNH2
O
Na
Chemical Formula: C11H9N5NaO+
Exact Mass: 250.0699

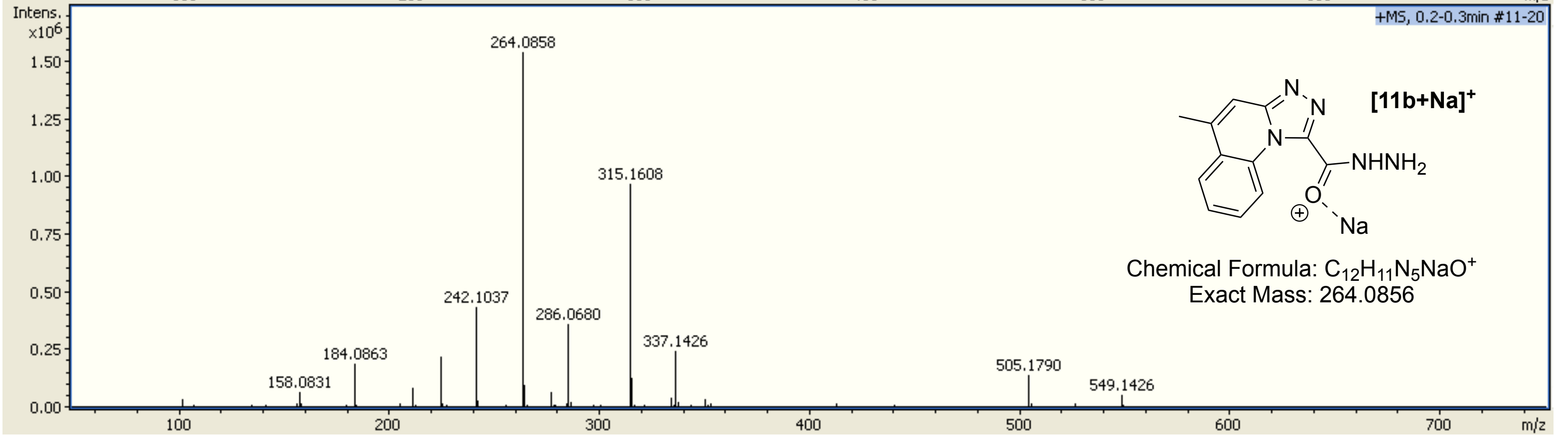

Intens.
x10^6
+MS, 0.2-0.3min #11-20
264.0858
315.1608
242.1037
286.0680
337.1426
184.0863
158.0831
505.1790
549.1426
m/z
[11b+Na]+
N
N
N
NHNH2
O
Na
Chemical Formula: C12H11N5NaO+
Exact Mass: 264.0856

HRMS spectral charts for [1,2,4]triazolo[4,3-a]pyridin-(6)7-yl)-1,3,4-oxadiazoles

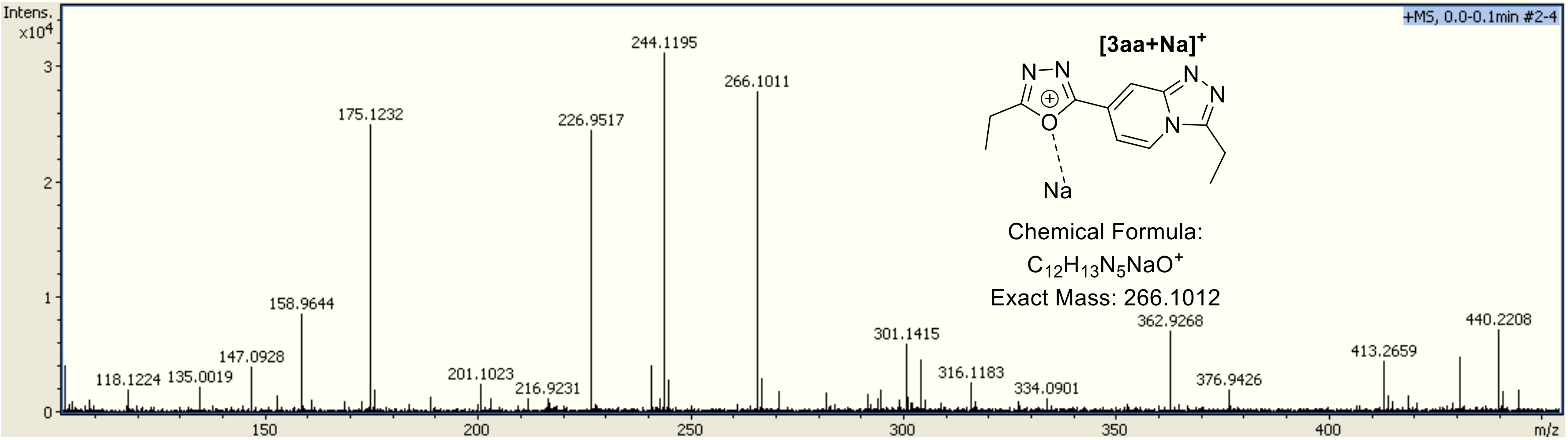

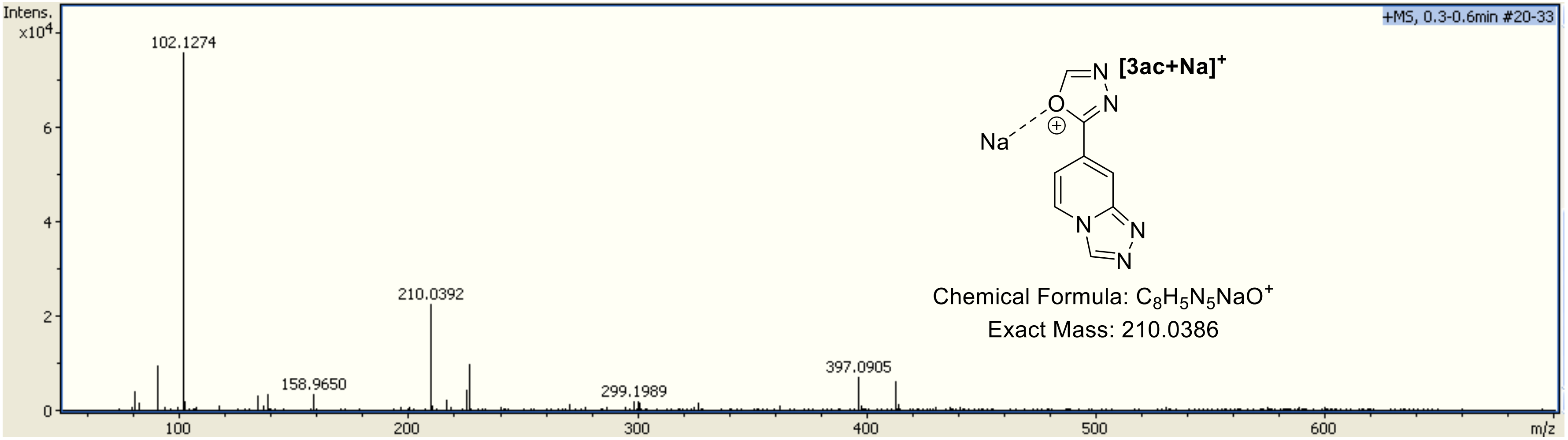

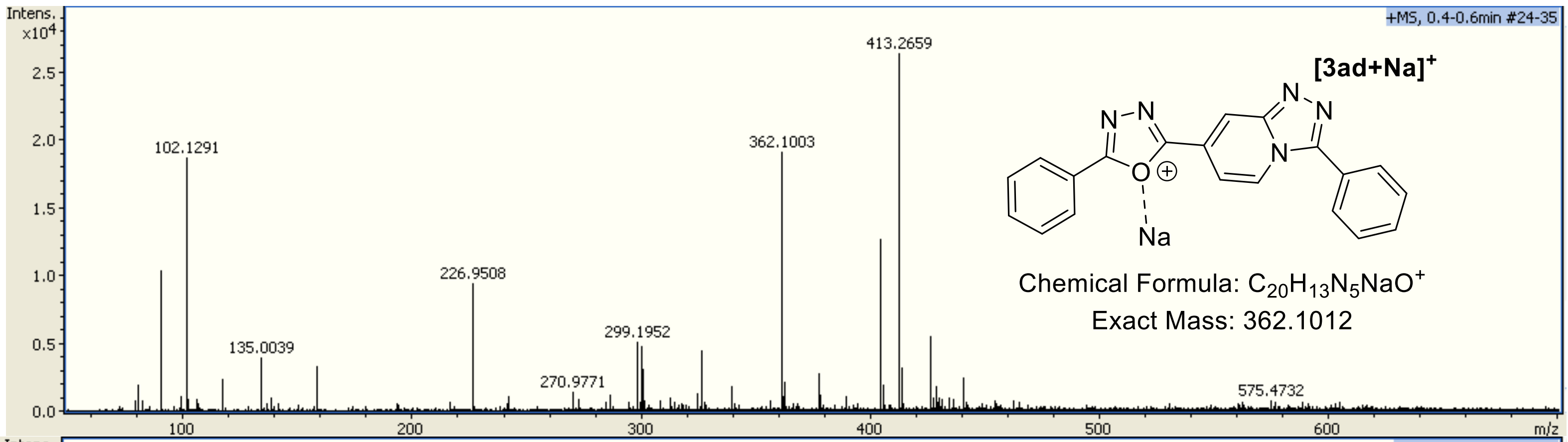
Intens.
x10^4
+MS, 0.4-0.6min #24-35
413.2659
362.1003
102.1291
226.9508
135.0039
299.1952
270.9771
575.4732
m/z
[3ad+Na]+
Na
Chemical Formula: C20H13N5NaO+
Exact Mass: 362.1012

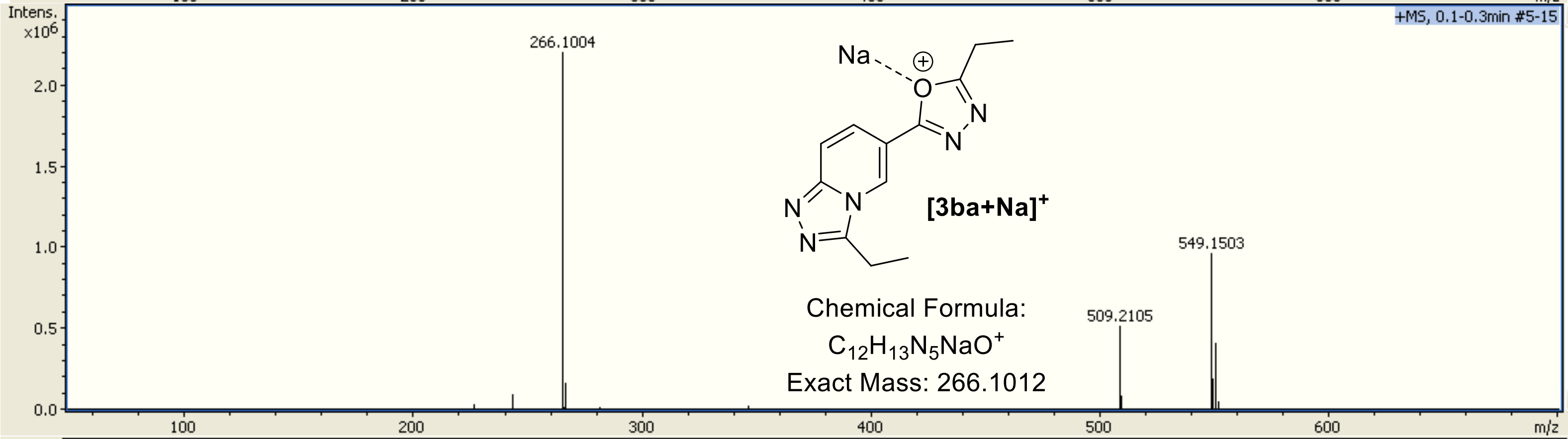
Intens.
x10^6
+MS, 0.1-0.3min #5-15
266.1004
549.1503
509.2105
m/z
[3ba+Na]+
Na
Chemical Formula:
C12H13N5NaO+
Exact Mass: 266.1012

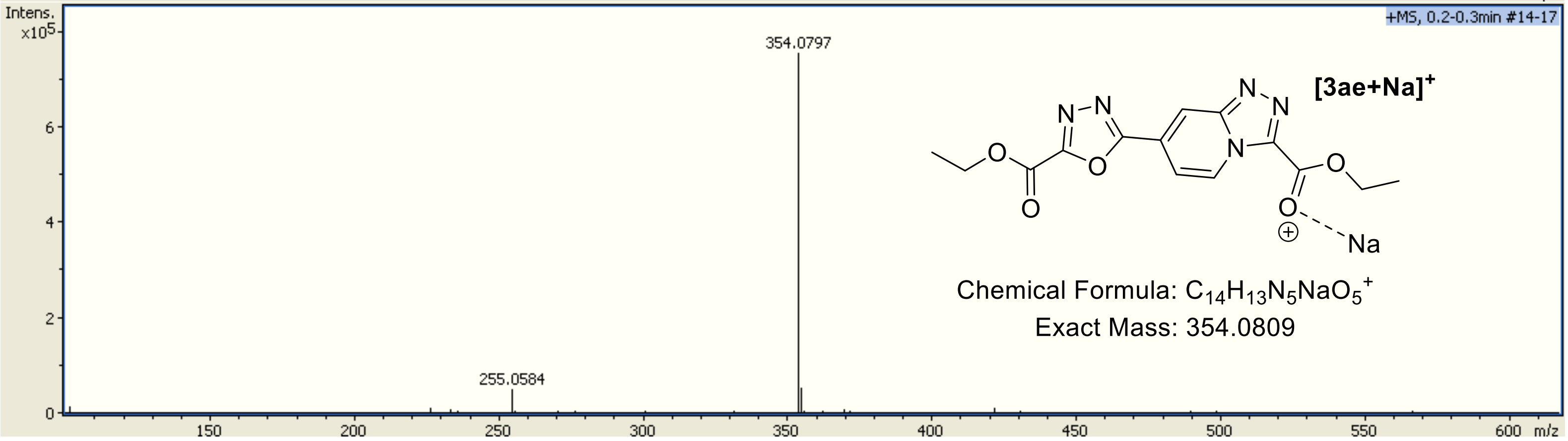
Intens.
x10^5
+MS, 0.2-0.3min #14-17
354.0797
255.0584
m/z
[3ae+Na]+
Na
Chemical Formula: C14H13N5NaO5+
Exact Mass: 354.0809

HRMS spectral charts for (1,3,4-oxadiazol-2-yl)[1,2,4]triazolo[4,3-a]quinolines

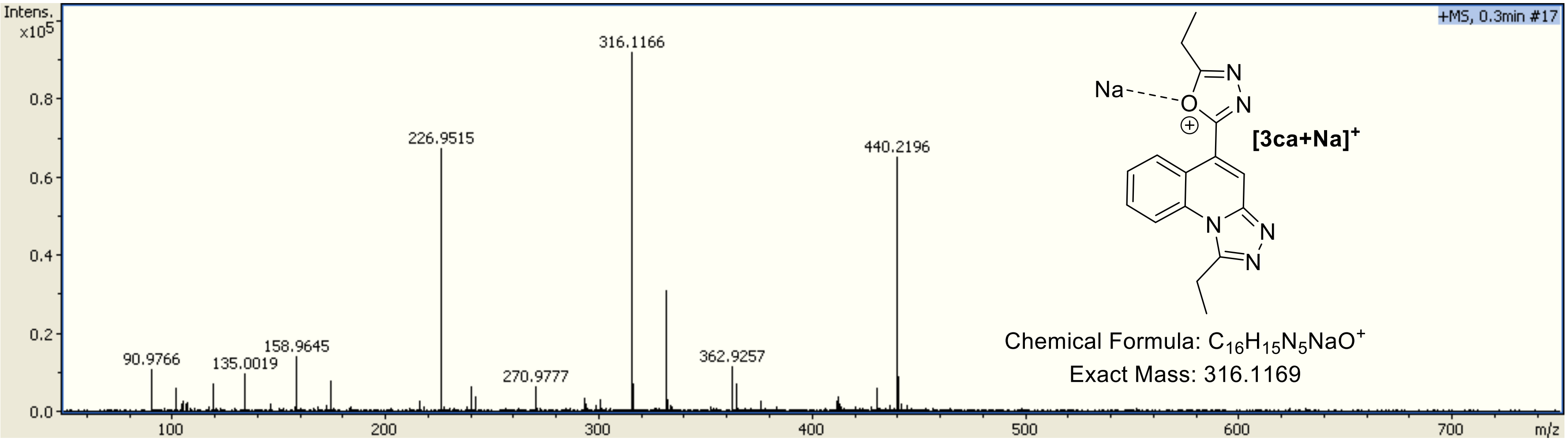

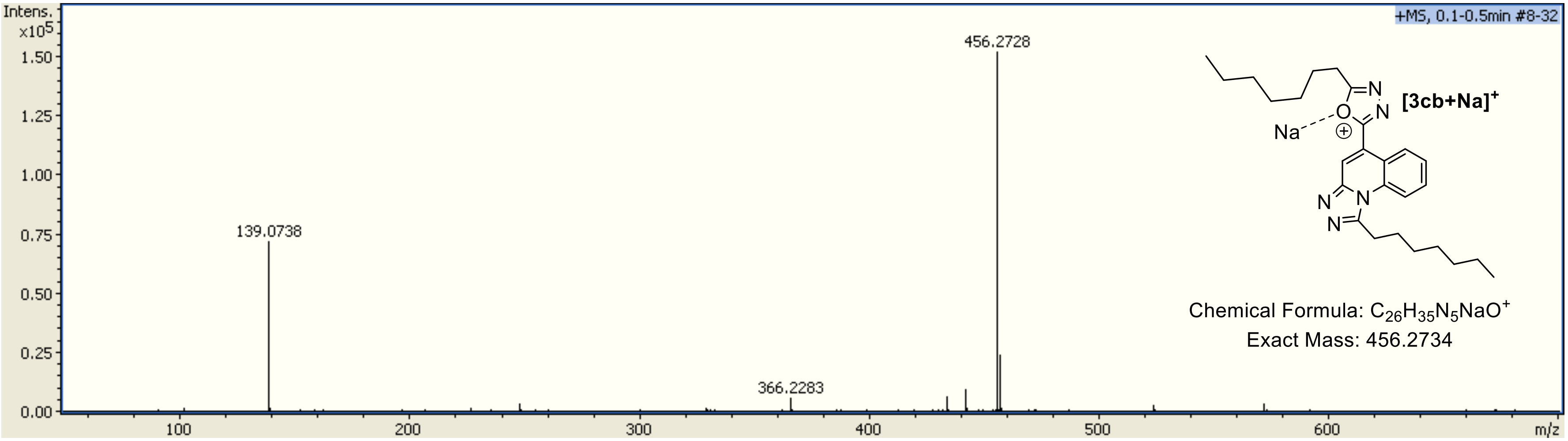

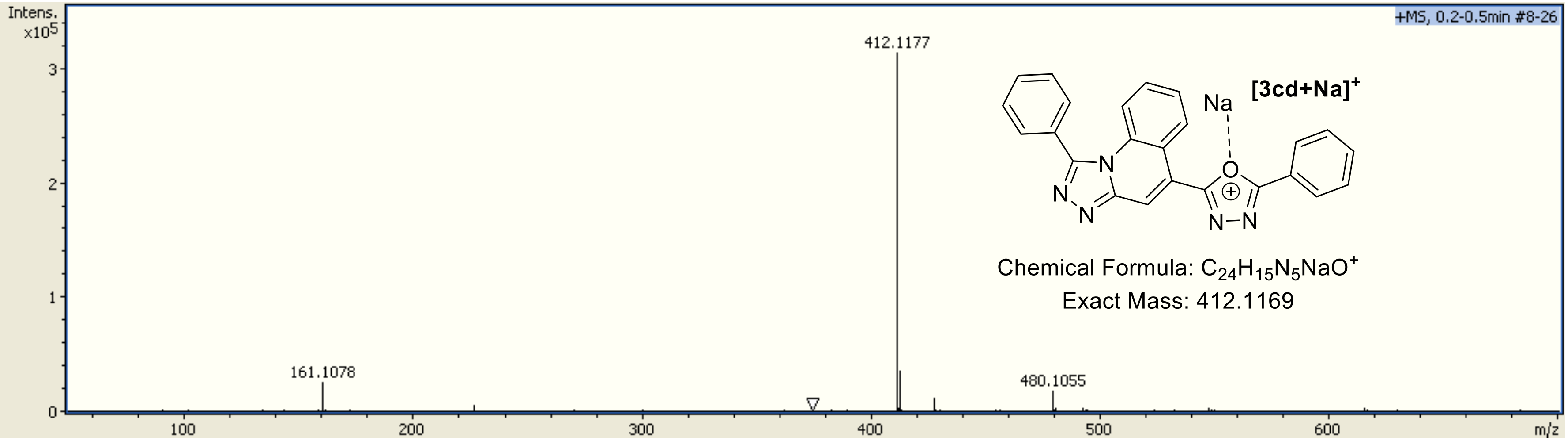
Intens.
x10^5
+MS, 0.2-0.5min #8-26
412.1177
161.1078
480.1055
m/z
[3cd+Na]+
Na
Chemical Formula: C24H15N5NaO+
Exact Mass: 412.1169

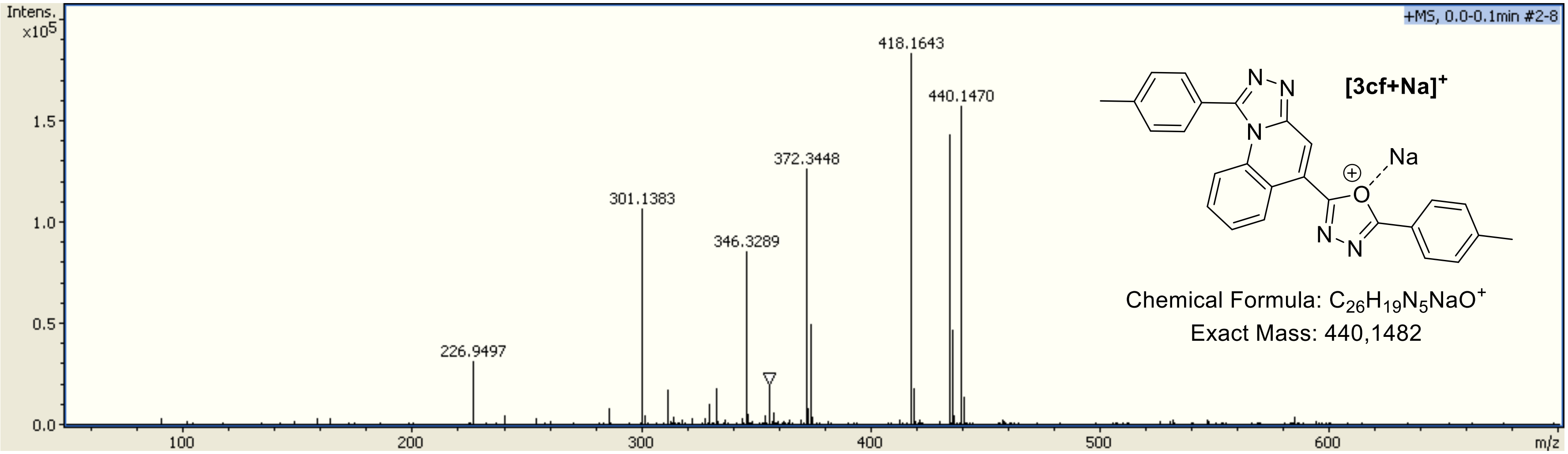
Intens.
x10^5
+MS, 0.0-0.1min #2-8
418.1643
440.1470
372.3448
301.1383
346.3289
226.9497
m/z
[3cf+Na]+
Na
Chemical Formula: C26H19N5NaO+
Exact Mass: 440,1482

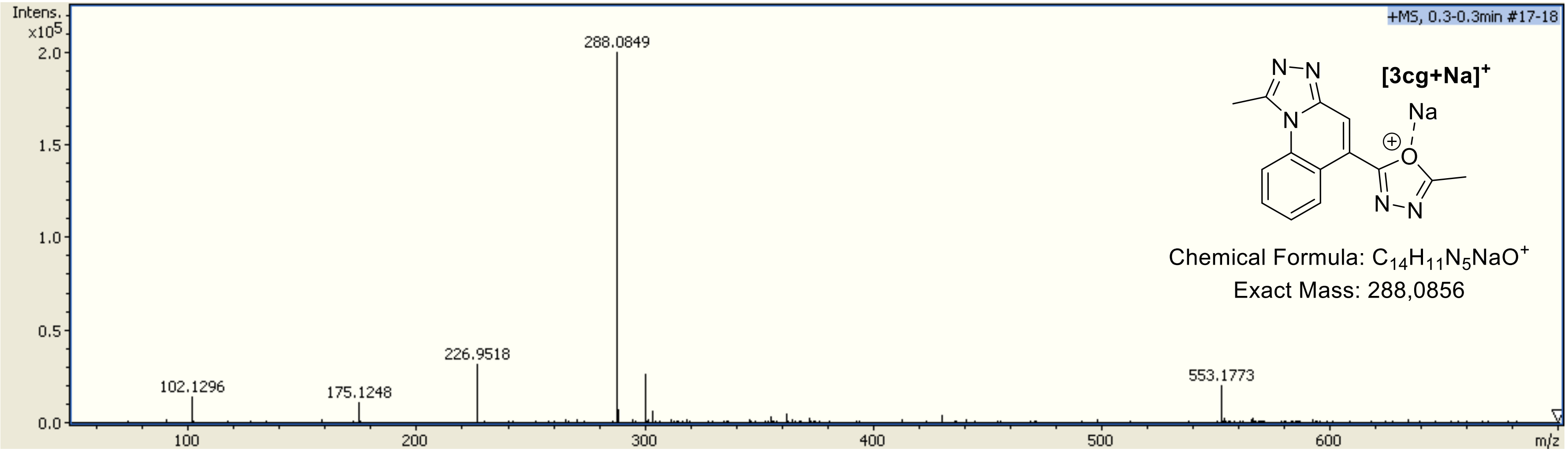

Intens.
x10^5
2.0
1.5
1.0
0.5
0.0
+MS, 0.3-0.3min #17-18
288.0849
102.1296
175.1248
226.9518
553.1773
100
200
300
400
500
600
m/z
[3cg+Na]+
Na
Chemical Formula: $C_{14}H_{11}N_5NaO^+$
Exact Mass: 288,0856

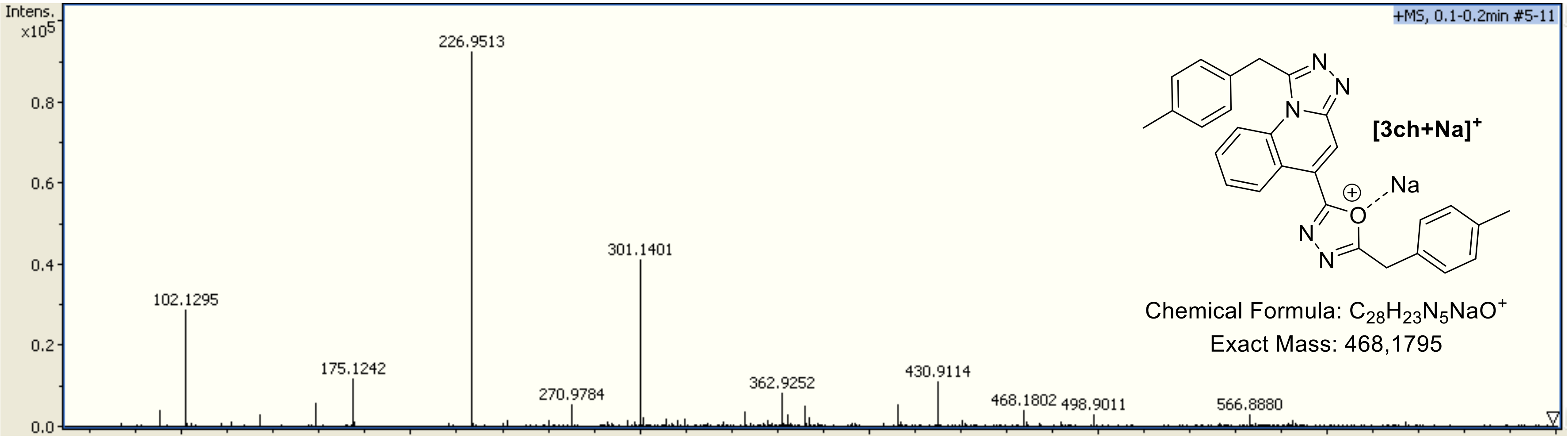

Intens.
x10^5
0.8
0.6
0.4
0.2
0.0
+MS, 0.1-0.2min #5-11
226.9513
301.1401
102.1295
175.1242
270.9784
362.9252
430.9114
468.1802
498.9011
566.8880
[3ch+Na]+
Na
Chemical Formula: $C_{28}H_{23}N_5NaO^+$
Exact Mass: 468,1795

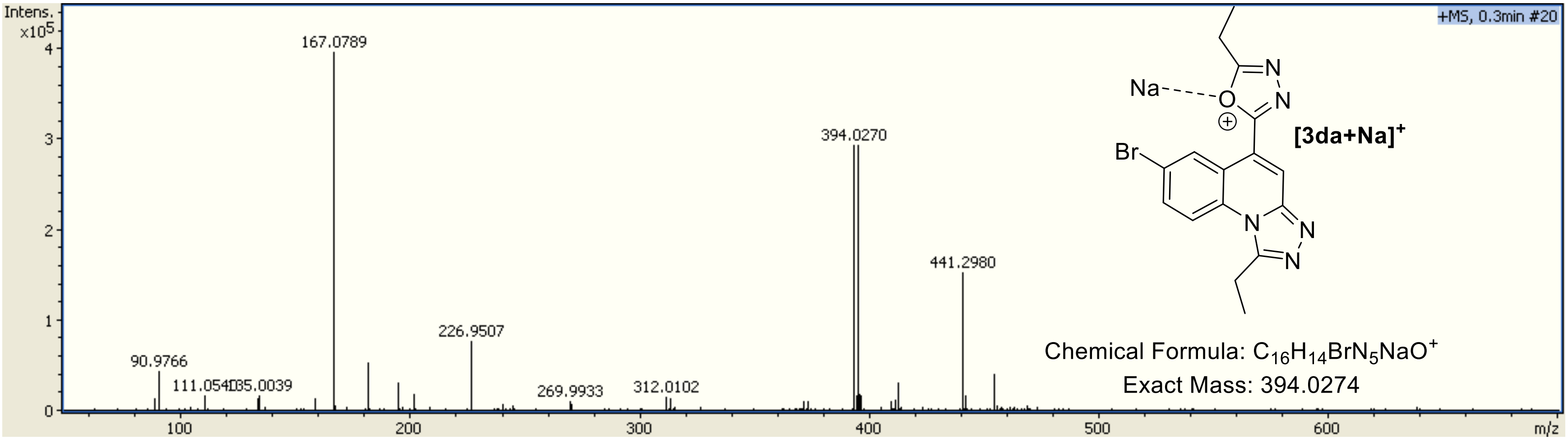
Intens.
x10^5
+MS, 0.3min #20
167.0789
394.0270
441.2980
226.9507
90.9766
111.0540
135.0039
269.9933
312.0102
m/z
Na
O
N
N
Br
N
N
N
[3da+Na]+
Chemical Formula: C16H14BrN5NaO+
Exact Mass: 394.0274

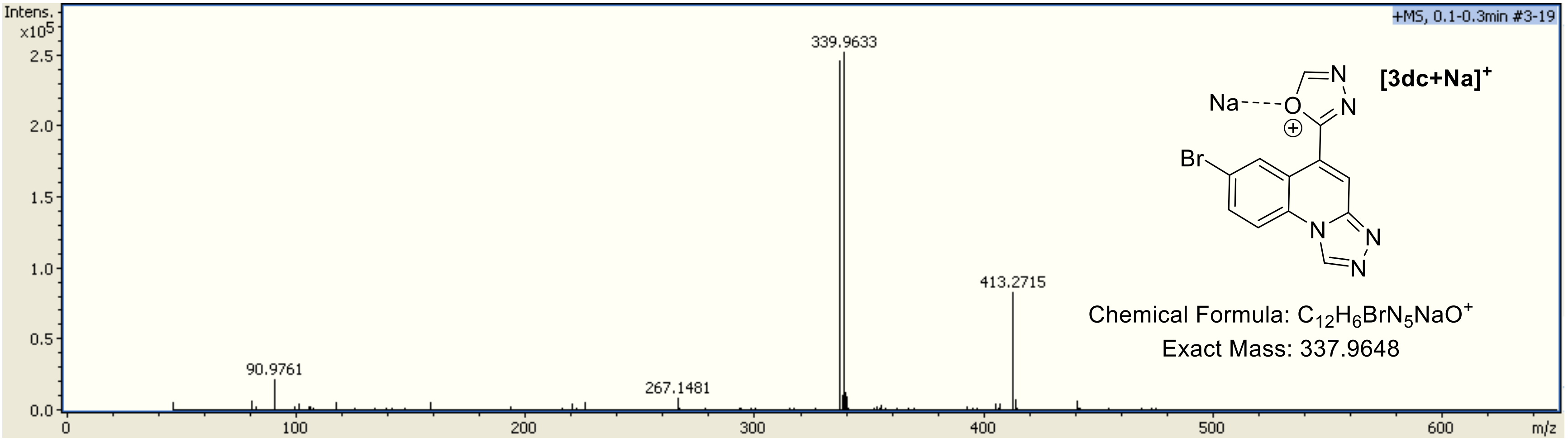
Intens.
x10^5
+MS, 0.1-0.3min #3-19
339.9633
413.2715
90.9761
267.1481
m/z
Na
O
N
N
Br
N
N
N
[3dc+Na]+
Chemical Formula: C12H6BrN5NaO+
Exact Mass: 337.9648

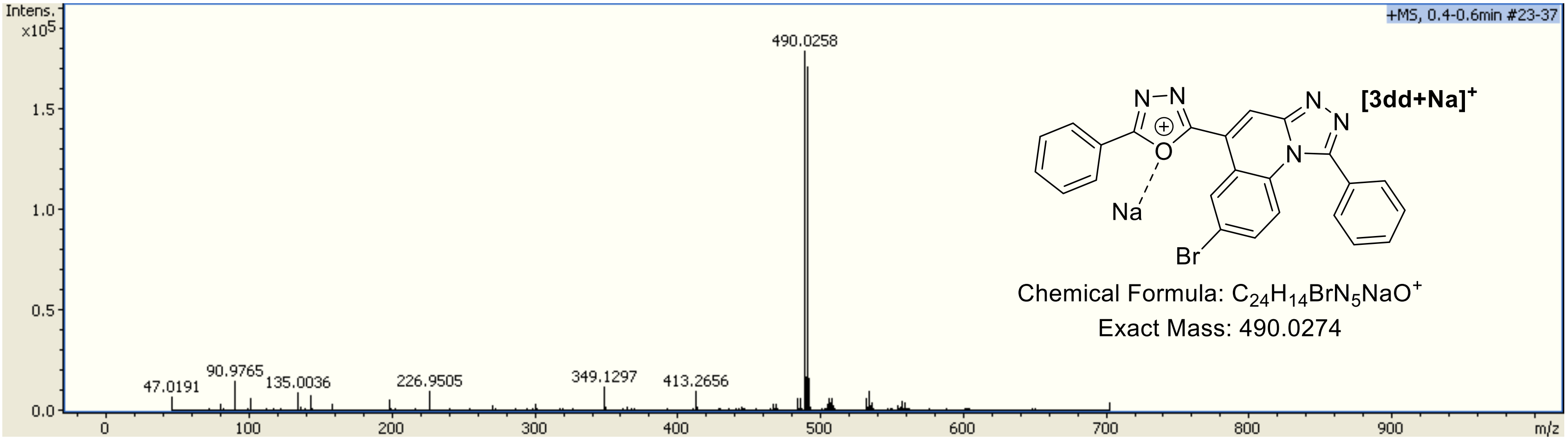
Intens.
x10^5
+MS, 0.4-0.6min #23-37
490.0258
47.0191
90.9765
135.0036
226.9505
349.1297
413.2656
m/z
[3dd+Na]+
Chemical Formula: C24H14BrN5NaO+
Exact Mass: 490.0274

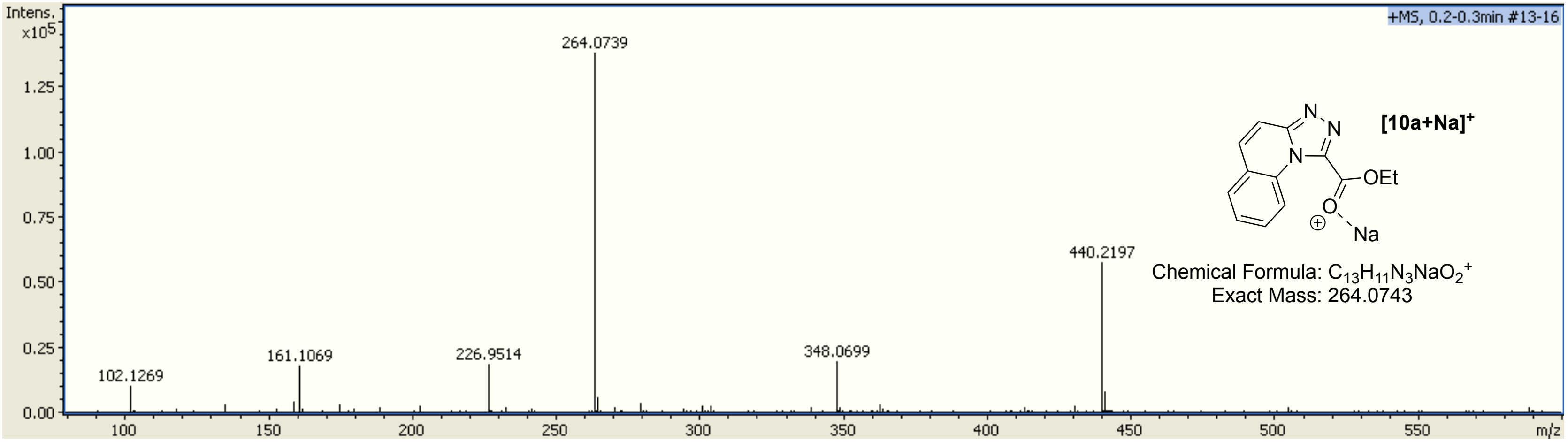
Intens.
x10^5
+MS, 0.2-0.3min #13-16
264.0739
440.2197
102.1269
161.1069
226.9514
348.0699
m/z
[10a+Na]+
Chemical Formula: C13H11N3NaO2+
Exact Mass: 264.0743

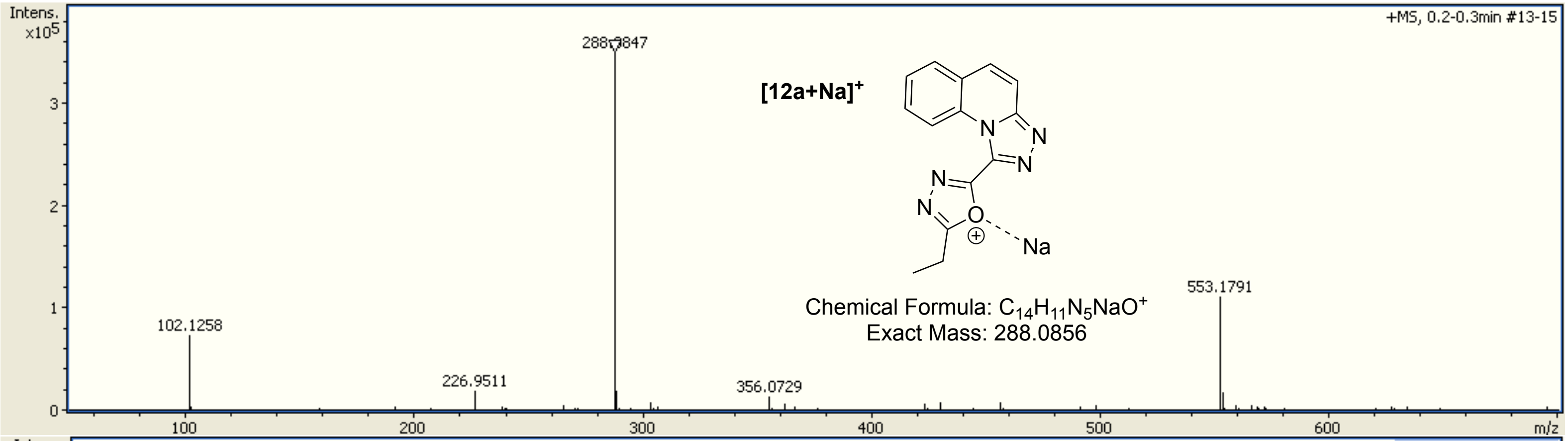
Intens.
x10^5
+MS, 0.2-0.3min #13-15
288.0847
[12a+Na]+
102.1258
226.9511
356.0729
553.1791
Chemical Formula: C14H11N5NaO+
Exact Mass: 288.0856
m/z

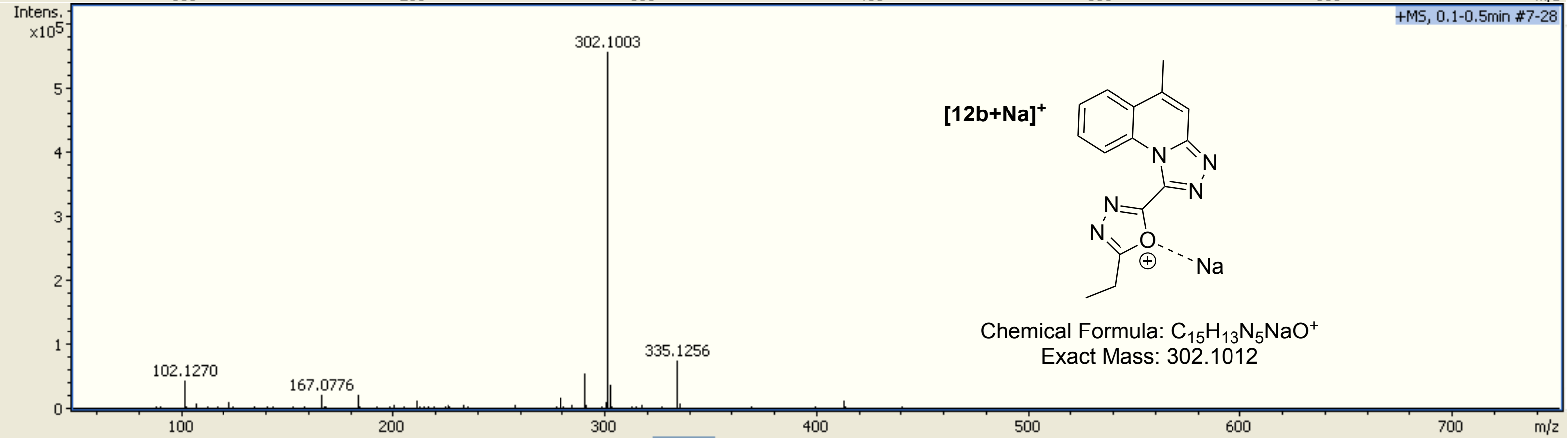
Intens.
x10^5
+MS, 0.1-0.5min #7-28
302.1003
[12b+Na]+
102.1270
167.0776
335.1256
Chemical Formula: C15H13N5NaO+
Exact Mass: 302.1012
m/z

X-Ray crystallography data

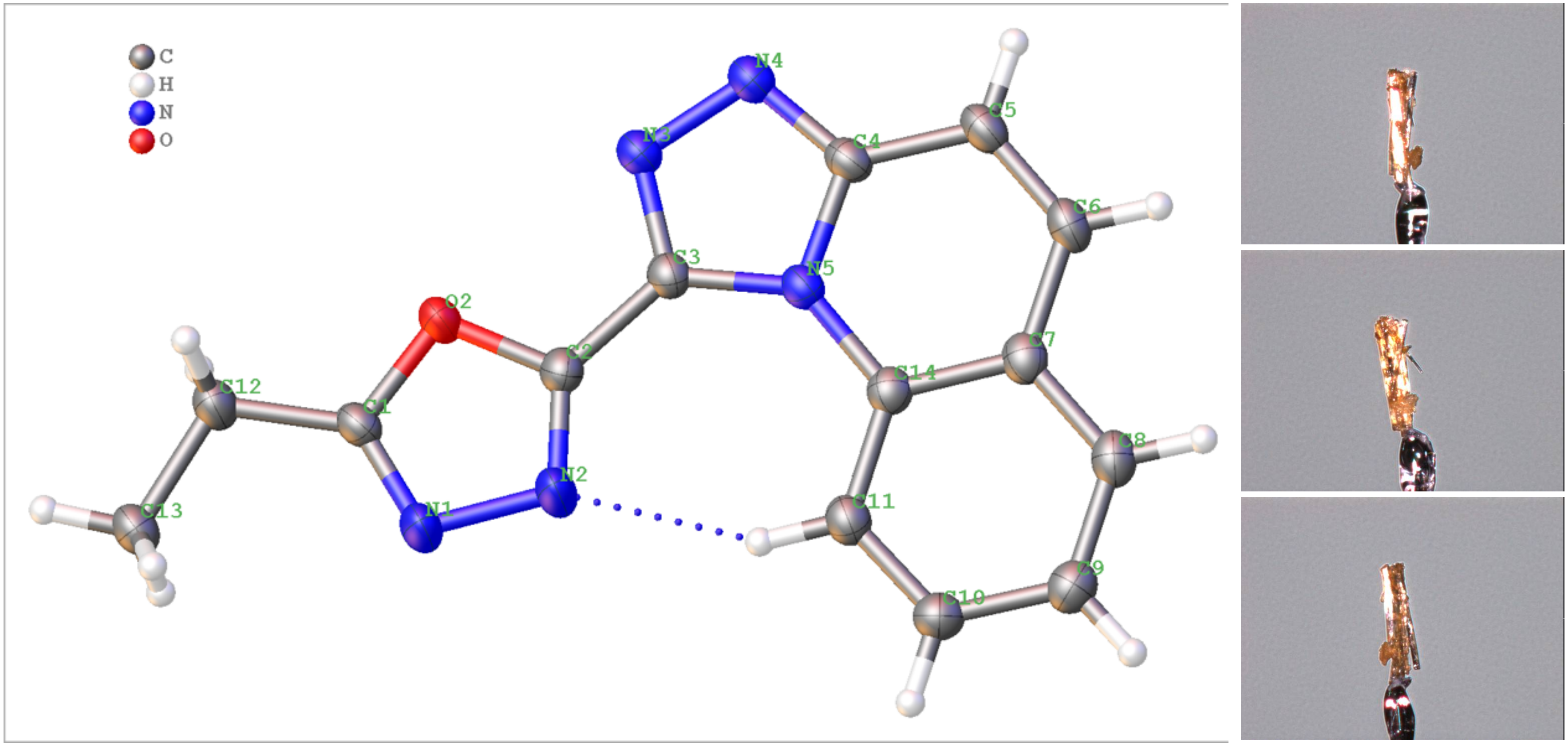

Figure S1. ORTEP drawing of the crystal structure (left) and microphotography of the single crystal of compound **12a** used for X-Ray diffraction analysis (right)

Table S1 Crystal data and structure refinement for 12a.

Identification code	ANNA_NIK424_2
Empirical formula	$C_{14}H_{11}N_5O$
Formula weight	265.28
Temperature/K	100.01(10)
Crystal system	monoclinic
Space group	$P2_1/c$
a/Å	4.14345(14)
b/Å	11.4862(4)
c/Å	24.9424(8)
α/°	90
β/°	90.307(3)
γ/°	90
Volume/Å^3	1187.06(7)
Z	4
ρ_{calc}g/cm^3	1.484
μ/mm^{-1}	0.820
F(000)	552.0
Crystal size/mm^3	0.399 × 0.115 × 0.084
Radiation	Cu Kα (λ = 1.54184)
2Θ range for data collection/°	7.088 to 152.766
Index ranges	-5 ≤ h ≤ 5, -14 ≤ k ≤ 14, -30 ≤ l ≤ 31
Reflections collected	27813
Independent reflections	2489 [R_{int} = 0.1099, R_{sigma} = 0.0345]
Data/restraints/parameters	2489/0/183
Goodness-of-fit on F^2	1.058
Final R indexes [I>=2σ (I)]	R_1 = 0.0516, wR_2 = 0.1256
Final R indexes [all data]	R_1 = 0.0535, wR_2 = 0.1269
Largest diff. peak/hole / e Å^{-3}	0.22/-0.28

Table S2 Fractional Atomic Coordinates (×10^4) and Equivalent Isotropic Displacement Parameters (Å^2×10^3) for 12a. U_{eq} is defined as 1/3 of of the trace of the orthogonalised U_{IJ} tensor.

Atom	*x*	*y*	*z*	U(eq)
O2	2526(4)	1398.2(13)	5178.6(6)	30.9(4)
N4	2246(5)	1623.3(16)	6777.8(7)	29.1(4)
N5	5273(4)	2998.7(15)	6394.7(7)	23.8(4)
N3	2087(5)	1483.0(15)	6235.4(7)	27.5(4)
N2	5764(6)	2890.5(19)	5090.2(8)	46.5(6)
C4	4160(5)	2525.7(18)	6869.7(8)	25.6(4)
C14	7285(5)	3997.3(17)	6395.0(8)	24.5(4)
C7	8097(5)	4478.3(18)	6900.1(8)	26.7(4)
N1	5171(6)	2396.7(18)	4577.8(8)	42.8(6)
C3	3865(5)	2293.9(17)	6006.7(8)	24.3(4)
C11	8406(5)	4514.2(19)	5926.6(9)	27.8(5)
C2	4176(5)	2273.8(17)	5424.4(8)	25.5(4)
C1	3289(5)	1539.5(18)	4651.9(8)	25.7(4)
C8	10053(5)	5474.5(19)	6915.7(9)	29.3(5)
C6	6925(5)	3961.7(19)	7386.8(8)	28.6(5)
C5	5036(6)	3012.0(19)	7375.5(8)	28.4(5)
C10	10342(6)	5488.6(19)	5957.2(9)	29.7(5)
C9	11185(6)	5977.1(19)	6450.9(9)	30.7(5)
C13	2839(6)	982(2)	3684.0(8)	30.8(5)
C12	1955(6)	699.7(19)	4258.7(8)	31.3(5)

Table S3 Anisotropic Displacement Parameters (Å^2×10^3) for 12a. The Anisotropic displacement factor exponent takes the form: -2π^2[h^{2}a*^{2}U$_{11}$+2hka*b*U$_{12}$+…].

Atom	U_{11}	U_{22}	U_{33}	U_{23}	U_{13}	U_{12}
O2	40.4(9)	34.2(8)	18.0(7)	-1.9(6)	2.3(6)	-10.4(7)
N4	36.1(10)	30.0(9)	21.1(8)	0.2(7)	1.2(7)	-2.8(8)
N5	28.2(9)	24.1(8)	19.2(8)	-0.6(6)	1.9(7)	0.6(7)
N3	34.9(10)	27.1(9)	20.6(8)	-0.4(6)	1.5(7)	-3.1(8)
N2	73.2(16)	46.9(12)	19.6(9)	-6.9(8)	6.5(10)	-30.1(12)
C4	29.9(10)	27.1(10)	19.7(10)	1.7(8)	2.3(8)	1.7(9)
C14	25.1(9)	23.8(9)	24.7(10)	-0.9(8)	-0.8(8)	1.4(8)
C7	28.3(10)	25.7(10)	26.0(10)	-2.8(8)	-1.1(9)	3.5(8)
N1	66.8(15)	42.1(11)	19.7(9)	-6.0(8)	6.3(10)	-23.6(11)
C3	28.2(10)	23.4(9)	21.4(10)	-1.4(7)	0.5(8)	0.3(8)
C11	31.5(11)	28.4(10)	23.5(10)	-1.8(8)	-0.7(9)	0.1(9)
C2	31.0(11)	22.5(9)	23.0(10)	-1.6(8)	0.3(8)	-1.0(8)
C1	31.5(11)	27.4(10)	18.3(9)	0.9(8)	2.2(8)	0.1(9)
C8	30.3(11)	30.0(11)	27.7(11)	-5.9(8)	-3.5(9)	2.4(9)
C6	30.9(11)	34.6(11)	20.1(9)	-4.2(8)	-1.4(8)	2.3(9)
C5	34.0(11)	32.9(11)	18.4(9)	-0.4(8)	1.8(9)	3.1(9)
C10	32.7(11)	28.5(11)	27.9(10)	1.8(8)	1.4(9)	-2.4(9)
C9	31.2(11)	27.7(10)	33.1(11)	-1.9(9)	-1.0(9)	-1.7(9)
C13	37.1(12)	34.4(11)	20.9(10)	-1.3(8)	0.6(9)	-1.6(10)
C12	37.3(12)	34.9(11)	21.8(10)	-2.5(9)	-0.1(9)	-6.4(9)

Table S4 Bond Lengths for 12a.

Atom	Atom	Length/Å	Atom	Atom	Length/Å
O2	C2	1.360(3)	C14	C11	1.392(3)
O2	C1	1.363(2)	C7	C8	1.403(3)
N4	N3	1.364(2)	C7	C6	1.438(3)
N4	C4	1.324(3)	N1	C1	1.270(3)
N5	C4	1.385(3)	C3	C2	1.459(3)
N5	C14	1.418(3)	C11	C10	1.379(3)
N5	C3	1.388(3)	C1	C12	1.480(3)
N3	C3	1.319(3)	C8	C9	1.380(3)
N2	N1	1.419(3)	C6	C5	1.343(3)
N2	C2	1.279(3)	C10	C9	1.396(3)
C4	C5	1.425(3)	C13	C12	1.516(3)
C14	C7	1.415(3)			

Table S5 Bond Angles for 12a.

Atom	Atom	Atom	Angle/°	Atom	Atom	Atom	Angle/°
C2	O2	C1	103.17(16)	N5	C3	C2	131.64(19)
C4	N4	N3	106.85(17)	N3	C3	N5	110.13(17)
C4	N5	C14	121.02(17)	N3	C3	C2	118.14(19)
C4	N5	C3	103.13(17)	C10	C11	C14	119.8(2)
C3	N5	C14	135.82(18)	O2	C2	C3	114.43(18)
C3	N3	N4	108.74(17)	N2	C2	O2	112.02(19)
C2	N2	N1	106.17(19)	N2	C2	C3	133.5(2)
N4	C4	N5	111.15(18)	O2	C1	C12	118.25(18)
N4	C4	C5	127.56(19)	N1	C1	O2	112.24(19)
N5	C4	C5	121.28(19)	N1	C1	C12	129.49(19)
C7	C14	N5	116.97(18)	C9	C8	C7	121.1(2)
C11	C14	N5	122.90(19)	C5	C6	C7	121.1(2)
C11	C14	C7	120.12(19)	C6	C5	C4	118.86(19)
C14	C7	C6	120.7(2)	C11	C10	C9	121.2(2)
C8	C7	C14	118.55(19)	C8	C9	C10	119.3(2)
C8	C7	C6	120.72(19)	C1	C12	C13	113.29(18)
C1	N1	N2	106.40(18)				

Table S6 Torsion Angles for 12a.

A	B	C	D	Angle/°	A	B	C	D	Angle/°
O2	C1	C12	C13	177.17(19)	C14	N5	C3	C2	5.8(4)
N4	N3	C3	N5	0.2(2)	C14	C7	C8	C9	-0.3(3)
N4	N3	C3	C2	177.01(19)	C14	C7	C6	C5	0.0(3)
N4	C4	C5	C6	-177.5(2)	C14	C11	C10	C9	-0.6(3)
N5	C4	C5	C6	1.4(3)	C7	C14	C11	C10	0.8(3)
N5	C14	C7	C8	-179.28(19)	C7	C8	C9	C10	0.5(3)
N5	C14	C7	C6	0.3(3)	C7	C6	C5	C4	-0.8(3)
N5	C14	C11	C10	179.6(2)	N1	N2	C2	O2	-0.3(3)
N5	C3	C2	O2	176.4(2)	N1	N2	C2	C3	177.3(2)
N5	C3	C2	N2	-1.1(4)	N1	C1	C12	C13	-4.6(4)
N3	N4	C4	N5	0.4(2)	C3	N5	C4	N4	-0.3(2)
N3	N4	C4	C5	179.4(2)	C3	N5	C4	C5	-179.4(2)
N3	C3	C2	O2	0.4(3)	C3	N5	C14	C7	177.9(2)
N3	C3	C2	N2	-177.1(3)	C3	N5	C14	C11	-1.1(4)
N2	N1	C1	O2	0.0(3)	C11	C14	C7	C8	-0.3(3)
N2	N1	C1	C12	-178.4(2)	C11	C14	C7	C6	179.3(2)
C4	N4	N3	C3	-0.3(2)	C11	C10	C9	C8	-0.1(3)
C4	N5	C14	C7	0.2(3)	C2	O2	C1	N1	-0.1(3)
C4	N5	C14	C11	-178.7(2)	C2	O2	C1	C12	178.4(2)
C4	N5	C3	N3	0.1(2)	C2	N2	N1	C1	0.2(3)
C4	N5	C3	C2	-176.2(2)	C1	O2	C2	N2	0.3(3)
C14	N5	C4	N4	178.03(18)	C1	O2	C2	C3	-177.78(19)
C14	N5	C4	C5	-1.0(3)	C8	C7	C6	C5	179.6(2)
C14	N5	C3	N3	-177.9(2)	C6	C7	C8	C9	-179.9(2)

Table S7 Hydrogen Atom Coordinates (Å×10⁴) and Isotropic Displacement Parameters (Å²×10³) for 12a.

**Atom**	***x***	***y***	***z***	**U(eq)**
H11	7850.91	4203.36	5594.43	33
H8	10597.08	5801.78	7245.1	35
H6	7485.73	4290.87	7714.98	34
H5	4311.97	2677.44	7692.41	34
H10	11099.79	5826.41	5643.3	36
H9	12494.24	6633.94	6466.25	37
H13A	1982.93	393.66	3450.08	46
H13B	5145.53	1007.21	3650.87	46
H13C	1949.04	1725.45	3587.39	46
H12A	-376.84	688.37	4289.8	38
H12B	2744.66	-73.06	4345.53	38

Experimental

Single crystals of $C_{14}H_{11}N_5O$ (**3fa**) were crystallized by slow evaporation of EtOAc solution. A suitable crystal was selected and mounted by acrylic glue on the glass stick on a **SuperNova, Dual, Cu at home/near, AtlasS2** diffractometer. The crystal was kept at 100.01(10) K during data collection. Using Olex2 [S1], the structure was solved with the SHELXT [S2] structure solution program using Intrinsic Phasing and refined with the SHELXL [S3] refinement package using Least Squares minimization.

Crystal structure determination of 3fa

Crystal Data for $C_{14}H_{11}N_5O$ (M =265.28 g/mol): monoclinic, space group $P2_1/c$ (no. 14), a = 4.14345(14) Å, b = 11.4862(4) Å, c = 24.9424(8) Å, β = 90.307(3)°, V = 1187.06(7) Å^3, Z = 4, T = 100.01(10) K, μ(Cu Kα) = 0.820 mm^{-1}, $Dcalc$ = 1.484 g/cm^3, 27813 reflections measured (7.088° ≤ 2Θ ≤ 152.766°), 2489 unique (R_{int} = 0.1099, R_{sigma} = 0.0345) which were used in all calculations. The final R_1 was 0.0516 (I > 2σ(I)) and wR_2 was 0.1269 (all data).

Refinement model description of 3fa

Number of restraints - 0, number of constraints - unknown.

Details:

1. Twinned data refinement
 Scales: 0.8428(18)
 0.1572(18)
2. Fixed Uiso
 At 1.2 times of:
  All C(H) groups, All C(H,H) groups
 At 1.5 times of:
  All C(H,H,H) groups

3.a Secondary CH2 refined with riding coordinates:
 C12(H12A,H12B)

3.b Aromatic/amide H refined with riding coordinates:
 C11(H11), C8(H8), C6(H6), C5(H5), C10(H10), C9(H9)

3.c Idealised Me refined as rotating group:
 C13(H13A,H13B,H13C)

X-Ray crystallography data

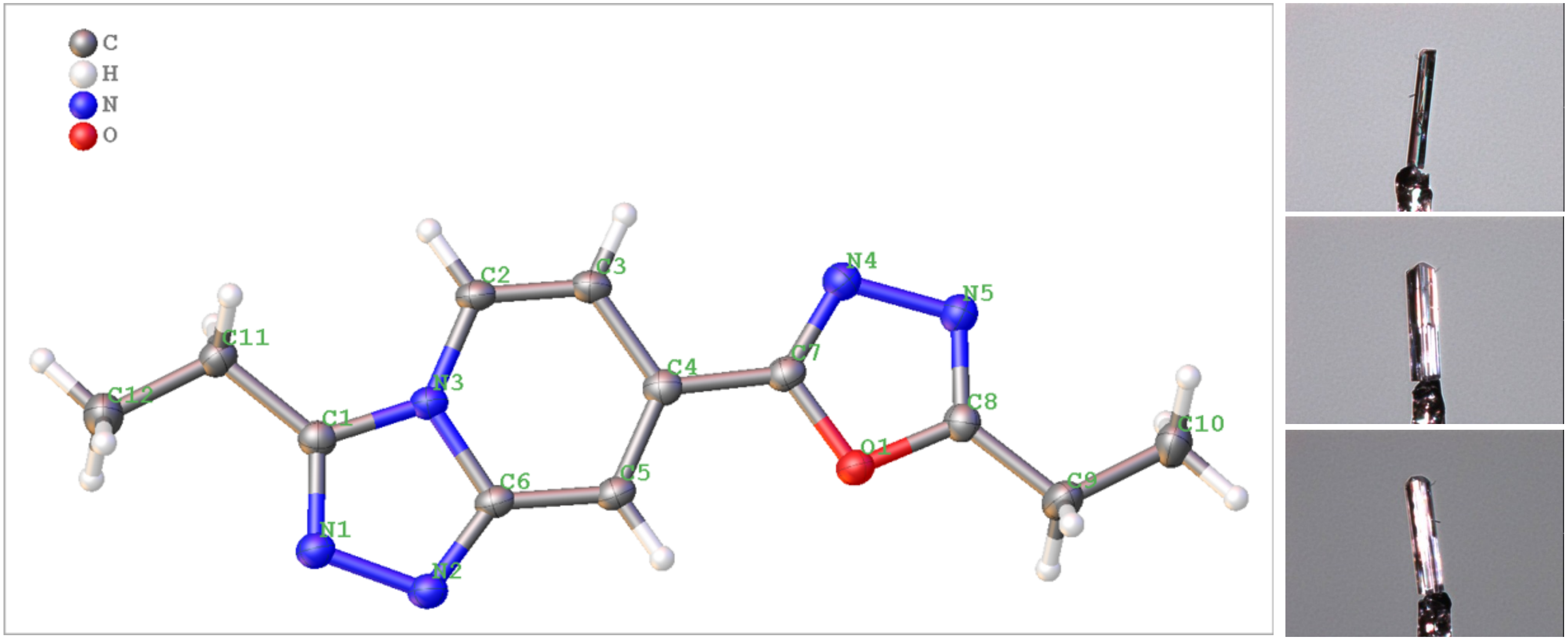

Figure S2. ORTEP drawing of the crystal structure (left) and microphotography of the single crystal of compound **3ba** used for X-Ray diffraction analysis (right)

Table S9 Crystal data and structure refinement for 3ba.

Identification code	ANNA_SOK3_2
Empirical formula	$C_{12}H_{13}N_5O$
Formula weight	243.27
Temperature/K	100.00(10)
Crystal system	monoclinic
Space group	$P2_1/c$
a/Å	14.4997(5)
b/Å	12.4867(4)
c/Å	6.7627(2)
α/°	90
β/°	103.384(4)
γ/°	90
Volume/Å^3	1191.16(7)
Z	4
ρ_{calc}g/cm^3	1.357
μ/mm^{-1}	0.758
F(000)	512.0
Crystal size/mm^3	0.424 × 0.1 × 0.081
Radiation	Cu Kα (λ = 1.54184)
2Θ range for data collection/°	6.266 to 152.694
Index ranges	$-18 \le h \le 17$, $-15 \le k \le 15$, $-5 \le l \le 8$
Reflections collected	12747
Independent reflections	2479 [R_{int} = 0.0256, R_{sigma} = 0.0135]
Data/restraints/parameters	2479/0/166
Goodness-of-fit on F^2	1.079
Final R indexes [I>=2σ (I)]	R_1 = 0.0391, wR_2 = 0.1102
Final R indexes [all data]	R_1 = 0.0405, wR_2 = 0.1129
Largest diff. peak/hole / e Å^{-3}	0.14/-0.30

Table S10 Fractional Atomic Coordinates (×10[4]) and Equivalent Isotropic Displacement Parameters (Å[2]×10[3]) for 3ba. U_{eq} is defined as 1/3 of of the trace of the orthogonalised U_{IJ} tensor.

Atom	*x*	*y*	*z*	U(eq)
O1	8533.2(7)	8287.5(8)	6133.4(15)	21.8(2)
N3	5316.1(8)	6834.5(10)	2803.2(18)	19.0(3)
N2	4965.6(9)	8558.7(10)	2377.6(18)	21.7(3)
N4	8757.6(9)	6544.1(10)	6558.1(19)	23.3(3)
N5	9609.5(9)	7075.5(10)	7477.4(19)	23.4(3)
N1	4164.5(9)	7958.4(10)	1553.6(19)	21.8(3)
C6	5652.2(10)	7873.4(11)	3124(2)	19.0(3)
C1	4379.1(10)	6936.9(11)	1824(2)	20.0(3)
C3	6797.6(10)	6098.6(11)	4336(2)	20.8(3)
C5	6613.2(10)	8039.9(11)	4123(2)	20.2(3)
C8	9443.6(10)	8084.6(12)	7174(2)	21.4(3)
C2	5879.5(10)	5944.8(11)	3400(2)	20.6(3)
C7	8160.2(10)	7284.8(11)	5801(2)	20.2(3)
C11	3742.8(10)	5994.4(11)	1246(2)	23.9(3)
C4	7173.2(10)	7164.3(11)	4728(2)	20.1(3)
C9	10095.4(11)	9009.5(13)	7719(3)	29.9(4)
C12	2753.5(11)	6294.7(13)	65(3)	32.2(4)
C10	11038.8(11)	8698.3(13)	9100(3)	32.0(4)

Table S11 Anisotropic Displacement Parameters (Å^2×10^3) for 3ba. The Anisotropic displacement factor exponent takes the form: $-2\pi^2[h^2a^{*2}U_{11}+2hka^*b^*U_{12}+…]$.

Atom	U_{11}	U_{22}	U_{33}	U_{23}	U_{13}	U_{12}
O1	22.7(5)	18.0(5)	23.3(5)	0.1(4)	2.4(4)	-0.2(4)
N3	21.7(5)	15.9(6)	19.6(5)	0.3(5)	5.1(5)	0.4(4)
N2	24.3(6)	17.5(6)	22.6(6)	0.3(5)	4.1(5)	1.0(4)
N4	22.8(6)	20.9(6)	24.8(6)	-0.7(5)	2.7(5)	0.0(4)
N5	22.2(6)	21.8(6)	24.7(6)	-0.6(5)	2.8(5)	-0.9(5)
N1	23.3(6)	18.8(6)	22.6(6)	0.1(5)	4.1(5)	0.1(4)
C6	24.6(6)	15.0(6)	17.6(6)	-0.7(5)	5.4(5)	-0.2(5)
C1	21.7(6)	19.4(7)	19.2(6)	1.2(5)	5.4(5)	0.4(5)
C3	24.3(7)	16.2(6)	21.9(6)	1.6(5)	5.6(5)	2.5(5)
C5	24.6(7)	16.1(6)	20.3(6)	-1.4(5)	5.9(5)	-1.1(5)
C8	21.5(6)	22.6(7)	19.2(6)	-0.8(5)	3.0(5)	-0.4(5)
C2	25.2(6)	14.2(6)	22.3(6)	-0.3(5)	5.3(5)	1.4(5)
C7	24.0(7)	18.1(7)	18.6(6)	-1.8(5)	5.2(5)	-0.6(5)
C11	23.7(7)	19.1(6)	28.1(7)	0.8(6)	4.5(6)	-2.2(5)
C4	24.0(7)	19.8(6)	16.8(6)	-0.8(5)	5.1(5)	0.4(5)
C9	28.8(7)	20.7(7)	36.4(8)	1.9(6)	0.0(7)	-3.2(6)
C12	24.2(7)	28.7(8)	41.1(9)	2.3(7)	2.2(6)	-1.8(6)
C10	23.3(7)	28.4(8)	41.1(9)	2.0(7)	1.3(7)	-5.2(6)

Table S12 Bond Lengths for 3ba.

Atom	Atom	Length/Å	Atom	Atom	Length/Å
O1	C8	1.3681(17)	N1	C1	1.3156(18)
O1	C7	1.3618(16)	C6	C5	1.416(2)
N3	C6	1.3852(18)	C1	C11	1.4906(19)
N3	C1	1.3727(18)	C3	C2	1.349(2)
N3	C2	1.3827(18)	C3	C4	1.4391(19)
N2	N1	1.3863(17)	C5	C4	1.3670(19)
N2	C6	1.3211(19)	C8	C9	1.484(2)
N4	N5	1.4132(17)	C7	C4	1.4548(19)
N4	C7	1.2894(19)	C11	C12	1.518(2)
N5	C8	1.2903(19)	C9	C10	1.518(2)

Table S13 Bond Angles for 3ba.

Atom	Atom	Atom	Angle/°	Atom	Atom	Atom	Angle/°
C7	O1	C8	102.34(11)	C2	C3	C4	120.56(13)
C1	N3	C6	105.15(11)	C4	C5	C6	118.44(13)
C1	N3	C2	131.88(12)	O1	C8	C9	117.84(13)
C2	N3	C6	122.96(12)	N5	C8	O1	112.64(13)
C6	N2	N1	106.89(12)	N5	C8	C9	129.49(15)
C7	N4	N5	106.04(12)	C3	C2	N3	118.35(13)
C8	N5	N4	106.06(13)	O1	C7	C4	118.99(12)
C1	N1	N2	108.61(12)	N4	C7	O1	112.91(12)
N3	C6	C5	118.94(12)	N4	C7	C4	128.08(13)
N2	C6	N3	109.88(12)	C1	C11	C12	113.27(12)
N2	C6	C5	131.18(13)	C3	C4	C7	118.31(13)
N3	C1	C11	122.44(12)	C5	C4	C3	120.74(13)
N1	C1	N3	109.46(12)	C5	C4	C7	120.95(13)
N1	C1	C11	128.09(13)	C8	C9	C10	112.66(13)

Table S14 Torsion Angles for 3ba.

A	B	C	D	Angle/˚	A	B	C	D	Angle/˚
O1	C8	C9	C10	172.46(13)	C6	N3	C2	C3	-0.1(2)
O1	C7	C4	C3	178.18(12)	C6	N2	N1	C1	0.28(15)
O1	C7	C4	C5	-2.4(2)	C6	C5	C4	C3	0.8(2)
N3	C6	C5	C4	0.1(2)	C6	C5	C4	C7	-178.61(12)
N3	C1	C11	C12	-176.51(13)	C1	N3	C6	N2	-0.36(16)
N2	N1	C1	N3	-0.51(16)	C1	N3	C6	C5	179.96(12)
N2	N1	C1	C11	178.17(13)	C1	N3	C2	C3	179.31(14)
N2	C6	C5	C4	-179.48(14)	C8	O1	C7	N4	0.69(15)
N4	N5	C8	O1	0.83(17)	C8	O1	C7	C4	179.25(12)
N4	N5	C8	C9	-176.79(15)	C2	N3	C6	N2	179.16(12)
N4	C7	C4	C3	-3.5(2)	C2	N3	C6	C5	-0.5(2)
N4	C7	C4	C5	175.94(14)	C2	N3	C1	N1	-178.92(13)
N5	N4	C7	O1	-0.23(16)	C2	N3	C1	C11	2.3(2)
N5	N4	C7	C4	-178.63(13)	C2	C3	C4	C5	-1.4(2)
N5	C8	C9	C10	-10.0(2)	C2	C3	C4	C7	178.01(13)
N1	N2	C6	N3	0.06(15)	C7	O1	C8	N5	-0.94(16)
N1	N2	C6	C5	179.69(14)	C7	O1	C8	C9	176.98(13)
N1	C1	C11	C12	5.0(2)	C7	N4	N5	C8	-0.36(16)
C6	N3	C1	N1	0.53(16)	C4	C3	C2	N3	1.0(2)
C6	N3	C1	C11	-178.24(12)					

Table S15 Hydrogen Atom Coordinates (Å×10[4]) and Isotropic Displacement Parameters (Å[2]×10[3]) for 3ba.

Atom	*x*	*y*	*z*	U(eq)
H3	7191.18	5510.91	4730.31	25
H5	6857.58	8728.72	4360.51	24
H2	5631.55	5257.42	3163.07	25
H11A	3695.2	5614.66	2469.67	29
H11B	4023.95	5511.14	427.87	29
H9A	9795.15	9547.41	8394.18	36
H9B	10207.67	9325.31	6485.79	36
H12A	2790.15	6623.94	-1197.07	48
H12B	2476.12	6787.93	850	48
H12C	2368.69	5661.94	-203.84	48
H10A	11340.94	8167.93	8438.6	48
H10B	10934.44	8410.94	10347.17	48
H10C	11437.81	9319.18	9389.11	48

Experimental

Single crystals of $C_{12}H_{13}N_5O$ (**3ba**) were crystallized by slow evaporation of saturated solution in EtOAc. A suitable crystal was selected and mounted by acrylic glue on the glass stick on a **SuperNova, Dual, Cu at home/near, AtlasS2** diffractometer. The crystal was kept at 100.00(10) K during data collection. Using Olex2 [S1], the structure was solved with the SHELXT [S2] structure solution program using Intrinsic Phasing and refined with the SHELXL [S3] refinement package using Least Squares minimization.

Crystal structure determination of 3ba

Crystal Data for $C_{12}H_{13}N_5O$ (M =243.27 g/mol): monoclinic, space group $P2_1/c$ (no. 14), a = 14.4997(5) Å, b = 12.4867(4) Å, c = 6.7627(2) Å, β = 103.384(4)°, V = 1191.16(7) Å^3, Z = 4, T = 100.00(10) K, μ(Cu Kα) = 0.758 mm^{-1}, $Dcalc$ = 1.357 g/cm^3, 12747 reflections measured (6.266° ≤ 2Θ ≤ 152.694°), 2479 unique (R_{int} = 0.0256, R_{sigma} = 0.0135) which were used in all calculations. The final R_1 was 0.0391 (I > 2σ(I)) and wR_2 was 0.1129 (all data).

Refinement model description

Number of restraints - 0, number of constraints - unknown.
Details:
1. Twinned data refinement
 Scales: 0.7827(13)
 0.2173(13)
2. Fixed Uiso
 At 1.2 times of:
  All C(H) groups, All C(H,H) groups
 At 1.5 times of:
  All C(H,H,H) groups
3.a Secondary CH2 refined with riding coordinates:
 C11(H11A,H11B), C9(H9A,H9B)
3.b Aromatic/amide H refined with riding coordinates:
 C3(H3), C5(H5), C2(H2)
3.c Idealised Me refined as rotating group:
 C12(H12A,H12B,H12C), C10(H10A,H10B,H10C)

This report has been created with Olex2, compiled on 2020.11.12 svn.r5f609507 for OlexSys. Please let us know if there are any errors or if you would like to have additional features.

References

(S1) Dolomanov, O. V.; Bourhis, L. J.; Gildea, R. J.; Howard, J. A. K.; Puschmann, H. OLEX 2: A Complete Structure Solution, Refinement and Analysis Program. *J. Appl. Cryst*. **2009**, 42, 339-341.

(S2) Sheldrick, G. M. SHELXT – Integrated Space-Group and Crystal-Structure Determination. *Acta Cryst*. **2015**, A71, 3-8.

(S3) Sheldrick, G. M. Crystal Structure Refinement with SHELXL. *Acta Cryst*. **2015**, C71, 3-8
